# Effectiveness of Predominantly Group Schema Therapy and Combined Individual and Group Schema Therapy for Borderline Personality Disorder: A Randomized Clinical Trial

**DOI:** 10.1001/jamapsychiatry.2022.0010

**Published:** 2022-03-02

**Authors:** Arnoud Arntz, Gitta A. Jacob, Christopher W. Lee, Odette Manon Brand-de Wilde, Eva Fassbinder, R. Patrick Harper, Anna Lavender, George Lockwood, Ioannis A. Malogiannis, Florian A. Ruths, Ulrich Schweiger, Ida A. Shaw, Gerhard Zarbock, Joan M. Farrell

**Affiliations:** 1 Department of Clinical Psychology, University of Amsterdam, Amsterdam, the Netherlands; 2 Department of Clinical Psychology and Psychotherapy, Institute for Psychology, University of Freiburg, Freiburg, Germany; 3 Faculty of Health and Medical Sciences, University of Western Australia, Perth, Western Australia, Australia; 4 De Viersprong, the Netherlands Institute for Personality Disorders, Halsteren, the Netherlands; 5 Department of Psychiatry and Psychotherapy, University of Lübeck, Lübeck, Germany; 6 Department of Psychiatry and Psychotherapy, Christian-Albrechts University of Kiel, Kiel, Germany; 7 Bradford District Care NHS Foundation Trust, Bradford, United Kingdom; 8 South London and Maudsley NHS Foundation Trust, London, United Kingdom; 9 Schema Therapy Institute Midwest, Kalamazoo, Michigan; 10 First Department of Psychiatry, Eginition Hospital, Medical School, Athens University, Athens, Greece; 11 Schema Therapy Institute Midwest, Indianapolis, Indiana; 12 Institut für Verhaltenstherapie Ausbildung Hamburg GmbH (Institute for Training in Cognitive Behavioral Therapy), Hamburg, Germany; 13 Department of Clinical Psychology, Indiana University–Purdue University, Indianapolis

## Abstract

**Importance:**

Schema therapy (ST), delivered either in an individual or group format, has been compared with other active treatments for borderline personality disorder (BPD). To our knowledge, the 2 formats have not been compared with treatment as usual (TAU) or with each other. Such comparisons help determine best treatment practices.

**Objective:**

To evaluate whether ST is more effectively delivered in a predominantly group or combined individual and group format and whether ST is more effective than optimal TAU for BPD.

**Design, Setting, and Participants:**

In this multicenter, 3-arm randomized clinical trial conducted at 15 sites in 5 countries (Australia, Germany, Greece, the Netherlands, and the UK), outpatients aged 18 to 65 years who had BPD were recruited between June 29, 2010, and May 18, 2016, to receive either predominantly group ST (PGST), combined individual and group ST (IGST), or optimal TAU. Data were analyzed from June 4, 2019, to December 29, 2021.

**Interventions:**

At each site, cohorts of 16 to 18 participants were randomized 1:1 to PGST vs TAU or IGST vs TAU. Both ST formats were delivered over 2 years, with 2 sessions per week in year 1 and the frequency gradually decreasing during year 2. Assessments were collected by blinded assessors.

**Main Outcomes and Measures:**

The primary outcome was the change in BPD severity over time, assessed with the Borderline Personality Disorder Severity Index (BPDSI) total score. Treatment retention was analyzed as a secondary outcome using generalized linear mixed model survival analysis.

**Results:**

Of 495 participants (mean [SD] age, 33.6 [9.4] years; 426 [86.2%] female), 246 (49.7%) received TAU, 125 (25.2%) received PGST, and 124 (25.0%) received IGST (1 of whom later withdrew consent). PGST and IGST combined were superior to TAU with regard to reduced BPD severity (Cohen *d*, 0.73; 95% CI, 0.29-1.18; *P* < .001). For this outcome, IGST was superior to TAU (Cohen *d*, 1.14; 95% CI, 0.57-1.71; *P* < .001) and PGST (Cohen *d*, 0.84; 95% CI, 0.09-1.59; *P* = .03), whereas PGST did not differ significantly from TAU (Cohen *d*, 0.30; 95% CI, −0.29 to 0.89; *P* = .32). Treatment retention was greater in the IGST arm than in the PGST (1 year: 0.82 vs 0.72; 2 years: 0.74 vs. 0.62) and TAU (1 year: 0.82 vs 0.73; 2 years: 0.74 vs 0.64) arms, and there was no significant difference between the TAU and PGST arms (1 year: 0.73 vs 0.72; 2 years: 0.64 vs 0.62).

**Conclusions and Relevance:**

In this randomized clinical trial, IGST was more effective and had greater treatment retention compared with TAU and PGST. These findings suggest that IGST is the preferred ST format, with high retention and continuation of improvement in BPD severity after the completion of treatment.

**Trial Registration:**

trialregister.nl Identifier: NTR2392

## Key Points

**Question:**

Is group schema therapy for borderline personality disorder (BPD) more effective than optimal treatment as usual, and is predominantly group schema therapy or combined individual and group schema therapy more effective?

**Findings:**

In this randomized clinical trial, which included 495 adult participants with BPD in 5 countries, combined individual and group schema therapy was significantly more effective than optimal treatment as usual and predominantly group schema therapy in reducing BPD severity.

**Meaning:**

The findings add to the evidence for the effectiveness of schema therapy for BPD and indicate that the combination of individual and group schema therapy is the more effective schema therapy format.

## Teaser

This randomized clinical trial examines whether schema therapy is more effective than optimal treatment as usual for borderline personality disorder and whether it is more effectively delivered in a predominantly group or combined individual and group format.

## Introduction

Meta-analytic reviews have found specialized therapies for borderline personality disorder (BPD) to be associated with greater reductions in overall BPD severity than treatment as usual (TAU).^1,2^ One specialized therapy is schema therapy (ST). Early trials of ST demonstrated effectiveness when delivered in an individual format.^3,4^ A large effect was found for group ST delivered as an adjunct to TAU.^5^ However, this group ST was delivered by its developers, and therefore, the generalizability of this finding to other therapists and other settings was unclear. Also unknown was the extent to which group ST would be effective as a stand-alone treatment and the relative merits of combining individual and group ST.

An international workgroup, in considering these issues, speculated that the group-only format might encourage patients to bring all issues to the group, thus optimizing group therapeutic processes. Conversely, an argument was made that combining individual with group ST would better meet patients’ needs for attention and attachment and better facilitate the processing of severe childhood adverse experiences, a hallmark of ST.^6,7^ The workgroup decided to test these competing positions through an international randomized clinical trial (RCT) with 3 arms: predominantly group ST (PGST), combined individual and group ST (IGST), and TAU.^8^ We decided to use optimal TAU as a comparator—that is, the optimal treatment that was available at the treatment site according to the usual local practice (excluding ST).

This RCT examined the effectiveness of ST in 2 formats compared with optimal TAU and also compared the effectiveness of the 2 ST formats. We investigated effectiveness for the primary outcome measure (BPD severity). Secondary outcomes included treatment retention, specific BPD and general psychiatric symptoms, quality of life, schemas and schema modes, and psychosocial functioning. We report the results of the planned analyses^8^: first, both group ST formats were combined and compared with TAU, and then the 3 arms were compared with each other. The first hypothesis was that the 2 ST formats combined would be superior to TAU in reducing BPD severity. The second hypothesis was that the 3 treatment types would differ in effectiveness. Moreover, we explored differences in treatment retention and other secondary outcomes.

## Methods

### Study Design

This international multicenter RCT had 3 arms: optimal TAU, PGST, and IGST. Participants were randomized 1:1 in cohorts of 16 to 18 participants per site either to PGST or TAU or to IGST or TAU (NTR2392) (trial protocol in Supplement 1). The order of these cohort types was randomly assigned across sites. There were 15 sites: 2 in Australia, 3 in Germany, 1 in Greece, 7 in the Netherlands, and 2 in the UK (details are provided in eAppendix 1 in Supplement 2). Because some sites could not finance participation or recruit a second cohort, other sites compensated by including extra cohorts, deviating from the plan^8^ (eAppendix 2 in Supplement 2). The number of cohorts per site varied from 1 to 5. Thirty cohorts were recruited. The mean rank order of cohort type (PGST vs IGST) was equal (eAppendix 1 in Supplement 2). The planned minimum number of participants was 448 from 28 cohorts based on a power analysis (power 90%; medium effect size, α = 0.05) and taking attrition into account.^8^ Ethical approval was obtained at each participating site. Participants provided written informed consent. This study followed the Consolidated Standards of Reporting Trials (CONSORT) reporting guideline.

### Patients

**Figure 1.**
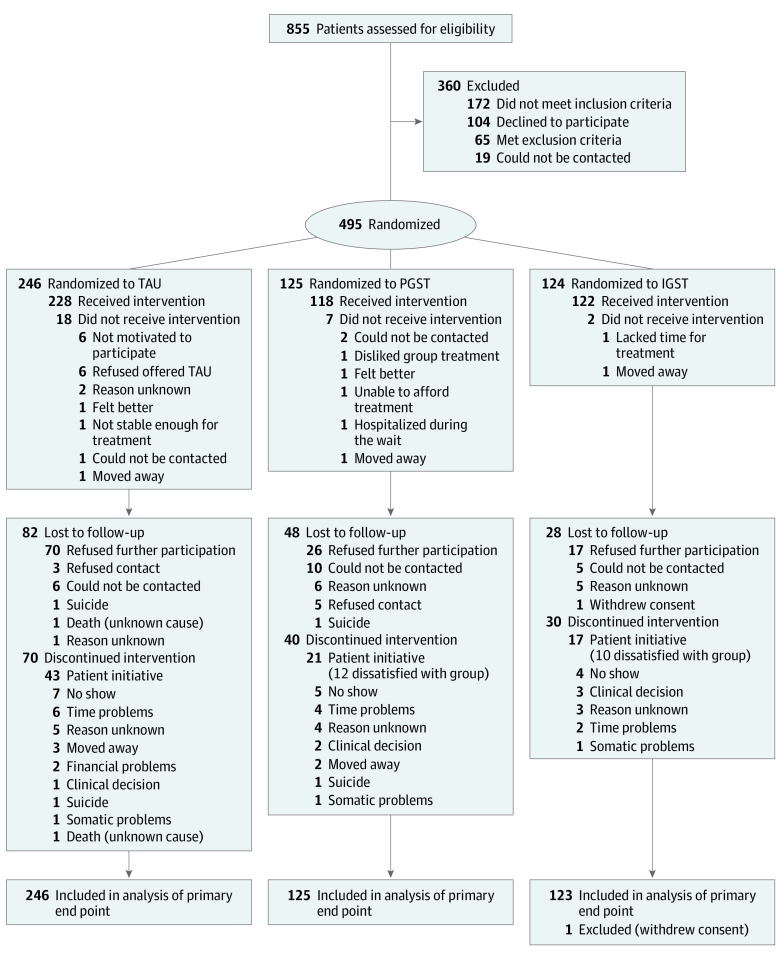
CONSORT Diagram IGST indicates combined individual and group schema therapy; PGST, predominantly group schema therapy; and TAU, (optimal) treatment as usual.

Participants were recruited between June 29, 2010, and May 18, 2016. Figure 1 shows the CONSORT flow diagram; 495 patients were randomly assigned, 1 withdrew consent, and 494 were analyzed. Inclusion criteria were a primary diagnosis of BPD, age of 18 to 65 years, a Borderline Personality Disorder Severity Index IV (BPDSI-IV) score greater than 20 (range, 0-90; a score >20 denotes clear BPD; 15 is the cutoff for BPD, and <15 is used as recovery criterion), and the willingness and ability to participate in 2 years of treatment. Exclusion criteria were an inability to speak, read, and/or understand the study site’s language, an IQ less than 80, a psychotic disorder (except reactive episodes; BPD criterion 9 per the *DSM-5*), bipolar disorder 1, dissociative identity disorder, untreated attention-deficit/hyperactivity disorder, addiction needing clinical detoxification (inclusion after detoxification was allowed), full or subthreshold narcissistic or antisocial PD, a serious and/or unstable medical illness, and having received ST for more than 3 months during the previous 3 years. Ethnicity was self-reported. Diagnoses were made with the Structured Clinical Interview for *DSM-IV* Axis I and II Disorders. Sites were eligible if they offered treatment for BPD, had therapists willing to be trained in group ST, agreed to adhere to the study protocol, and could organize and finance the study.

### Randomization and Masking

When a cohort of 16 to 18 participants was accrued, participants of that cohort were randomly assigned by a central independent research assistant using computerized randomization in blocks of 2 based on the order of completion of baseline assessments. The type of ST (PGST or IGST) per cohort was randomized across sites.^8^ Assessors were blinded to the condition. The study blind was removed after the last data became available. Given the unequal sample sizes in the 3 arms, blinding during analysis was not feasible.

### Procedures

#### Treatments and Therapists

Group ST followed a protocol.^9,10^ In PGST, 2 group sessions per week were provided in year 1, with a maximum of 12 individual sessions available on patient request. In year 2, group ST was provided once per week in months 13 to 18, once per 2 weeks in months 19 to 21, and once monthly in months 22 to 24, with a maximum of 5 individual ST sessions available on request.

For IGST, 2 sessions (1 individual^7,10^ and 1 group) per week were delivered in year 1. In the first 6 months of year 2 (months 13 to 18), frequencies of both individual and group ST were biweekly. In months 19 to 21, group ST was provided biweekly and individual ST was provided monthly. In months 22 to 24, both were offered monthly (details are shown in eTable 1 in Supplement 2).

Group ST was closed and provided by 2 therapists. For IGST, most patients had an individual therapist other than their group therapists. All ST therapists were licensed mental health professionals trained in individual ST for BPD. Group ST therapists were additionally trained by I.S. and J.F.^9^ Initially, therapists in both ST formats received online supervision by I.S. weekly; after 6 months, supervision was reduced to biweekly or monthly depending on the therapists’ experience.

Treatment as usual was the optimal psychological treatment available at the site. The most frequently offered was dialectical behavior therapy, with an intensity matched to that of ST. An overview of the types of TAU offered is shown in eTable 2 in Supplement 2.

#### Treatment Integrity and Assessments

A scale was designed to assess whether ST discriminated from TAU (eAppendix 3 in Supplement 2). Independent evaluators rated the quality of individual and group ST.^11^ There were 6 face-to-face assessments: at baseline and at 6, 12, 18, 24, and 36 months.

### Outcomes

The primary outcome was the slope over time of BPD severity during the past 3 months as assessed at baseline, 6 months, 12 months, 18 months, 24 months, and 36 months with the BPDSI-IV total score, a reliable and valid index of BPD severity.^12^ Secondary outcomes included 9 BPDSI subscales (representing the BPD criteria); suicidality and the number of suicide attempts during the past 3 months, assessed with the BPDSI-IV; the BPD Checklist^13^ (assessing the self-reported burden of BPD manifestations); Global Assessment of Functioning (*DSM-III*) and Social and Occupational Functioning Assessment Scale^14^ ratings, based on semistructured interviews^8^; the Brief Symptom Inventory^15^ (general psychopathology symptoms); the Work and Social Adjustment Scale^16^ (work and social functioning); the World Health Organization Quality of Life assessment^17^; a happiness item^8^; the Young Schema Questionnaire–short form^18^; the Schema Mode Inventory^19^ (providing 2 scores, 1 for dysfunctional and 1 for functional schema modes); and having employment or being a student. eAppendix 4 in Supplement 2 provides details on the instruments used. We assessed treatment retention because previous studies found a low rate of dropout from ST.^3,5,20^

### Statistical Analysis

Data were analyzed from June 4, 2019, to December 29, 2021. Given skewed distributions, the BPDSI total and subscale scores were analyzed with generalized linear mixed model (GLMM) gamma regression with a log link, with random effects of treatment for site and of treatment and time for the cohort nested under the site if estimation allowed (eAppendix 5 in Supplement 2). For the repeated part, an autoregressive moving average (1,1) model was used. The fixed part contained time, treatment, and their interaction, with the time × treatment interaction (the difference in slopes) testing the differential effectiveness hypothesis. Because gamma regression cannot be conducted with 0, a small value (0.01) was added to scores. Sensitivity analyses were performed to test the robustness of this choice. Effect sizes were expressed as *r* = √(*t*^2^/[*t*^2^ + *df*]) for fixed effects, and Cohen *d* was the conventional effect size, with the numerator based on estimated means from GLMM gamma regression and the denominator as baseline SD in transformed scale (based on GLMM gamma regression with a log link, without random parts, and with an intercept for the fixed part) (eAppendix 6 in Supplement 2). Treatment retention was analyzed using GLMM survival analysis of treatment dropout per quarter (every 3 months) during 2 years with the site as a random intercept. The number of suicide attempts during the previous 3 months was analyzed using generalized estimating equations with a Tweedie distribution (because of many 0 counts) with a log link and first-order autoregression for the repeated part. Depending on the distribution, secondary outcomes not based on the BPDSI were analyzed with appropriate LMM or GLMM. The time model was either linear or piecewise (if an inflection point was observed in all at 1 year). The choice of a piecewise model was supported by superior fit, tested with χ^2^ tests. For all piecewise models, the time × treatment interaction was not significant and did not lead to improved fit; thus, it was deleted, implicating that treatment effects were assessed by the difference in slopes in years 2 and 3. In accordance with the preset plan,^8^ first, both ST formats were jointly compared with TAU; next, the 3 arms were compared. Binomial tests (with a probability of 0.05) were used to assess the likelihood of the number of significant tests of secondary outcomes per comparison.^21^ Analyses were conducted in SPSS, version 25 (IBM), as intention-to-treat after the last data became available. Significance was set at 2-tailed *P* = .05. Different sample sizes made blind analysis impossible.

## Results

A total of 495 patients participated in the study (mean [SD] age, 33.6 [9.4] years; range, 18.6-61.7 years; 426 [86.2%] female). Of these, 246 (49.7%) received TAU, 125 (25.2%) received PGST, and 124 (25.0%) received IGST (1 of whom later withdrew consent). Table 1 shows baseline descriptive statistics for the participants.

**Table 1.** Descriptive Statistics at Baseline and Treatment Integrity Test Results

Characteristic	Patients^a^		
	TAU arm (n = 246)	PGST arm (n = 125)	IGST arm (n = 123)
Age, mean (SD), y	33.89 (9.55)	33.20 (9.33)	33.46 (9.17)
Sex			
Female	212 (86.2)	109 (87.2)	105 (85.4)
Male	33 (13.4)	16 (12.8)	17 (13.8)
Other or unknown	1 (0.4)	0 (0)	1 (0.8)
Relationship status			
Partner	99 (40.2)	41 (32.8)	41 (33.3)
No partner	147 (59.8)	84 (67.2)	82 (66.7)
Educational level, mean (SD)^b^	3.2 (1.6)	3.4 (1.6)	3.4 (1.7)
Ethnicity			
Same as dominant group at study site	230 (93.5)	118 (94.4)	106 (86.2)
Different from dominant group at study site	16 (6.5)	7 (5.6)	17 (13.8)
Employment status^c^			
Employed	79 (32.2)	34 (27.2)	34 (27.9)
Homemaker	16 (6.5)	6 (4.8)	7 (5.7)
Student	26 (10.6)	13 (10.4)	8 (6.6)
Disability or illness benefit	95 (38.8)	59 (47.2)	49 (40.2)
Unemployed	29 (11.8)	13 (10.4)	24 (19.7)
GAF score, mean (SD)	49.70 (8.69)	50.09 (8.10)	48.36 (8.32)
BPD severity			
BPD criteria (SCID-II), mean (SD), No.	6.83 (1.33)	7.05 (1.38)	6.81 (1.30)
BPDSI total score, mean (SD)	31.63 (8.60)	30.95 (8.75)	30.44 (8.74)
Comorbid disorders			
*DSM-IV* Axis I diagnoses (SCID-I), mean (SD), No.	3.31 (2.15)	3.57 (2.13)	3.76 (2.23)
*DSM-IV* Axis II diagnoses (SCID-II), mean (SD), No.^d^	1.74 (1.04)	1.87 (1.01)	1.76 (1.02)
PD			
Avoidant	77 (31.3)	37 (29.6)	47 (38.2)
Dependent	20 (8.1)	10 (8.0)	14 (11.4)
Obsessive-compulsive	38 (15.4)	19 (15.2)	18 (14.6)
Paranoid	48 (19.5)	21 (16.8)	27 (22.0)
Schizotypal	3 (1.2)	1 (0.8)	0 (0)
Schizoid	1 (0.4)	3 (2.4)	1 (0.8)
Histrionic	0 (0)	1 (0)	0 (0)
Conduct disorder youth	52 (21.1)	33 (26.4)	26 (21.1)
Antisocial A^e^	2 (0.8)	1 (0.8)	4 (3.3)
TIS rating, mean (SE)^f^			
Nonspecific subscale	50.69 (0.93)^g^	53.76 (1.19)	54.85 (1.16)
Specific subscale	39.21 (0.86)^g^	48.18 (1.13)	50.68 (1.09)
Non-ST subscale	4.74 (0.11)	4.47 (0.15)	4.77 (0.15)

Abbreviations: BPD, borderline personality disorder; BPDSI, Borderline Personality Disorder Severity Index; GAF, Global Assessment of Functioning; IGST, combination of individual and group schema therapy; PD, personality disorder; PGST, predominantly group schema therapy; SCID-I, Structured Clinical Interview for *DSM-IV* Axis I Disorders; SCID-II, Structured Clinical Interview for *DSM-IV* Axis II Disorders; ST, schema therapy; TAU, (optimal) treatment as usual; TIS, therapy integrity scale.

^a^ Data are presented as the number (percentage) of patients unless otherwise indicated.

^b^ Based on the International Standard Classification of Education, 2011 version, with a range of 0 to 8.

^c^ Two patients had missing responses (1 from the TAU group and 1 from the IGST group).

^d^ Included BPD.

^e^ Full antisocial PD was an exclusion criterion, as was narcissistic PD.

^f^ Means and SEs were from mixed regression of the TIS ratings at 6, 12, 18, and 24 months.

^g^ The TAU group had significantly lower TIS scores than did the ST groups (TAU vs PGST: *d*, 0.34; TAU vs IGST: *d*, 0.90; *P* < .001); scores from the PGST and IGST groups did not differ significantly from each other. Analyzed with linear mixed models with treatment and time in the fixed part, random intercept and slope for time for cohort within site, and autoregressive moving average (1,1) covariance for the repeated part.

### Assessment of Treatment Integrity

The Treatment Integrity Scale discriminated ST from TAU (Table 1). The quality of individual and group ST was rated between good and very good by independent raters^11^ (eAppendix 3 in Supplement 2).

### Primary Outcomes

Forty-two recordings of BPDSI interviews were randomly selected; these were rated by an independent assessor, and the BPDSI total scores were then compared with those reported by the original interviewer. Interrater agreement was excellent (intraclass correlation coefficient, 0.99).

For reduction of the total BPDSI-IV score, IGST and PGST combined were superior to TAU, with a medium to large effect size (Cohen *d*, 0.73; 95% CI, 0.29-1.18; *P* = .001) (Table 2 and Table 3). The difference was significant at 1.5 years (mean [SE] difference, 2.38 [0.98]; 95% CI, 0.27-4.49; *P* = .03).

**Table 2.** Estimated Means, 95% CIs, and Effect Sizes for the Primary and Secondary Outcomes

Outcome, time, y	Effect size					
	TAU		PGST		IGST	
	Estimated mean (95% CI)^a^	Cohen *d*^b^	Estimated mean (95% CI)^a^	Cohen *d*^b^	Estimated mean (95% CI)^a^	Cohen *d*^b^
**Primary outcome**						
BPDSI total score						
Baseline	30.69 (28.45-33.11)	NA	30.14 (27.44-33.10)	NA	30.51 (27.80-33.49)	NA
0.5	27.46 (25.51-29.56)	0.40	26.59 (24.33-29.06)	0.45	25.90 (23.72-28.28)	0.59
1.0	24.57 (22.77-26.51)	0.80	23.47 (21.42-25.71)	0.90	21.98 (20.10-24.05)	1.18
1.5	21.98 (20.22-23.89)	1.20	20.71 (18.71-22.92)	1.35	18.66 (16.90-20.60)	1.77
2.0	19.67 (17.87-21.64)	1.60	18.27 (16.24-20.56)	1.80	15.84 (14.13-17.75)	2.36
3.0	15.74 (13.84-17.90)	2.41	14.23 (12.12-16.70)	2.70	11.41 (9.78-13.31)	3.55
**Secondary outcomes**						
BPD checklist						
Baseline	67.04 (61.47-73.12)	NA	68.95 (61.01-77.92)	NA	64.43 (57.01-72.81)	NA
0.5	60.31 (55.50-65.53)	0.24	60.93 (54.23-68.45)	0.28	56.08 (49.93-63.00)	0.31
1.0	54.23 (49.76-59.16)	0.47	53.84 (47.80-60.66)	0.55	48.82 (43.38-54.94)	0.62
1.5	48.81 (44.31-53.77)	0.71	47.58 (41.77-54.20)	0.83	42.50 (37.39-48.30)	0.93
2.0	43.91 (39.25-49.17)	0.95	42.05 (36.26-48.77)	1.11	36.99 (32.02-42.74)	1.24
3.0	35.54 (30.54-41.36)	1.42	32.84 (26.98-39.98)	1.66	28.03 (23.19-33.89)	1.86
GAF						
Baseline	51.06 (48.42-53.71)	NA	50.87 (47.72-54.03)	NA	48.78 (45.65-51.91)	NA
0.5	53.04 (50.46-55.63)	0.23	53.22 (50.17-56.27)	0.28	51.70 (48.68-54.73)	0.35
1.0	55.03 (52.42-57.63)	0.47	55.57 (52.50-58.63)	0.55	54.62 (51.59-57.65)	0.69
1.5	57.01 (54.32-59.70)	0.70	57.91 (54.72-61.10)	0.83	57.54 (54.40-60.68)	1.04
2.0	58.99 (56.15-61.83)	0.94	60.26 (56.84-63.68)	1.11	60.46 (57.11-63.81)	1.38
3.0	62.95 (59.62-66.28)	1.41	64.95 (60.82-69.09)	1.66	66.30 (62.29-70.32)	2.07
SOFAS						
Baseline	51.86 (49.36-54.36)	NA	51.30 (48.27-54.34)	NA	49.44 (46.43-52.45)	NA
0.5	53.78 (51.32-56.23)	0.21	53.83 (50.88-55.77)	0.28	52.18 (49.26-55.10)	0.30
1.0	55.69 (53.19-58.19)	0.42	56.35 (53.35-59.35)	0.56	54.92 (51.97-57.88)	0.61
1.5	57.61 (54.97-60.25)	0.64	58.87 (55.69-62.06)	0.84	57.67 (54.54-60.80)	0.91
2.0	59.52 (56.66-62.39)	0.85	61.40 (57.91-64.88)	1.12	60.41 (57.00-63.83)	1.21
3.0	63.36 (59.84-66.88)	1.27	66.44 (62.09-70.80)	1.68	65.90 (61.66-70.14)	1.82
WSAS						
Baseline	23.23 (21.75-24.82)	NA	23.55 (21.52-25.78)	NA	23.54 (21.51-25.76)	NA
0.5	20.53 (19.24-21.90)	0.33	20.81 (19.04-22.75)	0.33	20.80 (19.04-22.72)	0.33
1.0	18.14 (16.77-19.61)	0.66	18.39 (16.66-20.30)	0.66	18.38 (16.67-20.26)	0.66
1.5	17.43 (16.03-18.95)	0.77	16.82 (15.18-18.65)	0.90	16.92 (15.28-18.73)	0.88
2.0	16.75 (15.17-18.49)	0.87	15.39 (13.64-17.37)	1.14	15.57 (13.83-17.53)	1.10
3.0	15.46 (13.39-17.85)	1.09	12.88 (10.76-15.42)	1.61	13.19 (11.10-15.68)	1.55
WHO QOL						
Baseline	70.62 (65.42-75.83)	NA	69.46 (62.07-76.86)	NA	71.05 (63.66-78.45)	NA
0.5	75.29 (70.10-80.49)	0.26	74.14 (66.75-81.52)	0.26	75.73 (68.34-83.11)	0.26
1.0	81.45 (76.05-86.85)	0.51	80.60 (73.07-88.13)	0.51	83.82 (76.30-91.33)	0.51
1.5	81.45 (76.05-86.85)	0.60	80.60 (73.07-88.13)	0.61	83.82 (76.30-91.33)	0.70
2.0	82.93 (77.34-88.53)	0.68	82.39 (74.65-90.12)	0.71	87.24 (79.54-94.94)	0.89
3.0	85.90 (79.59-92.21)	0.84	85.97 (77.37-94.56)	0.91	94.08 (85.60-102.56)	1.27
BSI						
Baseline	1.86 (1.73-2.01)	NA	1.99 (1.81-2.18)		1.85 (1.69-2.03)	NA
0.5	1.66 (1.54-1.79)	0.30	1.77 (1.61-1.94)	0.30	1.65 (1.50-1.80)	0.30
1.0	1.48 (1.36-1.60)	0.60	1.58 (1.43-1.74)	0.60	1.46 (1.33-1.61)	0.60
1.5	1.40 (1.29-1.52)	0.74	1.45 (1.31-1.60)	0.81	1.32 (1.19-1.45)	0.87
2.0	1.33 (1.21-1.46)	0.88	1.34 (1.19-1.50)	1.03	1.19 (1.06-1.32)	1.15
3.0	1.19 (1.05-1.35)	1.16	1.13 (0.97-1.33)	1.45	0.96 (0.83-1.12)	1.69
YSQ						
Baseline	55.82 (54.03-57.61)	NA	56.38 (53.89-58.88)	NA	56.55 (54.05-59.05)	NA
0.5	51.79 (50.02-53.55)	0.40	52.34 (49.87-54.82)	0.40	52.51 (50.04-54.98)	0.40
1.0	47.75 (45.83-49.66)	0.81	48.30 (45.72-50.88)	0.81	48.47 (45.92-51.03)	0.81
1.5	46.68 (44.76-48.61)	0.92	46.49 (43.91-49.06)	0.99	46.07 (43.51-48.62)	1.05
2.0	45.62 (43.55-47.70)	1.02	44.67 (41.91-47.43)	1.17	43.66 (40.94-46.34)	1.29
3.0	43.50 (40.81-46.19)	1.23	41.03 (37.45-44.62)	1.54	38.85 (35.38-42.31)	1.77
SMI-dysfunctional						
Baseline	48.98 (47.90-50.06)	NA	49.54 (48.04-51.03)	NA	48.88 (47.39-50.38)	NA
0.5	45.79 (44.73-46.85)	0.44	46.35 (44.87-47.82)	0.44	45.69 (44.23-47.16)	0.44
1.0	42.60 (41.41-43.79)	0.88	43.16 (41.59-44.73)	0.88	42.51 (40.95-44.06)	0.88
1.5	41.73 (40.54-42.93)	0.99	41.90 (40.35-43.46)	1.05	40.90 (39.36-42.43)	1.10
2.0	40.87 (39.54-42.19)	1.11	40.65 (38.93-42.37)	1.22	39.28 (37.60-40.97)	1.32
3.0	39.13 (37.29-40.97)	1.35	38.15 (35.74-40.56)	1.56	36.06 (33.76-38.37)	1.76
SMI-functional						
Baseline	5.73 (5.52-5.93)	NA	5.70 (5.41-5.99)	NA	5.66 (5.37-5.95)	NA
0.5	6.18 (5.97-6.38)	0.35	6.15 (5.87-6.44)	0.35	6.11 (5.83-6.40)	0.35
1.0	6.63 (6.49-6.86)	0.71	6.60 (6.30-6.90)	0.71	6.57 (6.27-6.86)	0.71
1.5	6.73 (6.50-6.97)	0.79	6.75 (6.45-7.06)	0.83	6.82 (6.52-7.12)	0.91
2.0	6.84 (6.58-7.09)	0.87	6.90 (6.57-7.24)	0.94	7.08 (6.75-7.41)	1.11
3.0	7.05 (6.70-7.39)	1.03	7.21 (6.76-7.65)	1.18	7.59 (7.16-8.02)	1.51
Happiness						
Baseline	3.08 (2.92-3.25)	NA	2.90 (2.68-3.13)	NA	3.16 (2.94-3.39)	NA
0.5	3.42 (3.26-3.58)	0.30	3.24 (3.02-3.46)	0.30	3.50 (3.38-3.72)	0.30
1.0	3.76 (3.60-3.93)	0.59	3.57 (3.34-3.81)	0.59	3.83 (3.60-4.06)	0.59
1.5	3.81 (3.64-3.98)	0.64	3.75 (3.52-3.98)	0.75	3.94 (3.72-4.16)	0.69
2.0	3.87 (3.69-4.05)	0.69	3.93 (3.81-4.29)	0.91	4.05 (3.81-4.29)	0.78
3.0	3.98 (3.74-4.23)	0.79	4.28 (3.94-4.62)	1.22	4.26 (3.94-4.58)	0.97
Working/studying proportion						
Baseline	0.42 (0.31-0.54)	NA	0.38 (0.26-0.51)	NA	0.34 (0.23-0.48)	NA
0.5	0.42 (0.31-0.53)	−0.01	0.40 (0.28-0.53)	0.09	0.37 (0.25-0.50)	0.09
1.0	0.41 (0.31-0.53)	−0.02	0.42 (0.30-0.56)	0.18	0.39 (0.27-0.52)	0.18
1.5	0.41 (0.30-0.53)	−0.03	0.44 (0.31-0.58)	0.28	0.41 (0.29-0.55)	0.28
2.0	0.41 (0.29-0.54)	−0.04	0.47 (0.32-0.61)	0.37	0.43 (0.30-0.58)	0.37
3.0	0.41 (0.28-0.55)	−0.05	0.51 (0.34-0.68)	0.55	0.48 (0.32-0.64)	0.55

Abbreviations: BPD, borderline personality disorder; BPDSI, Borderline Personality Disorder Severity Index; BSI, Brief Symptom Inventory; GAF, Global Assessment of Functioning; IGST, combination of individual and group schema therapy; NA, not applicable; PGST, predominantly group schema therapy; SMI, Schema Mode Inventory; SOFAS, Social and Occupational Functioning Assessment Scale; TAU, (optimal) treatment as usual; WSAS, Work and Social Adjustment Scale; WHO QOL, World Health Organization Quality of Life assessment; YSQ, Young Schema Questionnaire–short form.

^a^ Estimated means and 95% CIs are in original scale.

^b^ Cohen *d* values are based on the parameters of the generalized linear mixed model analyses (change over time), with the square root of the baseline variance of a model with no random parts and only a fixed intercept as the denominator (ie, SD baseline in transformed scale).

**Table 3.** Comparison of Time Effects for First and Secondary Outcomes Between the ST Groups Combined vs the TAU Group, the PGST Group vs the TAU Group, the IGST Group vs the TAU Group, and the IGST Group vs the PGST Group^a^

Outcome, comparison	Time effects, slope over 3 y				
	*t*	*df*	*P*	Cohen *d* (95% CI)^b^	*r*
**Primary outcome**					
Borderline Personality Disorder Severity Index total score					
Main time effect	14.11	31	<.001	2.76 (2.36 to 3.16)	0.93
Time × treatment					
ST vs TAU^c^	3.24	1028	<.001	0.73 (0.29 to 1.18)	0.10
PGST vs TAU	0.99	924	.32	0.30 (−0.29 to 0.89)	0.03
IGST vs TAU^c^	3.95	916	<.001	1.14 (0.57 to 1.71)	0.13
IGST vs PGST^c^	2.21	491	.03	0.84 (0.09 to 1.59)	0.10
**Secondary outcomes**					
Borderline personality disorder checklist^d^					
Main time effect	11.33	31	<.001	1.59 (1.30 to 1.85)	0.90
Time × treatment					
ST vs TAU^c^	2.03	910	.04	0.33 (0.00 to 0.67)	0.07
PGST vs TAU	1.05	763	.30	0.24 (–0.69 to 0.21)	0.04
IGST vs TAU^c^	2.03	777	.04	0.44 (0.44 to 0.87)	0.07
IGST vs PGST	0.71	377	.48	0.20 (–0.36 to 0.77)	0.04
Global Assessment of Functioning					
Main time effect	12.19	35	<.001	1.64 (1.37 to 1.92)	0.90
Time × treatment					
ST vs TAU^c^	2.68	745	.008	0.49 (0.13 to 0.84)	0.10
PGST vs TAU	1.09	704	.28	0.26 (–0.21 to 0.73)	0.04
IGST vs TAU^c^	2.90	708	.004	0.67 (0.22 to 1.12)	0.11
IGST vs PGST	1.38	314	.17	0.41 (–0.17 to 0.99)	0.08
Social and Occupational Functioning Assessment Scale					
Main time effect	10.36	31	<.001	1.51 (1.21 to 1.81)	0.88
Time × treatment					
ST vs TAU^c^	2.92	636	.004	0.48 (0.16 to 0.81)	0.12
PGST vs TAU	1.81	639	.07	0.40 (–0.04 to 0.84)	0.07
IGST vs TAU^c^	2.56	624	.01	0.55 (0.13 to 0.97)	0.10
IGST vs PGST	0.52	389	.61	0.15 (–0.70 to 0.41)	0.03
Work and Social Adjustment Scale^e^					
Main time effect					
Whole period	7.52	165	<.001	1.33 (0.81 to 1.86)	0.51
Years 2 and 3	−3.26	1120	.001		0.10
Time × treatment					
ST vs TAU^c^	2.88	990	.004	0.49 (0.16 to 0.82)	0.09
PGST vs TAU^c^	2.32	1008	.02	0.52 (0.08 to 0.97)	0.07
IGST vs TAU^c^	2.16	1003	.03	0.46 (0.04 to 0.88)	0.07
IGST vs PGST	−0.23	755	.82	−0.06 (–0.61 to 0.48)	0.01
World Health Organization Quality of Life assessment^f^					
Main time effect					
Whole period	10.30	163	<.001	0.97 (0.68 to 1.26)	0.63
Years 2 and 3	−5.04	1119	<.001		0.15
Time × treatment					
ST vs TAU^c^	2.58	804	.01	0.26 (0.06 to 0.46)	0.09
PGST vs TAU	0.50	784	.62	0.07 (–0.20 to 0.33)	0.02
IGST vs TAU^c^	3.37	787	<.001	0.43 (0.18 to 0.67)	0.12
IGST vs PGST^c^	2.17	514	.03	0.36 (0.03 to 0.68)	0.10
Brief Symptom Inventory^e^					
Main time effect					
Whole period	8.43	218	<.001	1.36 (0.94 to 1.78)	0.50
Years 2 and 3	−2.61	1140	.009		0.08
Time × treatment					
ST vs TAU^c^	2.81	888	.005	0.42 (0.13 to 0.72)	0.09
PGST vs TAU	1.48	848	.14	0.29 (–0.10 to 0.68)	0.05
IGST vs TAU^c^	2.88	854	.004	0.54 (0.17 to 0.90)	0.10
IGST vs PGST	1.01	559	.31	0.24 (–0.23 to 0.71)	0.04
Young Schema Questionnaire–short form^f^					
Main time effect					
Whole period	13.97	224	<.001	1.44 (1.11 to 1.79)	0.68
Years 2 and 3	−7.03	1014	<.001		0.22
Time × treatment					
ST vs TAU^c^	3.34	939	<.001	0.43 (0.18 to 0.68)	0.11
PGST vs TAU	1.82	809	.07	0.30 (–0.02 to 0.63)	0.06
IGST vs TAU^c^	3.42	844	<.001	0.54 (0.23 to 0.85)	0.12
IGST vs PGST	1.18	405	.24	0.24 (–0.16 to 0.63)	0.06
Schema Mode Inventory–dysfunctional^f^					
Main time effect					
Whole period	14.89	234	<.001	1.51 (1.17 to 1.86)	0.70
Years 2 and 3	−7.90	992	<.001		0.24
Time × treatment					
ST vs TAU^c^	2.44	997	.02	0.31 (0.06 to 1.86)	0.08
PGST vs TAU	1.24	842	.21	0.21 (–0.12 to 0.54)	0.04
IGST vs TAU^c^	2.54	894	.01	0.41 (0.09 to 0.72)	0.08
IGST vs PGST	0.97	441	.33	0.20 (–0.20 to 0.59)	0.05
Schema Mode Inventory–functional^f^					
Main time effect					
Whole period	12.09	217	<.001	1.19 (0.75 to 1.42)	0.63
Years 2 and 3	−6.66	1047	<.001		0.20
Time × treatment					
ST vs TAU^c^	2.58	805	.01	0.32 (0.08 to 0.57)	0.09
PGST vs TAU	0.89	743	.38	0.15 (–0.18 to 0.47)	0.03
IGST vs TAU^c^	3.07	765	.002	0.48 (0.17 to 0.78)	0.11
IGST vs PGST	1.67	445	.10	0.33 (–0.06 to 0.72)	0.08
Happiness^f^					
Main time effect					
Whole period	10.03	538	<.001	0.94 (0.59 to 1.29)	0.40
Years 2 and 3	−5.40	1384	<.001		0.14
Time × treatment					
ST vs TAU^c^	2.31	661	.02	0.29 (0.04 to 0.54)	0.09
PGST vs TAU^c^	2.61	572	.009	0.42 (0.10 to 0.74)	0.11
IGST vs TAU	1.16	597	.25	0.18 (–0.12 to 0.48)	0.05
IGST vs PGST	1.30	264	.20	−0.18 (–0.48 to 0.12)	0.08
Working/studying proportion^g^					
Main time effect	1.40	33	.17	0.25 (–0.11 to 0.61)	0.24
Time × treatment					
ST vs TAU^c^	2.22	590	.03	0.59 (0.07 to 1.11)	0.09
PGST vs TAU	1.78	497	.08	0.60 (–0.06 to 1.27)	0.08
IGST vs TAU	1.84	477	.07	0.60 (–0.04 to 1.25)	0.08
IGST vs PGST	0.00	233	>.99	0.00 (–0.82 to 0.81)	0.00

Abbreviations: GLMM, generalized linear mixed model; IGST, combination of individual and group schema therapy; PGST, predominantly group schema therapy; ST, schema therapy arms combined; TAU, (optimal) treatment as usual.

^a^ All analyses are based on GLMM with an autoregressive moving average (1,1) covariance structure for the repeated part and a random effect of time for a cohort within a site. Effect sizes *d* are based on the parameters of the GLMM analyses (change over time), with the square root of the baseline variance of a model with no random parts and only a fixed intercept as the denominator. Effect sizes *r* are defined as *r* = √(*t*^2^/[*t*^2^ + *df*]). These represent the effect size associated with the effect tests in the fixed part of the GLMM. The *t* values and Cohen *d* values are positive when there is a positive effect (ie, a reduction over time, a larger reduction in ST than in TAU). The main time effect is the mean across ST and TAU.

^b^ Cohen *d* values are based on the parameters of the GLMM analyses (change over time), with the square root of the baseline variance of a model with no random parts and only a fixed intercept as the denominator (ie, SD baseline in transformed scale). The 95% CIs of the Cohen *d* are based on the 95% CIs of the slope differences from the GLMM.

^c^ The interaction of treatment by time was significant.

^d^ Analyzed with GLMM gamma regression with a log link. Estimated means are −46.99 points lower than on the original scale. Scores were transformed by subtracting 46.99 to bring the minimum to just greater than 0 to enable gamma regression.

^e^ Piecewise gamma regression with a general slope for time and an additional slope for the second and third year was used. Deleting the general time × treatment interaction from the model increased model fit; thus, the final model was time (general), time (years 2 and 3), treatment, and treatment × time (years 2 and 3). Interactions for the second time slope are shown (second and third year). To enable GLMM gamma regression with a log link, 0.1 was added to raw scores.

^f^ Piecewise regression with a general slope for time and an additional slope for the second and third year was used. Deleting the nonsignificant general time × treatment interaction from the model did not reduce model fit; thus, the final model was treatment, time (general), time (years 2 and 3), and time (years 2 and 3) × treatment. Interactions for the second time slope are shown (second and third year).

^g^ Analyzed with GLMM logistic regression. Estimated means are the proportion of working to studying.

**Figure 2.**
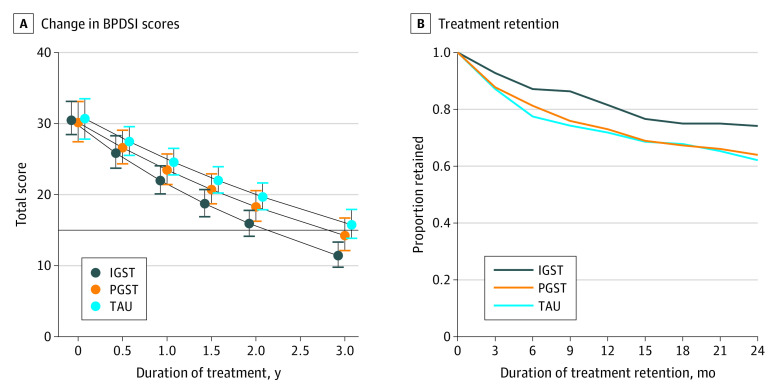
Borderline Personality Disorder Severity Index (BPDSI) Scores and Duration of Treatment Retention A, Estimated marginal means and 95% CIs from the generalized linear mixed model (GLMM) analysis of the total BPDSI scores for the 3 treatment arms. The horizontal line indicates the clinical BPDSI cutoff score of 15, and whiskers indicate 95% CIs. B, Treatment retention by study arm quarterly for 2 years, from the GLMM survival analysis. IGST indicates combined individual and group schema therapy; PGST, predominantly group schema therapy; and TAU, (optimal) treatment as usual.

When the 3 arms were mutually compared for treatment effectiveness, IGST was superior to PGST and TAU (IGST vs PGST: Cohen *d*, 0.84; 95% CI, 0.09-1.59; *P* = .03; IGST vs TAU: Cohen *d*, 1.14; 95% CI, 0.57-1.71; *P* < .001). The effectiveness of PGST did not differ significantly from that of TAU (Cohen *d*, 0.30; 95% CI, −0.29 to 0.89; *P* = .32) (Table 2, Table 3, and Figure 2A). The difference in effectiveness of IGST compared with TAU became significant at 1 year of treatment (difference, 2.59; 95% CI, 0.05-5.13; *P* = .048), and compared with PGST, at 2.5 years (difference, 2.68; 95% CI, 0.12-5.25; *P* = .04).

During the 3-year period, differences in slope between IGST and the other treatments attained large effect sizes (IGST vs TAU: Cohen *d*, 1.14; 95% CI, 0.57-1.71; *P* < .001; IGST vs PGST: Cohen *d*, 0.84; 95% CI, 0.09-1.59; *P* = .03) (Table 2 and Table 3). The TAU effect sizes at 1 year were 0.80 for the primary outcome and 0.56 for secondary non-BPDSI outcomes.

Results of a loc-f sensitivity analysis are given in eTable 3 in Supplement 2. Results of sensitivity analyses for different offset values are shown in eTable 4 in Supplement 2. Descriptive statistics of raw BPDSI scores and results in a transformed scale are shown in eTable 5 in Supplement 2.

### Secondary Outcomes

#### Treatment Retention

Figure 2B shows treatment retention during years 1 and 2. The GLMM survival analysis on treatment dropout per yearly quarter showed no significant difference between ST and TAU but superiority of IGST compared with PGST (1 year: 0.82 vs 0.72; 2 years: 0.74 vs. 0.62) and TAU (1 year: 0.82 vs 0.73; 2 years: 0.74 vs 0.64). Treatment as usual and PGST did not differ significantly for treatment retention (1 year: 0.73 vs 0.72; 2 years: 0.64 vs 0.62) (eTable 5 in Supplement 2).

#### Severity per BPD Trait

Predominantly group ST and IGST combined were superior to TAU on 6 of the 9 BPDSI subscales (binomial test, *P* < .001) (eTable 6 in Supplement 2); IGST was superior to TAU on 7 subscales (binomial test, *P* < .001) and to PGST on 2 subscales (binomial test, *P* = .07). PGST was not significantly different from TAU on any of the subscales (binomial test, *P* > .99). The effects of PGST were between those of TAU and IGST for all subscales apart from emptiness, for which the effect of PGST was smaller than that of TAU.

#### Suicidality and Suicide Attempts

For scores on suicidality items of the BPDSI (para)suicide scale, PGST and IGST combined were superior to TAU (eTable 6 in Supplement 2). Only IGST was superior to TAU; IGST and PGST did not differ significantly in reducing suicidality scores.

Overall, the number of suicide attempts during the previous 3 months reduced with time, with a nonsignificant difference between ST and TAU but with IGST being significantly superior to TAU in reducing suicide attempts (eTable 6 in Supplement 2).

#### Secondary Outcomes Not Based on BPDSI

For all 11 secondary outcomes, PGST and IGST combined were superior to TAU (binomial test, *P* < .001); IGST was superior to TAU for 9 of the 11 outcomes (binomial test, *P* < .001) (Table 2 and Table 3). PGST was superior to TAU for only 2 outcomes (the Work and Social Adjustment Scale and happiness; binomial test, *P* = .10). IGST was superior to PGST for 1 of 11 secondary outcomes (binomial test, *P* = .43), the World Health Organization Quality of Life Assessment. The overall ST effect was largely attributable to IGST, with PGST outcomes between those of TAU and IGST.

During the study period, 3 patients died of suicide (1 from each treatment arm). The patient from the PGST arm applied successfully to a euthanasia clinic during the first 3 months (quarter 1) of treatment. The patient from the IGST arm died by suicide approximately 8 months after discontinuing treatment and study during quarter 2 (months 4 to 6). A TAU recipient died by suicide during quarter 3 (months 7 to 9). In the TAU arm, an additional patient died in quarter 2 of an unknown cause; suicide was suspected but not confirmed.

#### Deterioration

Deterioration was defined as a BPDSI score increase of 11.70 or greater compared with baseline.^3^ Of the available BPDSI assessments, more deteriorations were observed in the TAU arm (16 of 745 assessments [2.1%]) than in the PGST arm (5 of 398 [1.25%]) and the IGST arm (4 of 452 [0.88%]).

## Discussion

This international RCT compared 2 ST formats (PGST and IGST) with TAU and with each other in the treatment of BPD across 15 sites in 5 countries. Although the PGST and IGST combined appeared to be superior to TAU in reducing BPD severity (the primary outcome) and for most secondary outcomes, most effects were attributable to IGST; there were also fewer suicide attempts and treatment dropouts in this arm. IGST was superior to TAU in reducing BPD severity and for 7 BPDSI subscales. Moreover, IGST was superior to PGST in reducing BPD severity and for 2 BPDSI subscales. In summary, the results support IGST as an effective, acceptable (as indexed by treatment retention), and safe treatment for BPD, whereas the results were more equivocal for PGST.

The 3-year effects were generally large in all arms. Treatment as usual was effective given the within-condition effect sizes. Compared with the pooled pretreatment-posttreatment effect size of 0.5 for dialectical behavior therapy,^22^ usually found at 1 year, the TAU effect sizes at 1 year in our study were similar (0.80 for the primary outcome and a mean effect size of 0.56 for secondary non-BPDSI outcomes). This indicates that optimal TAU was offered.

With regard to the reason that IGST was superior to PGST, IGST had relatively large effects on schemas, schema modes, and affective instability, variables that are assumed to underlie change processes.^23,24^ Core emotional needs such as safe attachment and positive attention are often not adequately met during childhood in patients with BPD in both individual and group relationships. The combined ST format aimed to meet needs in both contexts, whereas PGST provided less individual attention. Moreover, addressing severe problems and childhood trauma might be easier for therapists in individual treatment than in group treatment. This was reported by participating therapists in a qualitative ancillary study.^25^ Consistent with these hypotheses, patients in another ancillary qualitative study^26^ stated that individual ST was important to discuss sensitive topics on a deeper, more personal level. Most patients (75%) emphasized the importance of individual ST in conjunction with group ST, and 70% of the participants in receiving PGST expressed the need for more individual sessions. Also indicative of the importance of individual ST, PGST participants in the current study generally used the maximum number of individual sessions. How well individual ST compares with IGST and which format is optimal for whom are topics for future studies.

When comparing, to our knowledge, the first RCT on individual ST for BPD^3^ with the present study, there appeared to be equivalent effect sizes. There were more sessions in the former study (approximately double), but the dropout rate was lower. Group treatment might be associated with increased dropout for a variety of reasons, including distrust, frightening group processes, or lack of an individualized agenda. Some patients in the ancillary qualitative study^26^ described hostility from group members as particularly difficult. Individual sessions might mitigate the problem of aversive group dynamics.

The present study has some implications about the sufficient dosage of ST sessions. A finding of note was the continuation of symptom improvement during years 2 and 3. In the ST arms, session frequency was reduced to only once a month using a tapered schedule, and in year 3, no further treatment was offered. Although patients often resist this tapering, feeling not ready and expressing fear of relapse,^26^ in our study, they tended to do well. At the 3-year follow-up, some participants expressed how helpful it was to discover that they were able to apply what they learned after therapy was discontinued. They commented that this helped to increase their self-confidence and changed their self-view as a patient with chronic mental illness who is dependent on long-term mental health care.

Despite the generally positive results, there were 3 suicides and 1 death of unknown cause during the study, with 1 suicide in each treatment arm. One of the deaths was related to a preset plan to apply for euthanasia that the person did not share with the treating team. Another participant died by suicide after dropping out of treatment. Nevertheless, in general, suicidality was reduced during treatment, as were suicide attempts, with IGST being superior to TAU in reducing suicidality and suicide attempts.

### Limitations

This study had several limitations. First, the worldwide financial crisis of 2007-2008, followed by an economic crisis (in most countries), interfered with study implementation, leading some sites that had planned to participate to withdraw owing to cutbacks. This had to be compensated by recruiting additional sites and by sites including additional cohorts. Although the mean rank order of the ST formats was equal, the distribution deviated from the original plan. Second, as with all RCTs, participants dropped out of the study, which can affect the validity of the results. Third, rating of TAU recordings to assess differentiation between ST and TAU was impossible. However, patients’ self-reports supported that ST was different from TAU. Moreover, assessments of adherence to ST by independent raters supported that ST was generally delivered at a good level of quality. Fourth, group ST was generally delivered by novice group ST therapists who were specifically trained for the study without having had the opportunity to learn the intervention before the study commenced. Some aspects of the group intervention (eg, early conflict management) required a high skill level. For some of the individual ST therapists, it was the first time they applied ST. Thus, ST effects might increase and the number of dropouts may be reduced with experience, repeated training, and supervision. Fifth, TAU was unstandardized. Future RCTs should compare ST with other specialized psychotherapies, controlling for intensity of treatment.

## Conclusions

In this RCT, IGST was more effective in reducing the severity of BPD compared with TAU and PGST. However, PGST was not more effective than TAU. The international multicenter design, the size of the study population, and the execution of the study in regular mental health services that were treating BPD support the generalizability of the results. However, this study did not compare ST with another specialized psychotherapy, and no conclusions in this respect can be made. The cost-effectiveness of ST is a subject for further study. Future research should focus on the direct comparison of individual and combined ST formats; the testing of less extreme combinations of group and individual ST; direct comparisons of ST with other evidence-based treatments, including the study of factors associated with better treatment allocation; and the testing of ST in countries in other regions of the world.

## Notes

Supplement 1.
Trial Protocol

Supplement 2. **eAppendix 1.** Overview of Sites and Cohorts per Site **eAppendix 2.** Deviations From the Original Plan **eTable 1.** Overview of Type and Frequency of Sessions Over 2 Years for PGST and IGST **eTable 2.** Overview of Initial TAU per Site **eAppendix 3.** Assessment of Treatment Integrity **eAppendix 4.** Clinical Outcome Measures **eAppendix 5.** Overview of Random Effects for Secondary Outcomes **eAppendix 6.** Estimation Procedures for the Effect Size Cohen *d* and its 95% CI **eTable 3.** Results of Loc-f Sensitivity Analysis **eTable 4.** Sensitivity Analysis of the Zero-Offset Value for the Gamma Regression of the BPDSI Total Score **eTable 5.** Descriptive Statistics of Observed BPDSI Total Scores, GLMM Results in Transformed Scale, Pairwise Contrasts at 3 years in Original Scale, Treatment Retention per 3 Months by Treatment Arm **eTable 6.** BPDSI-Based Secondary Outcomes: BSPDI Subscales, Suicidality, Suicide Attempts

Supplement 3.
Data Sharing Statement

## References

[1] Storebø OJ, Stoffers-Winterling JM, Völlm BA, . Psychological therapies for people with borderline personality disorder. Cochrane Database Syst Rev. 2020;5:CD012955. 32368793 10.1002/14651858.CD012955.pub2 PMC7199382

[2] Oud M, Arntz A, Hermens ML, Verhoef R, Kendall T. Specialized psychotherapies for adults with borderline personality disorder: a systematic review and meta-analysis. Aust N Z J Psychiatry. 2018;52(10):949-961. doi:10.1177/0004867418791257 30091375 PMC6151959

[3] Giesen-Bloo J, van Dyck R, Spinhoven P, . Outpatient psychotherapy for borderline personality disorder: randomized trial of schema-focused therapy vs transference-focused psychotherapy. Arch Gen Psychiatry. 2006;63(6):649-658. doi:10.1001/archpsyc.63.6.649 16754838

[4] Nadort M, Arntz A, Smit JH, . Implementation of outpatient schema therapy for borderline personality disorder with versus without crisis support by the therapist outside office hours: a randomized trial. Behav Res Ther. 2009;47(11):961-973. doi:10.1016/j.brat.2009.07.013 19698939

[5] Farrell JM, Shaw IA, Webber MA. A schema-focused approach to group psychotherapy for outpatients with borderline personality disorder: a randomized controlled trial. J Behav Ther Exp Psychiatry. 2009;40(2):317-328. doi:10.1016/j.jbtep.2009.01.002 19176222

[6] Weertman A, Arntz A. Effectiveness of treatment of childhood memories in cognitive therapy for personality disorders: a controlled study contrasting methods focusing on the present and methods focusing on childhood memories. Behav Res Ther. 2007;45(9):2133-2143. doi:10.1016/j.brat.2007.02.013 17462588

[7] Arntz A, van Genderen H. Schema Therapy for Borderline Personality Disorder. Wiley Blackwell; 2009.

[8] Wetzelaer P, Farrell J, Evers SMMA, . Design of an international multicentre RCT on group schema therapy for borderline personality disorder. BMC Psychiatry. 2014;14:319. doi:10.1186/s12888-014-0319-3 25407009 PMC4240856

[9] Farrell JM, Shaw IA. Group Schema Therapy for Borderline Personality Disorder: A Step-by-Step Treatment Manual With Patient Workbook. John Wiley & Sons; 2012. doi:10.1002/9781119943167

[10] Farrell JM, Reiss N, Shaw IA. The Schema Therapy Clinician’s Guide: A Complete Resource for Building and Delivering Individual, Group and Integrated Schema Mode Treatment Programs. John Wiley & Sons; 2014. doi:10.1002/9781118510018

[11] Bastick E, Bot S, Verhagen SJW, . The development and psychometric evaluation of the Group Schema Therapy Rating Scale–Revised. Behav Cogn Psychother. 2018;46(5):601-618. doi:10.1017/S1352465817000741 29370876

[12] Arntz A, van den Hoorn M, Cornelis J, Verheul R, van den Bosch WM, de Bie AJHT. Reliability and validity of the borderline personality disorder severity index. J Pers Disord. 2003;17(1):45-59. doi:10.1521/pedi.17.1.45.24053 12659546

[13] Bloo J, Arntz A, Schouten E. The Borderline Personality Disorder Checklist: psychometric evaluation and factorial structure in clinical and nonclinical samples. Ann Psychol. 2017; 20(2): 281-336. doi:10.18290/rpsych.2017.20.2-3en

[14] Goldman HH, Skodol AE, Lave TR. Revising Axis V for *DSM-IV*: a review of measures of social functioning. Am J Psychiatry. 1992;149(9):1148-1156. doi:10.1176/ajp.149.9.1148 1386964

[15] Derogatis LR, Melisaratos N. The Brief Symptom Inventory: an introductory report. Psychol Med. 1983;13(3):595-605. doi:10.1017/S0033291700048017 6622612

[16] Mundt JC, Marks IM, Shear MK, Greist JH. The Work and Social Adjustment Scale: a simple measure of impairment in functioning. Br J Psychiatry. 2002;180:461-464. doi:10.1192/bjp.180.5.461 11983645

[17] The WHOQOL Group. Development of the World Health Organization WHOQOL-BREF quality of life assessment. Psychol Med. 1998;28(3):551-558. doi:10.1017/S0033291798006667 9626712

[18] Young JE, Brown G. Young Schema Questionnaire Short Form. Cognitive Therapy Center; 1998.

[19] Lobbestael J, van Vreeswijk M, Spinhoven P, Schouten E, Arntz A. Reliability and validity of the short Schema Mode Inventory (SMI). Behav Cogn Psychother. 2010;38(4):437-458. doi:10.1017/S1352465810000226 20487590

[20] Bamelis LL, Evers SM, Spinhoven P, Arntz A. Results of a multicenter randomized controlled trial of the clinical effectiveness of schema therapy for personality disorders. Am J Psychiatry. 2014;171(3):305-322. doi:10.1176/appi.ajp.2013.12040518 24322378

[21] Wilkinson B. A statistical consideration in psychological research. Psychol Bull. 1951;48(3):156-158. doi:10.1037/h0059111 14834286

[22] Kliem S, Kröger C, Kosfelder J. Dialectical behavior therapy for borderline personality disorder: a meta-analysis using mixed-effects modeling. J Consult Clin Psychol. 2010;78(6):936-951. doi:10.1037/a0021015 21114345

[23] Yakın D, Grasman R, Arntz A. Schema modes as a common mechanism of change in personality pathology and functioning: results from a randomized controlled trial. Behav Res Ther. 2020;126:103553. doi:10.1016/j.brat.2020.103553 32018065

[24] Von Klipstein L, Borsboom D, Arntz A. The exploratory value of cross-sectional partial correlation networks: predicting relationships between change trajectories in borderline personality disorder. PloS One. 2021;16(7):e0254496. doi:10.1371/journal.pone.0254496 34329316 PMC8323921

[25] Martius D. The experience of schema therapists who provided the treatment: what we learnt. Paper presented at: 9th World Congress of Behavioural and Cognitive Therapies; July 17-20, 2019; Berlin. Accessed January 28, 2022. https://wcbct2019.org/Downloads/Congress-Programme.pdf/

[26] Tan YM, Lee CW, Averbeck LE, . Schema therapy for borderline personality disorder: a qualitative study of patients’ perceptions. PloS One. 2018;13(11):e0206039. doi:10.1371/journal.pone.0206039 30462650 PMC6248917

