# Supplementary material for: Effectiveness of Predominantly Group Schema Therapy and Combined Individual and Group Schema Therapy for Borderline Personality Disorder: A Randomized Clinical Trial
Source: JAMA Psychiatry. 2022 Mar 2;79(4):287–99. doi: 10.1001/jamapsychiatry.2022.0010 (PMC8892362; doi:10.1001/jamapsychiatry.2022.0010)
Supplement: Supplement 1. — Trial Protocol [file jamapsychiatry-e220010-s001.pdf]

Algemene gegevens / General Information

Programma / Programme	:	
Subsidieronde / Subsidy round	:	**Ronde 12 Grant application E&K**
Projecttitel / Project title	:	**Group Schema Therapy for Borderline Personality Disorder**
Projecttaal / Project language	:	**Engels / English**
Geplande startdatum / Planned start date	:	**01-01-2012**
Geplande duur / Planned duration	:	**48 maanden / months**
Datum indienen / Date of application	:	**08-02-2011**
Projecttype / Project type	:	**Toegepast onderzoek / Applied research**
Vervolg eerder ZonMw-project / Continuation previously funded project ZonMw	:	**Nee / No**

Aanvrager / Applicant

Prof. dr. A. Arntz
T: 043-3881228 | *F:* 043-3884155 | *E:* Arnoud.Arntz@maastrichtuniversity.nl

Universiteit Maastricht
Onderzoekscentrum Experimentele Psychopathologie
Postbus 616
6200 MD MAASTRICHT

Projectleden / Project members

Prof. dr. A. Arntz (Projectleider en penvoerder)
Functie / Position: Hoogleraar | *Opleiding / Education:*
Studierichting / Subject:
T: 043-3881228 | *F:* | *E:* Arnoud.Arntz@Maastrichtuniversity.nl

Universiteit Maastricht
Onderzoekscentrum Experimentele Psychopathologie
Postbus 616
6200 MD MAASTRICHT

Dr. G.J.P. van Breukelen (Mede aanvrager)
Functie / Position: UHD | *Opleiding / Education:*
Studierichting / Subject:
T: 043-3884001 | *F:* | *E:*

Universiteit Maastricht
Department of Methodology and Statistics
P Debyeplein 1
6229 HA MAASTRICHT

Dr. M.J.J.M. Candel (Mede aanvrager)
Functie / Position: UD | *Opleiding / Education:*
Studierichting / Subject:
T: 043-3882273 | *F:* | *E:*

Universiteit Maastricht
Department of Methodology and Statistics
P Debyeplein 1
6229 HA MAASTRICHT

Dr. S. Evers (Mede aanvrager)
Functie / Position: UHD | *Opleiding / Education:*
Studierichting / Subject:
T: 043-3881727 | *F:* | *E:*

Universiteit Maastricht
School for Public Health and Primary Care (Caphri)
Postbus 616
6200 MD MAASTRICHT

Dr. J. Farrell (Mede aanvrager)
Functie / Position: assistant professor | *Opleiding / Education:*
Studierichting / Subject:
T: 00-1-317-941-4331 | *F:* | *E:*

Larue Carter Hospital
2601 Cold spring Road
INDIANAPOLIS

Drs. H. van Genderen (Mede aanvrager)
Functie / Position: Klinisch Psycholoog | *Opleiding / Education:*
Studierichting / Subject:
T: 043-3299611 | *F:* | *E:*

RIAGG
Maastricht
Parallelweg 45-47
6221 BD MAASTRICHT

Prof. dr. B. Jansma (Bestuurlijk verantwoordelijke)
Functie / Position: decaan | *Opleiding / Education:*
Studierichting / Subject:
T: 043-3881937 | *F:* | *E:*

Maastricht University
Faculty of Psychology and Neuroscience
Clinical Psychological Science
Postbus 616
6200 MD MAASTRICHT

Drs. M. van Vreeswijk (Mede aanvrager)
Functie / Position: Directeur | *Opleiding / Education:*
Studierichting / Subject:
T: 06-52036234 | *F:* | *E:*

G-Kracht, Psychomedisch centrum
Rietveld 45-47
2611 LG DELFT

Projectgegevens / Project information

Aandachtsgebieden / Focus

Psychische stoornissen
- Suïcide/suïcidaliteit

Samenvatting / Summary

SAMENVATTING

Doel: Borderline Persoonlijkheidsstoornis (BPS) is een ernstige psychische stoornis met hoog suicide-risico (10%). Groeps-Schematherapie (ST) is een nieuwe en effectieve behandeling met een potentieel grote kosten-effectiviteit. Er zijn twee formats van groeps-ST effectief en veilig bevonden: groep (format A) en de combinatie van groep en individuele ST (format B).
De doelen van het onderzoek zijn:
1. de (kosten-)effectiviteit vast te stellen van groeps-ST voor BPS.
2. de visies van patienten en therapeuten op de behandelingen vast te stellen.

Primaire onderzoeksvraag: is groeps-ST superieur aan de gebruikelijke behandeling (TAU)?
Secondaire onderzoeksvraag: is er een verschil tussen de twee groeps-ST formats?

Studie opzet: multicenter RCT met 3 armen: 2 vormen van groeps-ST, en TAU. 6 centra (elk N=32) participeren, in elk wordt ST-A, ST-B en TAU gegeven.

Studiepopulatie: 192 BPS patienten geworven in 6 participerende GGZ instellingen.

Interventie: 2 jaar groeps-ST (format A), gecombineerde groep-individuele-ST (format B), of TAU.

Uitkomstmaten: primaire uitkomst is verandering in BPS-ernst vastgesteld met de BPDSI, een gestructureerd interview naar de ernst van BPS; secondaire maten: andere BPS-maten, suicidaliteit, sociaal functioneren, algemene psychiatrische symptomen, kwaliteit van leven. Metingen op baseline, elke 6 maanden gedurende 2 jaar, en op 3 jaar follow-up.

Sample Size berekeningen/data analyse: De primaire onderzoeksvraag wordt getoetst met multilevel analyse, door groeps-ST te vergelijken met TAU (contrast: formats A+B vs TAU; power=.9, alpha =.05). De secundaire vraag door groeps-ST formats A en B met elkaar te vergelijken (contrast A vs B; power=.8, alpha=.05).

Economische evaluatie: gecombineerde kosten-effectiviteits/kosten-utiliteits analyse vanuit maatschappelijk perspectief, met dezelfde contrasten als voor effectiviteit.

Kwalitatieve studie: diepte-interviews en focusgroepen om de visies van patienten en therapeuten vast te stellen, w.o. het geprefereerde groeps-ST format.

Tijdpad:
2010-11: training therapeuten, patientenwerving, start behandeling, supervisie, metingen
2012: laatste inclusie, laatste behandeling start, supervisie, metingen, data checks
2013: behandelingen, supervisie, metingen, treatment integrity checks, 1e 3-jr follow-up, data checks, kwalitatieve studie
2014: metingen, einde laatste behandeling, 3-jr follow-up, data cleaning, analyses, publicaties
2015: laatste 3-jr follow-up, analyses, rapportage, publicaties.

SUMMARY

Objectives: Borderline Persoanlity Disorder (BPD) is a severe mental disorder with high suicidality (10%). Group Schema Therapy (ST) is a new effective treatment with potential high cost-effectiveness. Two formats of group-ST were found to be effective and safe: mainly group (format A) and the combination of group and individual ST (format B).

Aims:
1. to assess the (cost-)effectiveness of group-ST for BPD.
2. to assess patients' and therapists' views on the treatments.

Primary research question: is group-ST superior to Treatment as Usual (TAU)?
Secondary research question: is there a difference between two group-ST formats?

Design: multicenter RCT with 3 arms: 2 forms of group-ST, and TAU. 6 centers (each N=32) participate, in each ST-A, ST-B and TAU is given.

Population: 192 BPD patients recruited at 6 participating mental health care centers.

Intervention: 2 years of group-ST (format A), combined group-individual-ST (format B), or TAU.

Outcome measures: primary outcome is change in BPD-severity assessed with the BPDSI, a structured interview to assess BPD severity; secondary outcomes: other BPD-measures, suicidality, social functioning, general psychiatric symptoms, quality of life. Assessements at baseline, every 6 months during 2 years, and at 3 year follow-up.

Statistical analysis: The primary research question will be tested with multilevel analysis by comparing group-ST to TAU (contrast: formats A+B vs TAU; power=.9, alpha=.05). The secundary question by comparing group-ST formats A and B to eachother (contrast A vs B; power=.8, alpha=.05).

Economic evaluation: combined cost-effectiveness/cost-utility analysis from a societal perspective, using the same contrasts as for effectiveness.

Qualitative study: in-depths interviews and focus groups to assess patients' and therapists' opinions, including about the prefered group-ST format.

Time schedule:
2010-2011: therapists training, patient recruitment, first treatments start, supervision, assessments
2012: recruitment complete, last treatment start, supervision, assessments, reliability checks, data check
2013: treatments, supervision, assessments, treatment integrity, first 3 yr follow-up, data check, qualitative study
2014: assessments, last treatments, 3 yr follow-up, data check, analysis of 2 year treatment, publications
2015: last 3 yr follow-up, data analyses, final report, publications.

Trefwoorden / Keywords
Borderline Personality Disorder; Schema Therapy; RCT; Psychotherapy; cost-effectiveness; qualitative research

Samenwerking / Collaboration

Samenwerking tussen onderzoek en praktijk / Cooperation between research and practice:
Ja / Yes

Organisaties

G-Kracht, Psychomedisch centrum
Rietveld 45-47
2611 LG DELFT

GGZ Oost Brabant
Postbus 51
5700 AB HELMOND

Mondriaan Zorggroep
Instelling voor GGZ
Postbus 4436
6401 CX HEERLEN

RIAGG
Parallelweg 45-47
6221 BD MAASTRICHT

Symfora Groep
Rembrandthof
Laan van de Heelmeesters 2
1211 MS HILVERSUM

Vincent van Gogh Instituut
Stationsweg 46
5803 AC VENRAY

Inhoud / Content

Probleemstelling / Problem definition

Borderline Personality Disorder (BPD) is a highly prevalent (1.5-2% of general population) and severe mental disorder with high societal costs[1], high health care costs, and high suicidality (ranging from para-suicide (self-injury & risky behaviors) to suicide attempts). ± 10% of untreated/improperly treated BPD-patients dies by suicide, 1000 times more than the general population[2,3]. BPD-patients have a low quality of life and lead an unfulfilled life, functioning at lower levels than what would expected given their capacities. There is a risk of intergenerational transmission due to problematic childcare by parents with BPD.

Effective psychological treatments for BPD have been developed[33]. There is no medication that can cure BPD[34], but psychological treatments can bring about recovery. Schema Therapy (ST) is a particularly effective treatment with large effects[4-7], not only reducing all aspects of BPD pathology, but also improving quality of life, and normalizing emotional brain responses in the amygdala, hippocampus and ACC[35]. Cost-effectiveness of ST has been assessed in comparison to a specialized psychodynamic treatment of BPD, TFP[6]. A problem is that ST has not yet been compared to usual care for BPD in a cost-effectiveness analysis, seriously limiting the knowledge on which dissemination and implementation decisions should be based.

Despite Dutch guidelines clearly indicating specialized psychotherapy as first choice treatment[36], BPD patients often get other treatments that are not very effective. A Dutch study found that only 25% of BPD patients received psychotherapy[37]. This implies that 75% of BPD patients is given pharmacotherapy and/or supportive contacts that have little empirical evidence as effective full treatments. Focusing on ST for BPD, one explanation for its limited availibility is that standard ST is individual treatment with a length of 1.5 to 4 years, severely limiting the availibility of ST to consumers and increasing the direct treatment delivery costs.

A recent development is group-ST, which appears to bring about a faster reduction of BPD severity than individual ST. After one year, group-ST studies found large effects on BPD severity reduction (d's > 2), larger than those found with individual ST after 1 year (d~1.25). In sum, group-ST is shorter (2 years), can be given to more patients, and has a faster effect than individual ST. Group-ST is not the simple application of ST-techniques in a group. Rather, the model uses specific group processes such as coherence and a group-developmental model to catalyze the change processes that are used by ST. In sum, group-ST might help to make ST more available for BPD patients for lower costs. But, two models of group-ST have been developed and found to be both promising in terms of effects. The first is a model mainly using groups (format "A")[4], the other a model combining individual and group-ST (format "B")[11]. To date it is unclear which of these 2 formats is to be preferred in

terms of effectiveness, costs, and preferences of the most important stakeholders, patients and therapists.

The present study therefore has as primary aim to assess the effectiveness and the cost-effectiveness of group-ST in general (i.e., across the two formats). To get a valid indication of the cost-effectiveness, group-ST will be compared to usual care (Treatment as Usual, TAU), following the gold standard of cost-effectiveness research[17]. As there is no single TAU given to BPD patients in the Netherlands, we will follow the actual usual practice in the control condition, and let participating centers decide in their usual manner about the treatment offered to the BPD patient. The resulting amalgam of treatments will represent usual care in the centers and provide the intended contrast both in effects and in costs.

As a secondary aim, we want to directly compare the two group-ST formats as to effectiveness and cost-effectiveness.

We also aim to give a say to the most important stakeholders of BPD treatment: patients and therapists. Patients are the consumers and should therefore been given a say in further developments. Therapists often experience treatment of BPD patients as complex and frustrating. It is important that they inform us about the format they prefer, and about further needs and wishes. For this aim, qualitative methods are used.

Relevantie / Relevance

BPD is a highly prevalent (1.5-2% of general population) and severe mental disorder with high societal costs[1], high health care costs, and high suicidality (ranging from para-suicide (self-injury & risky behaviors) and suicide attempts to actual suicide. ± 10% of untreated/improperly treated BPD-patients dies by suicide, 1000 times more than the general population[2,3]. Effective treatments exist, but only few lead to full recovery. ST has proven effectiveness, reduces (para)suicidality profoundly[5] and can lead to full recovery[4-7]. Its recovery rates are higher than TAU and TFP, an alternative specialized treatment[4,5]. ST is more cost-effective than TFP[6], and an implementation study has proven that ST can be successfully implemented in regular health care without loss of effectiveness[7]. In contrast to many other treatments, ST leads to full recovery, and not to a partial remission of a subset of the symptoms of BPD. With respect to (para)suicidality, ST does not focus on (para)suicidal symptoms in isolation, but as part of the whole problem. With ST, BPD patients not only quickly reduce their (para)suicidal behavior, but also improve in all aspects of BPD, improve their functioning and quality of life, and reduce their costs of illness[4-7].

But, individual ST is a lengthy (2-4 years) and costly treatment. Given its length, need for specialist therapists, and individual application, availability for consumers is limited.

Group-ST is a new approach proven to be effective and safe[4,11], but its cost-effectiveness has not been assessed. Group-ST is probably cheaper than individual-ST. It uses less therapist time per patient, and group-processes catalyze healing processes, leading to faster recovery than individual treatment[4,11]. For group-ST, direct care-delivery costs per patient are 30%-60% of those of individual-ST. This makes group-ST an attractive treatment for mental health care institutes, insurance companies, and society as a whole. Moreover, the two small-scale group-ST studies done so far found very strong changes already in one year (d>2[4,11]), larger than individual ST obtains in one year (d≈1.25 [5,7]).

Although efficiency in terms of direct delivery costs is considerably enhanced with group-ST, it is unclear how group-ST compares to usual care for BPD in effectiveness and cost-effectiveness. It is also unclear which format is optimal: solely group-ST, or group-ST combined with individual ST. Lastly, it is unclear what the views and the preferred format of the major stakeholders, patients and therapists, are.

If group-ST proves to be (cost)effective, application leads to improvement of health care as more patients can be treated for less costs with hihger effects in a relatively short time.

No overlap with other ZONMW projects.

Kennisoverdracht, implementatie, bestendiging / Knowledge transfer, Implementation Consolidation

There are five major groups that will be informed about our experiences and the outcomes of the study:
1. Scientists;
2. Clinicians;
3. BPD-patients;
4. Policy makers;
5. General lay public.

1. Scientists.
The aim of our scientific dissemination will be to inform investigators in the fields of psychiatry, clinical psychology, psychotherapy research and mental health HTA about the effectiveness and cost-effectiveness of the two group-ST formats as treatment for BPD. Results will be presented at national and international conferences, and published in scientific journals. Relevant results of the qualitative analyses will similarly be presented at conferences and published in scientific journals.

2. Clinicians.
We have two major aims with our dissemination plans for clinicians. First, to inform clinicians about the effectiveness of group-ST, the preferred format, and experiences of therapists and patients elicited thru the qualitative substudy. Second, to train interested clinicians in group-ST. For the last aim, we already started with the following activities: (1) publication of a treatment manual, in international collaboration with the developers of group-ST, Joan Farrell and Ida Shaw. A draft of the protocol book has now been completed, a final version will be published by Wiley this year, and a Dutch translation will be

published soon after that, probably by "Uitgeverij Nieuwezijds". (2) To support further dissemination and implementation, video examples of group-ST sessions and the application of specific techniques will be developed and made available for clinicians. (3) A Dutch training course in group-ST has been developed and is currently tried out by Hannie van Genderen (co-applicant) and coworkers. Dutch clinicians can subscribe to the course, and when completed, use it for their registration (renewal). The course will also be used in the psychotherapy and clinical psychology post-master training programs in the Netherlands, and be offered as in-company training. Senior therapists of the trial will become trainers in these courses.

3. Patients.
The primary dissemination aim with respect to patients is to informs them about the effectiveness of group-ST (in its optimal format), and about what they can expect in terms of the kinds of processes they will be confronted with during this treatment. Patients will be informed about the effectiveness of the two forms of group-ST by presentations on patient conferences, by informing patient organizations, by information on patient internet platforms, and by integrating the information in patient brochures. Information about the processes that take place during group-ST and the kind of experiences that are to be expected with this treatment will be mainly based on the qualitative study of this project, and will be used for a patient information brochure that can be used when the treatment is implemented in daily practice.

4. Policy makers.
The major aim of dissemination activities towards policy makers (notably, managers of mental health institutes and insurance companies) is to inform them about the cost-effectiveness and the empirical foundation of group-ST. Especially if group-ST is found to be more cost-effective (e.g., dominant) than TAU, it is important that these groups are informed so that the treatment gets widely available for patients. Summaries of our findings will be sent to these groups, and published in relevant outlets.

5. General lay public.
The aim of dissemination activities towards the general public is to further inform them that people with BPD can recover from this disorder, and - if group-ST is found to be cost-effective - that this new treatment is a offering a good and scientifically grounded method for recovery from BPD. The publication of our first ST treatment study already draw a lot of attention in the general press, and we believe that with further findings we can reinforce new views on BPD and its treatability. Press releases, interviews on television, radio, and publications in newspapers, popular magazines, and other media will be used for this purpose.

Doelstelling / Objective

This study aims to assess the (cost-)effectiveness of group-ST for BPD by comparing it to treatment as usual (TAU).

As a secondary aim, the study investigates which of the two formats of group-ST is the most (cost-)effective, and which form is preferred by the major stakeholders, patients and therapists.

To assess effectiveness, cost-effectiveness and cost-utility of group-ST, two forms of group-ST (A+B) will be jointly compared to treatment as usual (TAU).
The hypotheses that are tested are:
(1) group-ST (A+B) is more effective than TAU in terms of reduction of BPD-severity
(2) group-ST (A+B) is more cost-effective than TAU with reduction of BPD-severity as clinical outcome
(3) group-ST (A+B) has higher cost-utility than TAU

To compare the two formats of group-ST, the following hypotheses are tested:
(4) the two forms of group-ST differ in terms of reduction of BPD-severity (format A vs. B)
(5) the two forms of group-ST differ in terms of cost-effectiveness with reduction of BPD-severity as clinical outcome (A vs. B)
(6) the two forms of group-ST differ in terms cost-utility (A vs. B).

The cost-effectiveness/utility issues will be addressed from a societal viewpoint, that is not only direct healthcare costs are taken into account, but all costs associated with BPD.

In addition to the primary outcome, reduction of BPD-severity, changes in specific BPD-symptoms, notably (para)suicidality, and in general psychiatric symptoms, general and social functioning, and quality of life will be assessed.

We further aim to assess primary stakeholders' perspectives about the form of group-ST they prefer, and their experience and perceptions about the treatment to further improve it. Qualitative methods will be used to obtain patients' and therapists' opinions about these issues.

Plan van Aanpak / Strategy

CLINICAL STUDY

Previous studies by applicants.

In a multicenter RCT we compared individual ST and TFP, a psychodynamic treatment, as treatments of BPD. ST was found to be more effective and cost-effective than TFP[5-6]. Individual ST had a high within condition effect size (d=2.96) on BPD severity (assessed with BPDSI), but was rather lengthy (3 years), with 2 individual sessions a week. For this trial we developed the BPDSI, a structured interview to assess BPD severity that is suitable to assess change due to treatment, and has high reliability and validity[26-27]. In a second trial the implementation of individual ST in regular practice was investigated[7]. In that trial, the usefulness of telephone availability of the therapist for crisis outside office hours was investigated. No positive effects

of this extra service were demonstrated, which eliminated a barrier to implementation. Changes during treatment remained very strong. A next development was to develop group-ST to reduce treatment delivery costs and to speed up recovery by using specific group factors. Farrell et al. demonstrated that group-ST was more effective than TAU[4], with high effect sizes (d>2) in a shorter time period (<1 year) than in the RCTs on individual ST[4,7]. The Maastricht group developed a combined individual-group ST format, that had similar strong effects in a shorter time period than individual therapy[11]. Both studies found high acceptability and safety, and low drop-out from group-ST.
Two major questions arised: (1) how does group-ST compare to TAU in terms of effectiveness, cost-effectiveness, and opinions of the major stakeholders (patients and therapists)? (2) how do the two ST formats compare on these aspects?

Study population

192 BPD patients at 6 sites: RIAGG Maastricht, Mondriaan Zorggroep Heerlen, G-kracht Delft, GGZ Oost Brabant Helmond, Symfora Hilversum, Vincent van Gogh Institute Venray. These centers have consented to participate, 5 already started.

Inclusion criteria are:
1. Age 18-65 yr
2. Primary DSM-IV BPD diagnosis (SCID-2 interview)
3. BPDSI severity >20 (this is an empirically derived severity criterion, distinguishing BPD pathology from other PD-pathologye with high sensitivity and specificity[26,27])
4. Willingness to participate
5. Ability to participate for 3 years.

Exclusion criteria are:
1. Lifetime psychotic disorder
2. IQ<80
3. Unable to read, speak, write Dutch language
4. ADHD (but allowed when under proper control)
5. Bipolar disorder type 1
6. Dissociative Identity Disorder
7. Full or sub-threshold narcissistic or antisocial PD(SCID-2) (participants with too many of these features will endanger safety in the group for the other participants)
8. Substance dependence needing clinical detox (like heroin dependency). After succesful detoxification they can participate. Substance dependence not needing clinical detox is allowed (and is common in BPD)
9. Serious medical illness
10. Previous >3 months ST.

The following diagnostic instruments are used in the assessment of in/exclusion criteria: SCID-1 (axis-1); SCID-2 (axis-2); self-developed semi-structured interview about motivation and ability to participate in a 3 yr trial; BPDSI; semi-structured interview to detect signs of DID; followed up by a SCID-D interview if DID is suspected; WAIS to assess intelligence if low IQ is suspected; Dutch language comprehension reading test if language problems are suspected; WHO ADHD screen for adults (followed up by the KID-SCID, ADHD section if screen is positive).

Interventions

1. ST-A (Group-focused): 124 90-min group sessions plus 2-18 individual sessions (used at the patient’s discretion or for crisis) over 2 years. Two sessions a week in the first year, session frequency gradually decreases in the second year.
2. ST-B (Group and individual combined): 74 group sessions plus 62 individual sessions over 2 years. In the first year 1 group (90 min) and one individual (50 min) session per week. In the second year session frequency gradually decreases.
3. TAU: center's regular intake staff will decide what treatment is given (ST excluded) on the basis of the usual procedures. There is not one TAU for BPD in the Netherlands, and treatment is typically decided by an intake staff on the basis of clinical considerations that appear to have validity for instance for psychodynamic treatment[32]. Treatments will vary from supportive to intensive therapy, in outpatient, day treatment, to inpatient settings. This will represent current practice in the Netherlands, and is important to document how group-ST compares to TAU in the Netherlands. Type of TAU and its utilization will be carefully monitored and described. This is also important for the cost-effectiveness analysis.

Both experimental conditions have a written protocol and will be supervised (Skype & live) by Dr. Farrell. Individual ST-therapists are trained in ST for BPD. Group-ST-therapists were trained in group-ST in May 2009 and February 2010 by Dr. Farrell.
Additional medication is allowed and will be prescribed by independent psychiatrists, and controlled for in the analyses. Similar for additional crisis treatments.

Treatment integrity and quality checks will be done by independent specialists, rating a random sample of video recordings of group-ST sessions and audio-recordings of individual sessions using a revised version of the instrument used in our previous trials [5-7].

Outcome measures

Primary outcome measure is BPD severity, assessed with a semi-structured interview, the BPDSI, also used in[5,7,11].

Secondary outcomes: (1) interview-based: recovery from BPD and reliable change (BPDSI), BPDSI subscales (notably, suicidality)(the BPDSI yields dimensional scores for each of the BPD criteria), general functioning (GAF score DSM-IV), social/occupational functioning (SOFAS, DSM-IV). Interviewers will be independent and blind for condition. A random selection of interview recordings is used to assess interrater reliability. (2) selfreport-based: BPD-symptoms, quality of life (QoL; WHOQoL; EuroQoL), general psychiatric symptoms (BSI), social functioning (WSAS).

A secured internet-based program with a database facility at Maastricht University is used for questionnaires and other local data entry. Local research assistants have been trained and are supervised by the project leader and a central research assistant.

Design

Multicenter RCT. 6 centers recruit 32 BPD patients each; 8 randomly assigned to ST-A, 8 to ST-B, and 16 to TAU. Total N = 192. Assessments will take place at baseline and every 6 months during 2 years of treatment, with a follow-up at 3 years. The BPDSI is assessed twice during baseline to check for changes during baseline.

Randomization
Patients will be centrally randomized by an independent research assistent after qualification with computerized randomization of blocks of 2 patients per center (ST vs. TAU). Each center has two cohorts of 16 patients, per cohort 8 are randomized to ST, 8 to TAU. Order of format of ST (A-B or B-A) is balanced and randomized over centers, so that 3 centers start with A, 3 with B.

The flowchart in the appendix depicts the design.

Statistical analysis

Data will be analyzed with mixed (multilevel) regression for repeated measures, with dummy indicators for ST-A and ST-B and using TAU as reference category, and taking cohort and center effects into account as fixed effects (the nr of centers is too small for a random effects approach). Of primary interest are the between-group contrasts (TAU versus ST, and ST-A versus ST-B) after 2 of treatment, but intermediate measures (at 6, 12 and 18 months) increase power and allow inclusion of dropouts into the analyses. Starting with a general model for the repeated measures covariance structure and for the group by time interaction, model reduction will be aimed at by likelihood ratio testing, using the following candidate models: linear divergence between groups over the treatment period; divergence until 12 months and constancy of the between-group difference from 12 till 24 months. The analysis allows running coavriates, like medication use, to be taken into account.
Secondary outcome variables will be analyzed with the same method, but here significance will be only concluded if at least 2 measures will be significant at $p<.05$ in the same direction.

Power analysis

1. Primary research question (group-ST is superior to TAU)

The sample size calculation is based on an effect size d = .5 for the between-group outcome difference after treatment. This is a minimum effect we expect, based on the following considerations.
1. The Farrell et al. RCT demonstrated a superior effect of group-ST compared to TAU on BPD severity with a between effect size d > 2, and a within-group effect size of d>2 for group-ST and a within-group d near zero for TAU[4].
2. Shrinkage of the effect of group-ST when provided by centres that did not develop group-ST can be expected. A shrinkage in terms of within condition changes of Cohens'd > 2 to d = 1.5 is taken into consideration.
3. TAU was completely ineffective in the Farrell et al. RCT (within condition change effect size = 0)[4]. This might reflect US healthcare several years ago, but we don't expect a complete lack of effect of TAU in the present trial. Whereas the within TAU changes were virtually zero in the Farrell et al RCT[4], meta analyses and recent RCTs suggest that modern treatments are associated with within-condition changes of about d=1 [13-16].
Thus, allowing some shrinkage of the effects of group-ST from a within-group change effect size of d>2 (see also systematic review) to d=1.5, and a within-group effect size of d=1 for TAU, we expect at least a difference of d = 1.5-1 = .5 between group-ST and TAU.
The presently assumed effect size is therefore reasonable and not overoptimistic.

We expect a 20% dropout over the three years of investigation, with about 5% in year 1. After one year of treatment the effects between ST and TAU are expected to become apparent (e.g., [4]), so that the study is powered taking a 5% attrition into account. Later attrition will be buffered by the use of mixed regression that allows inclusion of all available data from dropouts into the analysis so that dropouts still contribute to the power.

Hypothesis ST > TAU: With 6 sites with each 2 cohorts (total N=192) and 5% dropout, the power is 90% at alpha=.05 (2-tailed) to detect a medium effect (d=.50, Cohen's d).

2. Secondary research question (ST-A and ST-B differ)

For this secondary research question, we accept less power (80%) than for the primary research question (90%). The test of this hypothesis involves the comparison of ST-A and ST-B, thus with half the sample, N=96. With 5% attrition at year 1, power=

80% at alpha=.05 (2-tailed) to detect a difference of d = .60. Such a difference could be expected if, for instance, one format differs from TAU with an effect size of d=.20 (small effect), the other with d=.80 (large effect). (The ST-TAU contrast would then have an effect size of d=.50, the average of .20 and .80; whereas the two ST formats would differ .80-.20=.60).

Feasibility of recruitment

BPD is a common disorder with prevalences ranging from about 1.5-2% in the general population to 10% or more in outpatients of mental health institutes. The number of patients referred to participating centers is high enough to complete recruitment in 1 to 1.5 yr in each center. Our experience is that about 50% of the patients referred to the study is included, making the numbers manageable[5-7]. Participating centers have specialized treatment facilities or treatment programmes for BPD patients, facilitating their recruitment. Five centers have already started recruitment and the first cohorts have already started treatment.

ECONOMIC EVALUATION

General Considerations

A major aim of economic evaluation is to compare additional costs and additional outcomes of new treatments to usual care (TAU)[17]. The comparison to TAU is pivotal for decision making about the implementation of new treatments. In this study we compare a new treatment, group-ST, to TAU. The economic evaluation will involve a combination of cost-effectiveness analysis (CEA) and cost-utility analysis (CUA). In a CEA effects are presented in clinical outcomes (here BPD severity decrease). The primary outcome for the CUA is QALYs, based on the EuroQol utility scores [18-19]. In the CUA, the ICER will be expressed as incremental costs per QALY. This economic evaluation will be performed from a societal perspective, which implies that all relevant costs and outcomes are taken into account. The time horizon is the same as the follow-up period of the main study, 36 months. Costs (the use of resources) are measured continuously (for details see costs measurement), outcomes for the economic evaluation study are measured at pre-test before random assignment into groups, and every 6 months during the first 2 years and 1 year follow-up during the last year. As a secondary question, we compare cost-effectiveness and cost-utility between the 2 group-ST formats using the same methodologies as for the primary research question.

Cost analysis

Total costs will be estimated using a bottom-up (or micro-costing) approach, where information on each element of service used is multiplied by an appropriate unit cost and summed to provide an overall total cost[20]. We will assess intervention, healthcare, patient, and family costs, and costs outside the health care sector. We use a cost interview especially designed for this group[6], which identifies all relevant costs aspects. To measure the actual use of resources data will be obtained using combined sources (registrations by professionals and interview). Resources used relating tot the interventions are based on the registered time all professionals spent on treatment. Intervention costs include costs of development and administration of group-ST, for example costs of training.
The valuation of healthcare costs, patient and family costs will be based on the updated Dutch manual for cost analysis in healthcare research [21]. This manual recommends using standardized cost prices. In brief, the manual recommends that prices of informal care will be based on shadow prices for unpaid work (meaning a standard cost price based on general hourly wages). Costs of transport will be calculated as the mean distance per destination multiplied by standard cost prices. Costs of medication will be calculated using prices based on Daily Defined Dosage (DDD) taken from the Dutch Pharmacotherapeutic Compass (College voor zorgverzekeringen, 2007), indicating the mean medication usage per adult a day inclusive claw back (by the government imposed discount for patients, paid by the pharmacy). Productivity costs will be calculated by means of the friction cost method, based on a mean added value of the Dutch working population. The friction costs method takes into account production losses confined to the period needed (usually 90 days) to replace a sick employee. In case of uncertainty we will use a conservative estimation (i.e. the lowest cost price). Cost prices will be expressed in 2011 euros. If necessary, existing cost-prices will be updated to 2011 using the consumer price index (CPI)[21].

Patient outcome analysis

The primary outcome measure for the CEA is decrease in BPD severity during 3-years of follow-up. For the CUA, outcomes are measured with the EuroQol (EQ-5D)[19]. This self-report questionnaire is completed at all assessments together with the cost interview. Both generic quality of life and utilities are derived from the EQ-5D. The EQ-5D is chosen because it is a widely used quality of life instrument (also internationally). The EQ-5D contains 5 dimensions of health-related quality of life: mobility, self-care, daily activities, pain/discomfort and depression/anxiety. Utility values can be calculated for these health states, using preferences elicited from a general population, the so-called social tariffs. These utilities are used to calculate Quality Adjusted Life Year (QALY). The utilities at the 6 time points are used to compute QALY scores by means of the area under the curve method.

Analysis

For the analyses we will use SPSS statistical software and Excel (for Bootstraps). A baseline analysis will be performed to examine the comparability of groups at baseline for both costs and outcomes. If necessary methods are applied to control for differences in baseline [22]. The Incremental cost-effectiveness ratio (ICER) will be determined on the basis of incremental costs and effects of group-ST compared to TAU. The cost-effectiveness ratio will be stated in terms of costs per outcome rate,

the cost-utility ratio will focus on the net cost per QALY gained.
The ICER will be calculated as follows. ICER = (Ci – Cc) / (Ei – Ec), where Ci is the annual total cost of the group-ST, Cc is the annual total cost of TAU, Ei is the effect at 3-years follow-up for the group-ST and Ec is the effect at 3-years follow-up for TAU. The robustness of the ICER will be checked by non-parametric bootstrapping. Bootstrap simulations will also be conducted to quantify the uncertainty around the ICER, yielding information about the joint distribution of cost and effect differences. The bootstrapped cost-effectiveness ratios will be subsequently plotted in a cost-effectiveness plane, in which the vertical line reflects the difference in costs and the horizontal line reflects the difference in effectiveness. The choice of treatment depends on the maximum amount of money that society is prepared to pay for a gain in effectiveness, which is called the ceiling ratio. Therefore, the bootstrapped ICERs will also be depicted in a cost-effectiveness acceptability curve showing the probability that group-ST is cost-effective using a range of ceiling ratios.
Additionally, to demonstrate the robustness of our base-case findings a multi-way sensitivity analyses will be performed. In the sensitivity analysis uncertain factors of assumptions in the base case analysis are recalculated to assess whether the assumptions have influenced the incremental cost-effectiveness ratio (ICER), for example by varying cost-prices and volumes between minimum and maximum [23].
For the primary research question, the two formats are combined ("group-ST"). For the secondary research question, the 2 formats ST-A and ST-B are compared to eachother using the same methods, with ST-A replacing group-ST and ST-B replacing TAU.

QUALITATIVE STUDY

In-depth interviews (patients) and focusgroups (therapists) with member checks will assess patients' and therapists' opinions about treatment and preferred format of group-ST. Assessment will continue until saturation is reached, probably with 12-15 ST patients and 12-15 therapists. Qualitative methods are important adjuncts to quantitative methods, as they allow for the discovery of unexpected (unhypothesized) results, and give a say to the major stakeholders in the further developments - like how the final treatment protocol should be revised before it is implemented in daily practice. As an example, based on patient in-depths interviews we adapted the way imagery rescripting, an important ingredient of ST, is introduced to the patient[24-25]. In the present study we explicitly want to give patients and therapists a say about the to be prefered group-ST format: A or B.

SYSTEMATIC REVIEW

A systematic review resulting in a meta-analysis was done as preparation for this application. The complete report, presenting forest plots, is added as appendix.

Search terms
- population: borderline personality disorder
- intervention: schema (focused) therapy
- comparison/control: no related terms used
- outcome: id.
- methodologcal filters: id.

Databases used (and number of ms. retrieved)
- Psychinfo (233)
- Medline (22)
- Cochrane (125)

Selection procedure and validity assessment

Selection: Abstracts were read by the applicant and an independent research assistant, and independently selected if deemed relevant for the present systematic review. Disagreements/doubtful findings (3) were discussed and resolved by mutual agreement. To be selected, studies had to be RCTs, open trials, or consecutive case series with quantitative results, assessing the effects of schema (focused) therapy in BPD patients with a primary focus on reducing personality disorder dysfunction. Studies primarily focusing on other disorders but that included participants with comorbid BPD were excluded. Case studies of a single patient were also excluded as this design doesn't protect for selection biases. We added our own pilot study into the combination of group and individual ST for BPD[11].

Validity check: The results were compared to a recent qualitative review of schema therapy[28]. All studies found with our searches were discussed in that review, and the review did not yield additional studies meeting our selection criteria.

Results

- clinical outcome: In total 6 relevant studies were detected. Four were RCTs[4-7, 29], one a consecutive case series[30], and one an open trial[11]. All studies employed structured diagnostic interviews for diagnosing BPD (5 studies SCID-2, 1 DIPD-R[4]). One study had only a minority of BPD diagnoses among the PD-patients, and did not assess full ST, but an adaptation[29]. Although this study only marginally meets the criteria, we included it in the meta-analysis (see appendix), because there were only 3 RCTs comparing ST for BPD to non-ST treatments. Moreover, its inclusion prevents an overoptimistic effect estimation. Between condition effect sizes varied from .5 (for the incomplete ST [29]), via 1.1 [5] to 2.7 [4]. The weighted mean effect size was 1.28, 95%CI (.4, 2.2), in favor of ST. Excluding [29] leads to a weighted between group effect size of 1.8 (95%CI .3, 3.4) in favor of ST.

The 5 studies focusing on BPD only report highly similar findings on primary outcome (BPD severity, 4-7,11) or the average over outcomes [30 had no primary outcome]: pre-post change effect sizes d were in the 2-3 range, weigthed mean 2.5, 95%CI (2.2-2.8).
Having access to the data, we could calculate within group effect sizes for reduction of BPD severity in 1 year of treatment in group-ST [4,11] and individual ST [5,7]. For group-ST the mean effect size was larger than 2 (Cohen's d); for individual ST approximately 1.25. This indicates that changes take place earlier in group than in individual ST.

- economic evaluation: Two relevant studies were retrieved, one done by our own group[6], comparing cost-effectiveness and cost-utility between individual ST and a psychodynamic treatment (TFP). This study was already discussed. The other, with our group also involved, is the cost-effectiveness study of individual ST with and without extra crisis support[31]. Preliminary results indicate no support for extra telephone support both in terms of costs and outcomes. The element was therefore deleted from the protocols tested in the present study.

Summary and conclusions

Studies so far indicate that ST is an effective and safe treatment for BPD outpatients. The pre-post effect sizes are remarkably similar across studies, indicating that results can be replicated. Between condition effect sizes show more variability, probably caused by the different control conditions that varied from a very ineffective TAU [4] to a quite effective psychodynamic specialized treatment[5]. The two studies involving group-ST for BPD attain similar effects as individual ST in less time, suggesting that they speed up recovery processes.

The present study will add to the existing knowledge base:
- assessment of the degree to which the strong and relatively fast effects of group-ST are maintained when the treatment is given outside its developers' center
- assessment of the cost-effectiveness of group-ST. To date, no cost-effectiveness study of group-ST has been done (or is running). ST, whether individual or group, has not yet been compared to TAU in cost-effectiveness, limiting our knowledge about the potential value (group)-ST has for society.
- a direct comparison of two formats that have been developed for group-ST. This is important as format A is cheaper in direct delivery costs, but some doubt whether this format, that mainly consists of group sessions, meets the needs of BPD patients fully enough.
- giving the most important stakeholders, patients and therapists, a say about BPD treatment in general, further developments of group-ST, and in particular about the to be preferred format of group-ST.

TIME SCHEDULE

2010-2011: therapists training, patient recruitment, first treatments start, supervision, assessments
2012: recruitment complete, last treatment start, supervision, assessments, reliability checks, data check 2013: treatments, supervision, assessments, treatment integrity, first 3 yr follow-up, data check, qualitative study
2014: assessments, last treatments, 3 yr follow-up, data check, analysis of 2 year treatment, publications
2015: last 3 yr follow-up, data analyses, final report, publications.

Note. Support is asked for 2012-2015 (4 years). The study started in 2010 with support from Dutch Fund for Mental Health and Maastricht University.

This study takes place in collaboration with American, German and Australian researchers who execute the same comparisons of group-ST and TAU, and obtained their own funding. However, the Dutch study is by far the largest and has sufficient power to answer the research questions uniquely for the Netherlands, which is required by ZONMW. The international findings will be compared and analysed with help of meta-analytic or multilevel techniques. The present application pertains only to the Dutch study.

Expertise, voorgaande activiteiten en producten / Expertise, prior activities and products

Prof. dr. Arnoud Arntz is full professor of Clinical Psychology and Experimental Psychopathology, and director of the research center of Experimental Psychopathology at Maastricht University. He is an expert in the treatment of personality disorders (PDs) and co-developed new techniques integrated in ST. His research focusses on treatment and on the experimental study of psychological processes in PDs. The BPDSI, an internationally well-known instrument to assess BPD severity was developed by him. He was project leader of the multicenter trial comparing ST to TFP as treatment for BPD[1,5], and of a just completed large multicenter RCT (n=331) comparing ST, CCT and TAU as treatments for non-borderline PDs. He is also involved in an implementation study of ST for BPD[7]. All these studies assessed cost-effectiveness. Prof. Arntz contributed to the dissemination of ST by writing protocol chapters and books, setting up a Dutch register of ST-therapists (www.schematherapie.nl), and giving workshops. He is regularly invited to give keynotes at scientific conferences on the topics of BPD and ST.

Dr. Joan Farrell, PhD, is Clinical Director of the Center for BPD Treatment & Research at Larue Carter Hospital, Indiana University School of Medicine, Indiana USA. She spent the last 20 years of her career on developing effective group ST for BPD. This included conducting a successful RCT of Group ST for outpatients added to treatment as usual[4], establishing a BPD treatment and research center in Indianapolis, developing a dedicated BPD inpatient unit in a university affiliated medical school and with Dr. Fretwell establishing an outpatient group ST program in a local community mental health center.
Dr. Farrell was on the NIMH committee that examined the conduct of treatment trials of BPD[12]. She led an international

group-ST protocol development workgroup, and is first author of the book presenting this group-ST protocol to be published by Wiley. She trained and will supervise all study therapists.

Dr. Gerard van Breukelen is a associate professor in Statistics at Maastricht University. He is a specialist in in the design and analysis of randomized controlled trials with a complicated data structure due to nesting (cluster randomized and multisite/ multicentre trials) and/or repeated outcome measurements. He has published various papers on the optimal design and sample sizes for such trials in statistical journals, and he is co-author to several RCT publications in clinical psychology, public health, medicine and social psychology. Statistical methods used include multilevel (mixed) regression and structural equation modeling, and Gerard teaches these topics to research master and PhD students at Maastricht University, The Netherlands.

Dr. Math J.J.M. Candel is assistant professor Methodology and Statistics at Maastricht University. His research focuses on optimal designs for research in Health Sciences and Medicine. He wrote several international publications on optimal designs and various other statistical topics. He has broad experience in statistical consultancy, and acted as a co-author in various applied studies. As a course coordinator and a lecturer he is responsible for and involved with several bachelor and several master courses given at the Faculty of Health, Medicine and Life Sciences at Maastricht University.

Dr. Silvia Evers is a associative professor in Health Economics. She has ample experience with conducting and analyzing cost-effectiveness of treatments of mental health problems. She is HTA-supervisor in several clinical trials and lecturer in HTA-courses, especially in the Research Master on HTA (www.hsrm.nl). Her research focus is on the methodology of economic evaluation, meta-analysis and quality of life analysis. She has a special interest in the application of these methods in the field of mental disorders and brain related diseases. Dr. Evers is a member of several National and International working groups and referee for journals and research programs, and member of the Mental Health Economics Europe Network that aims to develop refined methodologies of mental health economics in Europe. She was senior health economist in the nearly completed RCT on the (cost)effectiveness of ST for 6 PDs other than BPD, with 12 centers and N=331. This multicenter RCT investigated similar cost-effectiveness issues as the present proposal.

Michiel van Vreeswijk, MSc is clinical psychologist and CEO of G-kracht, a mental health institute. He is a certified CBT therapist (VGCt) and certified supervisor schematherapy (ISST, Dutch register ST) and is an expert in group ST. He regularly gives workshops and supervision in (group)ST. He is member of the board of the Dutch ST register, and member of the international workgroup that developed the group-ST protocol to be tested in the present study. He is currently doing research on short term group ST.

Hannie van Genderen, MSc, is a registrered Clinical Psychologist and Psychotherapist at the Maastricht Community Mental Health Center. She is an experienced ST therapists with a specialization in BPD, and a registrered trainer and supervisor of ST. She developed a training programmes in ST for BPD and cluster-C PD, and trained hundreds of therapists in ST, in the Netherlands and abroad. She collaborates with prof. Arntz for over 20 years, and wrote a protocol book on ST for BPD together with him. She collaborated with Farrell and van Vreeswijk in the international workgroup that developed the group-ST protocol to be tested in the present study. She also developed a group-ST training for Dutch therapists, and started to disseminate this new treatment approach.

Relevant ST products:

Protocol books:

Arntz A & van Genderen H (2009). Schema Therapy for Borderline Personality Disorder. Chichester: Wiley.

Arntz A (2010). Schematherapie bei Cluster-C Persönlichkeitsstörungen. In: E Roediger & G Jacob (Eds), Fortschritte der Schematherapie, 146-82. Göttingen: Hogrefe.

Farrell J, Shaw, I, Vreeswijk M van, Genderen H van et al (2011). Group Schematherapy for Borderline PD. Wiley: in prep.

Genderen van H, & Arntz, A (2010). Schematherapie bij borderline persoonlijkheidsstoornis. Amsterdam: Nieuwezijds.

Jacobs G & Arntz A (2011). Schematherapie in der Praxis. Beltz.

Arntz A, et al (2011). Schematherapie in de praktijk. Amsterdam: Nieuwezijds. In prep.

Rijkeboer M Genderen van H & Arntz A (2009). Schemagerichte therapie. In EHM Eurelings-Bontekoe et al (Eds), Handboek persoonlijkheidspathologie, 301-18. Houten: BSL.

Vreeswijk MF van & Broersen J (2006). Schemagerichte therapie: werken in groepen. Houten: BSL.

Vreeswijk MF van, et al (Eds) (2008). Handboek schematherapie. Theorie, praktijk en onderzoek. Houten: BSL.

Vreeswijk MF van et al (Eds) (2011). Handbook Schema Therapy. Chichester: Wiley, in prep.

Others:

Arntz A et al(2003/2010). Borderline Personality Severity Index (BPDSI). Maastricht: author.

Arntz A (2009). Schematherapie: een nieuwe therapie voor chronische problematiek. De Psycholoog, 44, 404-6.

Arntz A et al. Schema Mode Inventory-2. Maastricht: author.

Bamelis L, Giesen-Bloo J, Bernstein D, & Arntz A (2010). Effektivitätsstudien zur Schematherapie. In: E Roediger & G Jacob (Eds), Fortschritte der Schematherapie, 86-103. Göttingen: Hogrefe.

Nadort et al. DVD series: Schema Therapy for BPD.

Dutch Register Schema Therapy: www.schematherapie.nl

Publicaties / Publications

-Arntz A (2004) Cognitive therapy for Borderline Personality Disorder. In Beck et al (Eds), Cognitive Therapy for PDs. New York: Guilford
-Arntz A et al (2004) Beliefs in Personality Disorders. Behav Res Ther, 2, 1215-25.
-Arntz A & van Genderen (2009) Schema Therapy for Borderline Personality Disorder. Chichester: Wiley
-Arntz A et al (2009) Taxometric evidence for the dimensional structure of cluster-c, paranoid, & borderline PDs. J Personal Dis 23, 606-8
-Arntz A et al (2009) Theory of mind in borderline and cluster-C PD. J Nerv Mental Dis 197, 801-7
-Arntz A et al (2003) Reliability and validity of the BPDSI. J Pers Dis 17, 45-49
-Arntz A, Weertman A (1999) Treatment of childhood memories: theory and practice. Beh Res Ther 37, 715-40
-Asselt van A, Dirksen CD, Arntz A et al (2007) The cost of BPD: societal cost of illness. Eur Psychiatry 22, 354-61
-Asselt van A, Dirksen CD, Arntz A et al (2008) Outpatient psychotherapy for BPD: Cost-effectiveness of SFT vs TFP. Br J of Psych 192, 450-7
-Asselt van A, Arntz, A et al (2009) The EQ-5D: a useful QoL measure in BPD? Eur Psychiatry 24, 79-85
-Asselt van A (…) Arntz A et al (2009) How to deal with cost differences at baseline. PharmacoEconomics 27, 519-28
-Breukelen van GJP et al (2007) Relative efficiency of unequal vs equal cluster sizes in cluster randomized and multicentre trials. Stat in Med 26, 2589-603
-Breukelen van GJP et al (2008) Relative efficiency of unequal cluster sizes for variance component estimation in cluster randomized and multicentre trials. Stat Meth in Med Res 17, 439-58
-Broek van den MAJ (…) Breukelen van GJP et al (2009) Feasability of randomized controlled trials in liver surgery using surgery related mortality or morbidity as endpoint. Br J of Sur 96, 1005-14
-Broersen J & Vreeswijk MF van (2006) Schemagerichte therapie: werken in groepen. Houten: BSL
-Candel MJJ, Breukelen van GJP (2010) Sample size adjustments for varying cluster sizes in cluster randomized trials with binary outcomes analyzed with second-order PQL mixed logistic regression. Stat in Med 29, 1488-501
-Candel MJJM, Breukelen van GJP (2010) D-optimality of unequal versus equal cluster sizes for mixed linear regression analysis of randomized trials with clusters in one treatment arm. Comp Stat Data Anal 54, 1906-20
-Candel MJJM (2004) Performance of empirical Bayes estimators of random coefficients in multilevel analysis: Some results for the random intercept-only model. Stat Neerl 58, 197-219
-Candel MJJM (2007) Empirical Bayes estimators of the random intercept in multilevel analysis: Performance of the classical, Morris and Rao version. Comp Stat Data Anal 51, 3027-40
-Eurelings-Bontekoe EHM (…) Vreeswijk van MF et al (2010) Relationship between personality organization and Young's cognitive model of personality pathology. Pers Ind Diff 49, 198-203
-Evers S et al (2007) The Cost-Effectiveness of Pneumococcal Vaccination For Preventing Invasive Pneumococcal Disease In The Elderly (…) Eur J of Clin Micro Inf Dis 26, 531-40
-Evers S et al (2005) Criteria List For Assessment Of Methodological Quality Of Economic Evaluations-CHEC Int J of Tech Ass in H Care 21, 240-5
-Evers S (2010) Value of integrated care: revival of the monetary valuation of health care benefits. Int J Integr Care 10:e045
-Farrell JM et al (2009) A schema-focused approach to group psychotherapy for outpatients with BPD: A randomized controlled trial. J Beh Ther Exp Psych 40, 317-28
-Farrell JM, Shaw IA (1994) Emotional awareness training: A prerequisite to effective cognitive-behavioral treatment of BPD. Cogn Beh Pract 1, 71-91
-Gerhards SA, Arntz A, Evers SM et al (2010) Economic evaluation of online computerised CBT without support for depression in primary care: randomised trial. Br J Psychiatry 196, 310-8
-Gerhards SA, Abma TA, Arntz A, de Graaf LE, Evers SM, et al (2010) Improving adherence and effectiveness of computerised CBT without support for depression: A qualitative study. J Affect Disord. Oct 1.
-Gerhards SA, Evers SM, et al (2010) Discrepancy in rating health-related quality of life of depression between patient and general population. Qual Life Res
-Giesen-Bloo J & Arntz A (2005) World assumptions and the role of trauma in BPD. J Behav Ther Exper Psychiatry, 36, 197-208
-Giesen-Bloo J (..) Arntz A (2006) Outpatient psychotherapy for BPD: Randomized trial of SFT vs TFP. Arch of Gen Psych 6, 649-58
-Graaf de E, Gerhards S, Evers S, Arntz A et al (2008) Clinical and cost-effectiveness of computerized CBT for depression in primary care: design of a randomized trial BMC Publ H, 8, 224
-Grauvogl A, Evers SM, et al (2009) Research into the efficacy and cost-effectiveness of brief, free of charge and anonymous sex counselling to improve (mental) health in youth: design of a RCT. BMC Public H, 9:459
-Hendriks MRC (…)Evers SMAA, et al (2005) Effectiveness and cost-effectiveness of a multidisciplinary intervention programme to prevent new falls and functional decline among elderly persons at risk: design of a replicated RCT. BMC Publ H, 5:6
-Hoving MA, Evers SMAA, et al (2008) Intrathecal baclofen in children with spastic cerebral palsy: a cost-effectiveness study.

Dev Med Child Neur 50, 450-5
-Jacob G, Arntz, A (2011) Schematherapie in der Praxis. Hemsbach: Beltz
-Jacob G, (…) Arntz A. (2011) Comparison of different strategies to decrease negative affect and increase positive affect in women with borderline personality disorder. Beh Res Ther 49, 68-73
-Lobbestael J, Leurgans M, & Arntz A (2011) Inter-rater reliability of the Structural and Clinical Interview for DSM-IV axis (SCID I) and axis 2 disorders (SCID II). Clin Psych Psycho 18, 75-9
-Lobbestael J, Vreeswijk van MF, & Arntz A (2007) Shedding light on schema modes: A clarification of the mode concept and its current research status. Neth J of Psych, 76-85
-Lobbestael J, Vreeswijk van MF, & Arntz A (2008) An empirical test of schema mode conceptualizations in PDs. Beh Res Ther 46, 854-60
-Lobbestael J, Vreeswijk van MF, Spinhoven P, Schouten E, & Arntz A (2010) Reliability and Validity of the Short SMI. Beh Cogn Psych 38, 437–58
-Lobbestael J Arntz A et al (2010) Disentangling the relationship between different types of childhood maltreatment and personality disorders. J Personal Dis 24, 285-95
-Nadort M (…) Vreeswijk van MF & Arntz A (2009) Three Preparatory Studies for Promoting Implementation of Outpatient Schema Therapy for BPD in General Mental Health Care. Behav Res Ther 47, 938-45
-Nadort M, Arntz A et al (2009) Implementation of Outpatient Schema therapy for BPD with vs without crisis support by the therapist outside office hours: a randomized trial. Behav Res Ther 47, 961-73
-Sieswerda S, Arntz A et al (2005) Evaluations of emotional noninterpersonal situations by patients with BPD. J Behav Ther Exper Psychiatry 36, 209-25.
-Simpson SG, Morrow E, Vreeswijk van MF & Reid C (2010) Group Schema Therapy for Eating Disorders: A Pilot Study. DOI=10.3389/fpsyg.2010.00182
-Vreeswijk van MF, Broersen J, & Nadort M (2008) Handboek voor schematherapie. Houten: BSL
-Weertman A, Arntz A (2007) Effectiveness of Treatment of Childhood Memories in Cognitive Therapy for PDs: A controlled Study contrasting Methods Focusing on the Present and Methods Focusing on Childhood Memories. Beh Res Ther 45, 2133-43
-Zanarini MC, (…) Farrell J, Sanislow C (2010) Methodological considerations for treatment trials for persons with BPD. Ann of Clin Psych 22, 75-83

Referenties / References

References
1. Asselt van AD et al. The costs of Borderline Personality Disorder: Eur Psychiatry, 2007, 22, 354-61
2. Pompili M et al. Suicide in borderline personality disorder: a meta-analysis. Nordic J Psychiatry, 2005, 59, 319-24
3. Oldham JM Borderline personality disorder and suicidality. Am J Psychiatry, 2006, 163, 20-26
4. Farrell JM et al. A Schema-Focused Approach to Group Psychotherapy for Outpatients with Borderline Personality Disorder: A Randomized Controlled Trial. J Beh Ther Exp Psychiatry, 2009, 40, 317-28
5. Giesen-Bloo J et al. Outpatient psychotherapy for borderline personality disorder: randomized clinical trial of schema-focused therapy vs. transference-focused psychotherapy. Arch Gen Psychiatry, 2006, 63, 649-58
6. Asselt van AD et al. Outpatient psychotherapy for borderline personality disorder: cost-effectiveness of schema-focused therapy vs. transference-focused psychotherapy. Br J Psychiatry, 2008, 192, 450-57
7. Nadort MM et al. Implementation of outpatient schema therapy for borderline personality disorder (…) Behav Res Ther, 2009, 47, 961-73
8. Davidson K et al. The effectiveness of cognitive behavior therapy for borderline personality disorder: Results from the BOSCOT trial. J Personal Dis, 2006, 20, 450-65
9. Palmer S et al. The cost-effectiveness of cognitive behavior therapy for borderline personality disorder: Results from the BOSCOT trial. J Personal Dis, 2006, 20, 466-81
10. Perry et al. Effectiveness of Psychotherapy for Personality Disorders. J Am Psychiatry, 1999, 156, 1312-21
11. Dickhaut V & Arntz A. Pilot study of combined group-individual ST for BPD. In prep.
12. Kliem S et al. Dialectical Behavior Therapy for Borderline Personality Disorder: A Meta-Analysis Using Mixed-Effects Modeling. J Cons Clin Psychol, 2010, 78, 936–51
13. Linehan MM et al. Two-year randomized controlled trial and follow-up of dialectical behavior therapy vs therapy by experts for suicidal behaviors and borderline personality disorder. Arch Gen Psychiatry, 2006, 63, 757–66
14. Clarkin JF et al. Evaluating three treatments for borderline personality disorder: A multiwave study. Am J Psychiatry, 2007, 164, 922–28
15. McMain SF et al. A randomized trial of dialectical behavior therapy versus general psychiatric management for borderline personality disorder. Am J Psychiatry, 2009, 166, 1365–74
16. Pasieczny N & Connor J. The effectiveness of dialectical behaviour therapy in routine public mental health settings: An Australian controlled trial. Behav Res Ther, 2011, 49, 4-10
17. Gold M, Siegel J, Russell L, Weinstein M (Eds.) (1996). Cost-effectiveness in health and medicine. New York: Oxford University Press
18. Brooks R & EuroQol Group. Euroqol: the current state of play. Health Policy, 1996, 37, 53-72.
19. The EuroQol Group. EuroQol-a new facility for the measurement of health-related quality of life. Health Policy, 1990, 16, 199-208
20. Drummond MF et al. (2005). Methods for the Economic Evaluation of Health Care Programmes. Oxford: Oxford University Press
21. Oostenbrink JB et al. Manual for Cost Research. Methods and Unit Prices for Economic Evaluations in Health Care. College voor Zorgverzekeringen, 2004.
22. Manca A et al. Estimating mean QALYs in trial-based cost-effectiveness analysis: the importance of controlling for baseline utility. Health Econ, 2005, 14, 487-496
23. Briggs AH et al. Pulling cost-effectiveness analysis up by its bootstraps: a non-parametric approach to confidence

interval estimation. Health Econ, 1997, 6, 327-340
24. Arntz A. Imagery Rescripting for Personality Disorders. Cogn Behav Practice, in press
25. Schutz, M. et al. Personality Disorder patients' perspectives on the introduction of imagery within Schema Therapy: A qualitative study of patients' experiences. Cogn Behav Practice, in press
26. Arntz A et al. Reliability and validity of the BPDSI. J of Pers Dis, 2003, 17, 45-9
27. Giesen-Bloo et al. The BPDSI-IV: Psychometric evaluation and dimensional structure. Personality and Individual Differences, 2010, 49, 136-41
28. Bamelis L et al. Effectiveness studies of Schema Therapy. In: van Vreeswijk, M et al (Eds), Handbook Schema Therapy. Chichester: Wiley, in press.
29. Zorn P et al. Schemazentrierte emotiv-behaviorale therapie (SET): eine randomisierte evaluationsstudie an patienten mit Persönlichkeitsstörungen aus den clustern B und C. Verhaltenstherapie 2007, 17:233-41
30. Nordahl HM, Nysaeter TE. Schema Therapy for patients with BPD: a single case series. J Behav Ther Experiment Psychiatry, 2005, 36, 254-64
31. Nadort M, Arntz A et al. Implementation of outpatient schema therapy for borderline personality disorder: study design. BMC Psychiatry 2009, 9:64
32. Spinhoven P, Giesen-Bloo, JH Dyck van R & Arntz A. Can assessors and therapists predict the outcome of long-term psychotherapy in BPD? J Clin Psychol, 2008 64, 667-86
33. Zanarini M. Psychotherapy of borderline personality disorder. Acta Psychiatr Scand 2009, 120: 373–7
34. Lieb et al. Pharmacotherapy for borderline personality disorder: Cochrane systematic review of randomised trials. Br J Psychiatry, 2010 196, 4–12
35. Arntz et al. Central and Peripheral Nervous System Responses to Emotional Stimuli in Borderline Personality Disorder: Specificity and Effects of Psychotherapy. Under review.
36. Multidisciplinaire Richtlijn Persoonlijkheidsstoornissen. Utrecht: Trimbos Instituut, 2010.
37. Kloofstudie van de implementatie van de richtlijn persoonlijkheidsstoornissen in instellingen. In prep.

Financiële gegevens / Financial data

ZonMw budget

	Jaar / Year								
**Kostenpost**	*1*	*2*	*3*	*4*	*5*	*6*	*7*	*8*	**Totaal / Total**
Personeel	0	0	149,000	147,800	153,600	160,100	0	0	610,500
Materieel	0	0	13,800	3,800	3,700	3,700	0	0	25,000
Implementatie	0	0	20,000	0	0	0	0	0	20,000
Apparatuur	0	0	0	0	0	0	0	0	0
Overig	0	0	0	0	0	0	0	0	0
**Totaal / Total**	**0**	**0**	**182,800**	**151,600**	**157,300**	**163,800**	**0**	**0**	**655,500**

Co-financiering / Cofinancing

Naam co-financier / Name of cofinancier	Bedrag / Amount	Status
Fonds Psychische Gezondheid	40,000	Toegekend
Universiteit Maastricht	20,000	Toegekend

Bijzondere gegevens / Additional information

Vergunningen / Permits

	Verklaring nodig / Statement required?		Status verklaring / Statement status		
	Ja / Yes	Nee / No	Verkregen / Acquired	Aangevraagd / Applied	Nog niet aangevraagd / Not applied yet
METC	X		X		
DEC		X			
WBO		X			

Onderschrijvingen / Assents

	Ja / Yes	Nee / No	N.v.t. / N.A.
Code biosecurity / Code Biosecurity			X
Code openheid dierproeven / Code Transparency of Animal Testing			X

Study Design.

For 3 centers i, group-ST format A is given to half of the first cohort patients, group-ST format B to half of the second cohort patients. For 3 other centers j, the order is reversed (first ST-B; then ST-A). Order is randomized over the 6 centers.

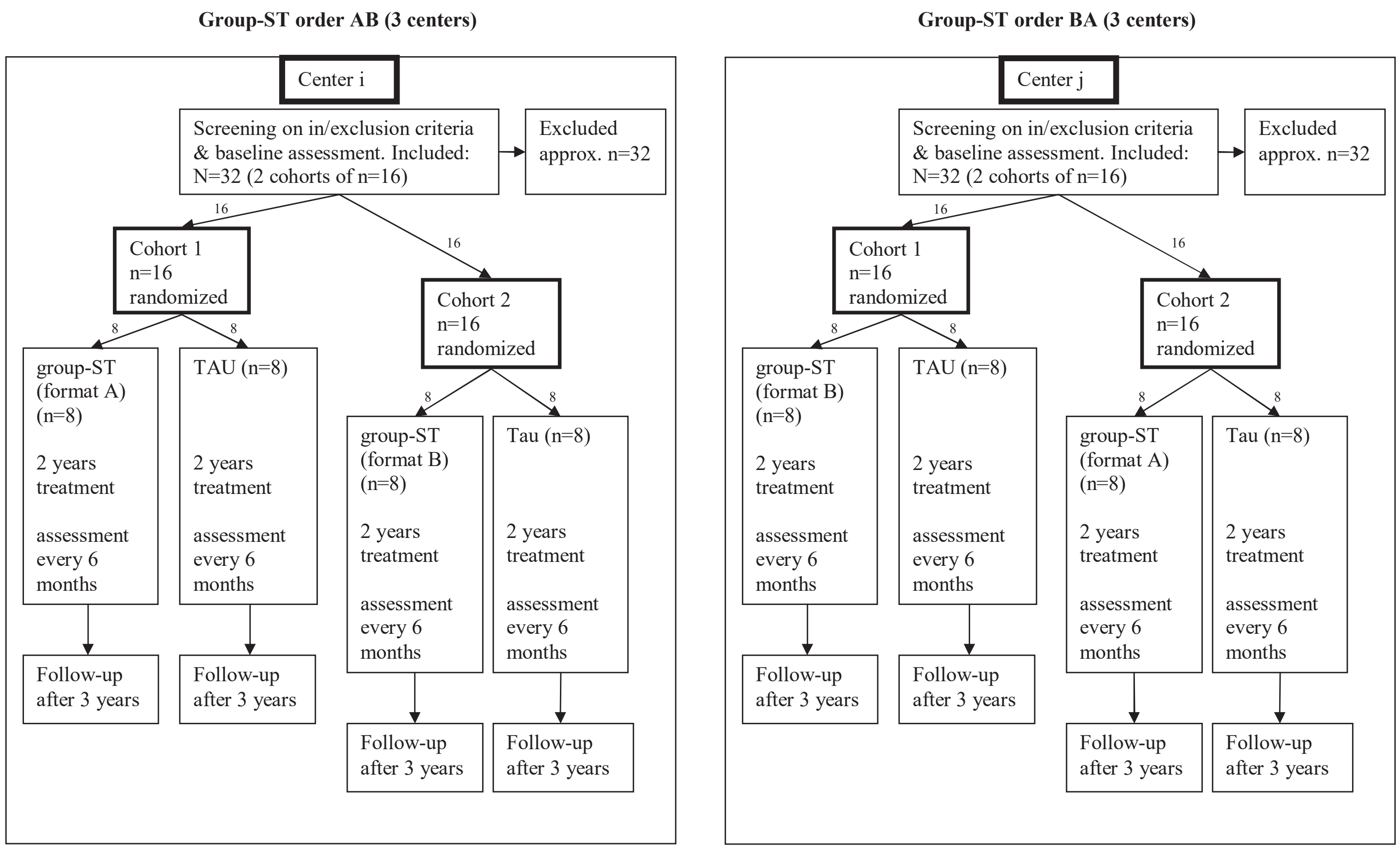

Systematic Review of Schema Therapy for Borderline Personality Disorder.

Arnoud Arntz & Michelle Houben
Maastricht University
Maastricht, February 3rd, 2011.

Search terms:

The first search in each database was done with the search terms: "schema focused therapy" AND "borderline personality disorder".
The second search was done with "schema focused therapy"
The third search was done with "schema focused therapy" AND "cluster b personality disorder"
The fourth-sixth searches were done with "schema therapy" replacing "schema focused therapy" in searches 1-3.
In Psychinfo and Medline, each of these 6 searches was initially done with the methodological filter "treatment outcome/clinical trial", and next repeated without that filter. All fields were searched.

-*Population:*
1. Borderline personality disorder,
2. Cluster-b personality disorder,
3. and no restriction.

-*Intervention:*
1. schema focused therapy
2. schema therapy

-*Comparison/control*
No restrictions were used.

-*Outcome measures*
No restrictions were used.

-*Methodological filters*
1. "treatment outcome/clinical trial" (Psychinfo)
2. "randomized controlled trial" (Medline)
3. each search was repeated without methodological filter

Databases
1. Psychinfo
2. Medline
3. Cochrane database

Selection Procedure, validity assessment

Abstracts of the articles and book chapters were read by the two authors, the second being an independent research assistant, and independently selected if deemed relevant for the present systematic review. Disagreements or doubtful findings (n=3) were discussed and decisions were made on mutual agreement. To be selected, the studies had to be RCTs, open trials, or consecutive case series with quantitative results, assessing the effects of schema (focused) therapy in Borderline Personality Disorder (BPD) patients with a primary focus on reducing personality disorder dysfunction. Studies primarily focusing on other disorders but that included

participants with comorbid BPD were excluded. Case studies of a single patient were also excluded as this design doesn't protect for selection biases.

We added our own pilot study into the combination of group- and individual ST for BPD (Dickhaut et al., in progress).

The results were compared to a recent qualitative review of schema therapy by Bamelis et al. (2011). All studies found with our searches were discussed in that review, and the review did not yield additional studies meeting our selection criteria.

Results

1. Psychinfo:

With the methodological filter "TREATMENT OUTCOME/CLINICAL TRIAL" 9 publications were detected. Without the filter 223. Fourteen articles seemed relevant, and were inspected in detail.

2. Medline:

With the methodological filter "randomized controlled trial" 6 publications were detected. Without the filter 22. Inspection of the abstracts yielded no additional study to the 5 already detected.

3. Cochrane:

The Cochrane searches yielded 125 publications. One possibly relevant article was not already detected by the previous searches, but was not included as the primary focus of this RCT was on opioid dependence (Ball et al., 2007).

4. Additions:

We added our own pilot study into the combination of group- and individual ST for BPD (Dickhaut et al., in progress). We also added the final findings by Nadort et al. (in preparation) who published intermediate results in 2009.

In total 6 relevant studies were detected. Four were RCTs, one a consecutive case series, and one an open trial. All studies employed structured diagnostic interviews for diagnosing BPD (5 studies SCID-2, 1 (Farrell et al) DIPD-R).
1. Giesen-Bloo et al. (2006) and van Asselt et al. (2008). This was a multicenter RCT comparing ST to Transference Focused Therapy (TFP) in BPD outpatients. ST was individual. Primary outcome was BPD severity, assessed with the BPDSI. ST was superior to TFP on primary outcome and on secondary outcome measures. The van Asselt et al. (2008) study reports the cost-effectiveness analysis of this study. ST was dominant to TFP: less costly and more effective.
2. Zorn et al. (2007; 2008). This was an RCT comparing group formats of a cognitive therapy format containing ST elements to social skills training for outpatients with various personality disorders, 20 of the total 93 patients had BPD. Many outcome measures were reported and no primary outcome measure was indicated. We therefore averaged effect sizes over the outcome instruments.
3. Farrell et al. (2009). This RCT compared Group-ST added to TAU, to TAU in a sample of 32 BPD outpatients. Strong effects of a limited dose of group-ST were found, and almost no effects for TAU. Main outcome was borderline severity assessed with the BSI.
4. Nadort et al. (2009). This RCT compared an individual ST condition with extra telephone support by the therapist in case of crisis to the same ST without that extra support, in the context of an implementation trial. Main outcome was BPD severity assessed with the BPDSI. No differences between the two ST conditions were found. For the present review we combined results from the two conditions at the end of

study (Nadort et al., in preparation) and used them for the within-ST meta analyses, as a between conditions comparison doesn't make sense with both conditions being ST..
5. Nordahl & Nysaeter (2005). A consecutive case series study with 6 BPD outpatients. Various outcome instruments were used, we used the average pre-post within group effect size for the within-ST meta analyses.
6. Dickhaut et al. (2011). An open trial of our group into the effectiveness of group-ST combined with individual ST for BPD outpatients. 18 months results were available, the 2 year results not yet. Primary outcome is BPD severity assessed with the BPDSI.

Meta analysis.
We based the meta analyses on intent to treat results. If a BPD severity index was available, that was taken as outcome. If it was not available, results were averaged across outcome measures (Nordahl & Nysaeter, 2005; Zorn et al., 2007). The relevance of the Zorn et al. study was viewed as limited given that only 21.5% of the participants had BPD, and the experimental treatment was not a full ST package. Given the small number of RCTs of ST vs. another treatment, the study was used in the between condition meta analysis. If anything, it contributed to a rather conservative overall effect size estimate, as it had a relatively small effect size. For the within ST we left it out of the analyses as data from 5 studies were available.

Meta analysis: ST vs. control treatments.
Three RCTs compared ST (or a variant, Zorn et al. 2007) to a control treatment. One to TFP (Giesen-Bloo et al., 2006), one to social skills training (Zorn et al. 2007), and one to treatment as usual (TAU; Farrell et al, 2009). A forest plot of the between condition effect sizes (Hedges g) is depicted below. Differences between treatments probably relate to (i) differences between control treatments (TAU as the least effective (within ES ≈ 0), TFP as relatively the most effective (within ES > 1); (ii) and the fact that the Zorn et al study did not include a full ST package.

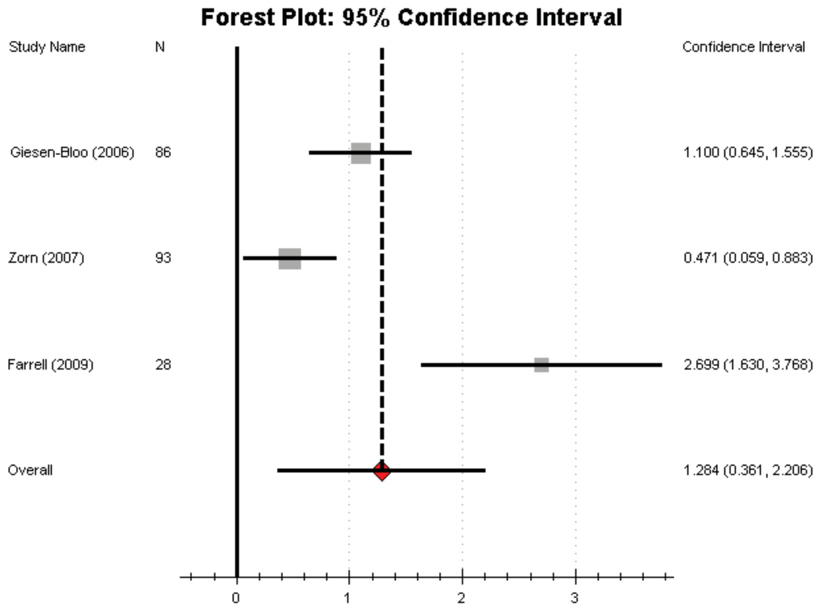

Figure 1. Forest plot of between condition effect sizes (Hedges g) of ST vs. control treatment of 3 RCTs. Positive values denote superior effects of ST. Random effects model. Analyzed with MetaAnalyst (Beta 3.13).

Meta analysis: within ST changes.
We derived within ST effect sizes from 5 studies that included only BPD outpatients (pre-post effect sizes, Cohen's d). Figure 2 depicts the Forest plot.

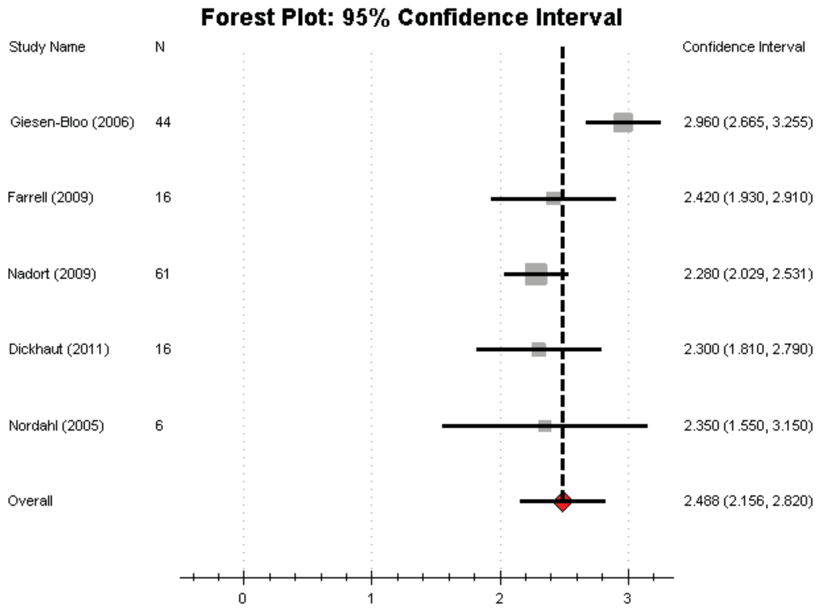

Figure 2. Forest plot of within condition effect sizes (Cohen's d) of 5 ST trials.. Positive values denote improvement. Random effects model. Analyzed with MetaAnalyst (Beta 3.13).

The within condition effect sizes are quite similar, indicating that ST has quite stable effects. Differences between RCTs in between condition effect sizes (figure 1) seem therefore more attributable to differences in the comparison groups. Note that the two studies involving group-ST (Farrell et al., 2009; Dickhaut et al., 2011) had much shorter treatment times (up to 18 months) than the studies investigating individual ST (up to 3 years).

Conclusion.

Studies so far indicate that ST is an effective and safe treatment for BPD outpatients. The pre-post effect sizes are remarkably similar across studies, indicating that results can be replicated. Between condition effect sizes show more variability, but that is probably associated with the different control conditions that varied from a very ineffective TAU (Farrell et al, 2009) to a quite effective psychodynamic specialized treatment (TFP, Giesen-Bloo et al., 2006). The two studies involving group-ST for BPD attain similar effects as individual ST in less time, suggesting that they speed up recovery processes.

The present study will add to the existing knowledge base:
- assessment of the degree to which the strong and relatively fast effects of group-ST are maintained when the treatment is given outside its developers' center (Farrell et al., 2009).
- assessment of the cost-effectiveness of group-ST. To date, no cost-effectiveness study of group-ST has been done (or is running). ST has not been compared to TAU in cost-effectiveness, limiting our knowledge about the potential value (group)-ST has for society.

- a direct comparison of two formats that have been developed for group-ST. This is important as format A is cheaper in direct delivery costs, but some doubt whether this format, that mainly consists of group sessions, meets the needs of enough BPD patients.
- giving the most important stakeholders, patients and therapists, a say about BPD treatment in general, further developments of group-ST, and in particular about the to be preferred format of group-ST.

References.

van Asselt, A. D. I., Dirksen, C. D., Arntz, A., Giesen-Bloo, J. H., Van Dyck, R., Spinhoven, P., et al. (2008). Outpatient psychotherapy for borderline personality disorder: cost effectiveness of schema-focused therapy versus transference focused psychotherapy. *British Journal of Psychiatry, 192*, 450–457.

Ball, S.A. (2007). Comparing individual therapies for personality disordered opioid dependent patients. *Journal of Personality Disorders, 21* (3), 305-321

Bamelis, L., Giesen-Bloo, J., Bernstein, D., & Arntz, A. (2011). Effectiveness studies of Schema Therapy. In: van Vreeswijk, M., Broersen, J. & Nadort, M. (Eds.), *Handbook Schema Therapy.* Chichester: Wiley, in press.

Dickhaut, V., Arntz, A. (2011). Individual and group schema therapy combined as treatment for borderline personality disorder: An open trial. *In preparation.*

Farrell, J.M., Shaw, I.A., & Webber, M.A. (2009). A schema-focused approach to group psychotherapy for outpatients with borderline personality disorder: a randomized controlled trial. *Journal of Behavior Therapy and Experimental Psychiatry, 40,* 317–328.

Giesen-Bloo, J., van-Dyck, R., Spinhoven, P., van-Tilburg, W., Dirksen, C., van-Asselt, T., et al. (2006). Outpatient Psychotherapy for Borderline Personality Disorder: Randomized Trial of Schema-Focused Therapy vs Transference-Focused Psychotherapy. *Archives of General Psychiatry, 63,* 649-658.

Nadort, M., Arntz, A., Smit, J. H., Wensing, M., Giesen-Bloo, J., Eikelenboom, M., et al. (2009). Implementation of Outpatient Schema therapy for Borderline Personality Disorder with versus without crisis support by the therapist outside office hours: a randomized trial. *Behaviour Research and Therapy, 47(11),* 961-973.

Nordahl, H. M., & Nysaeter, T. E. (2005). Schema Therapy for patients with borderline personality disorder: a single case series. *Journal of Behavior Therapy and Experimental Psychiatry, 36*, 254-264.

Zorn, P., Roder, V., Muller, D. R., Tschacher, W., & Thommen, M. (2007). Schemazentrierte emotiv-behaviorale therapie (SET): eine randomisierte evaluationsstudie an patienten mit persönlichkeitsstörungen aus den clustern B und C. *Verhaltenstherapie, 17*, 233-241

Zorn, P., Roder, V., Soravia, L., Tschacher, W., (2008) Evaluation der 'schemazentrierten emotiv-behavioralen therapie' (SET) für patienten mit persönlichkeitsstörungen: Ergebnisse einer randomisierten untersuchung. *Psychotherapie Psychosomatik Medizinische Psychologie, 58,* 371-378.
