# Supplementary material for: Effectiveness of Predominantly Group Schema Therapy and Combined Individual and Group Schema Therapy for Borderline Personality Disorder: A Randomized Clinical Trial
Source: JAMA Psychiatry. 2022 Mar 2;79(4):287–99. doi: 10.1001/jamapsychiatry.2022.0010 (PMC8892362; doi:10.1001/jamapsychiatry.2022.0010)
Supplement: Supplement 2. — eAppendix 1. Overview of Sites and Cohorts per Site eAppendix 2. Deviations From the Original Plan eTable 1. Overview of Type and Frequency of Sessions Over 2 Years for PGST and IGST eTable 2. Overview of Initial TAU per Site eAppendix 3. Assessment of Treatment Integrity eAppendix 4. Clinical Outcome Measures eAppendix 5. Overview of Random Effects for Secondary Outcomes eAppendix 6. Estimation Procedures for the Effect Size Cohen d and its 95% CI eTable 3. Results of Loc-f Sensitivity Analysis eTable 4. Sensitivity Analysis of the Zero-Offset Value for the Gamma Regression of the BPDSI Total Score eTable 5. Descriptive Statistics of Observed BPDSI Total Scores, GLMM Results in Transformed Scale, Pairwise Contrasts at 3 years in Original Scale, Treatment Retention per 3 Months by Treatment Arm eTable 6. BPDSI-Based Secondary Outcomes: BSPDI Subscales, Suicidality, Suicide Attempts [file jamapsychiatry-e220010-s002.pdf]

Supplementary Online Content

Arntz A, Jacob GA, Lee CW, et al. Effectiveness of predominantly group schema therapy and combined individual and group schema therapy for borderline personality disorder. *JAMA Psychiatry*. Published online March 2, 2022. doi:10.1001/jamapsychiatry.2022.0010

eAppendix 1. Overview of Sites and Cohorts per Site

eAppendix 2. Deviations From the Original Plan

eTable 1. Overview of Type and Frequency of Sessions Over 2 Years for PGST and IGST

eTable 2. Overview of Initial TAU per Site

eAppendix 3. Assessment of Treatment Integrity

eAppendix 4. Clinical Outcome Measures

eAppendix 5. Overview of Random Effects for Secondary Outcomes

eAppendix 6. Estimation Procedures for the Effect Size Cohen *d* and its 95% CI

eTable 3. Results of Loc-f Sensitivity Analysis

eTable 4. Sensitivity Analysis of the Zero-Offset Value for the Gamma Regression of the BPDSI Total Score

eTable 5. Descriptive Statistics of Observed BPDSI Total Scores, GLMM Results in Transformed Scale, Pairwise Contrasts at 3 years in Original Scale, Treatment Retention per 3 Months by Treatment Arm

eTable 6. BPDSI-Based Secondary Outcomes: BSPDI Subscales, Suicidality, Suicide Attempts

This supplementary material has been provided by the authors to give readers additional information about their work.

© 2022 Arntz A et al. *JAMA Psychiatry*.

Supplementary Materials

Overview

eAppendix 1. Overview of sites and cohorts per site

eAppendix 2. Deviations from the original plan

eTable 1. Overview of type and frequency of sessions over 2 years for PGST and IGST

eTable 2. Overview of initial TAU per site

eAppendix 3. Assessment of Treatment Integrity

eAppendix 4. Clinical outcome measures

eAppendix 5. Overview of Random Effects for Secondary Outcomes

eAppendix 6. Estimation procedures for the effect size Cohen d and its 95% CI

eTable 3. Results of Loc-f sensitivity analysis

eTable 4. Sensitivity analysis of the zero-offset value for the gamma-regression of the BPDSI-total score

eTable 5. Descriptive statistics of observed BPDSI total scores, GLMM results in transformed scale, pairwise contrasts at 3 years (in original scale), treatment retention per 3 months by treatment arm

eTable 6. BPDSI-based secondary outcomes: BSPDI subscales, suicidality, suicide attempts

© 2022 Arntz A et al. *JAMA Psychiatry*.

eAppendix 1. Overview of sites and cohorts per site

Overview of the 15 sites participating in the group-ST for BPD trial

Australia
Peel, WA: Peel Mental Health Service (Rockingham Peel Group (RkPG), WA, Australia)
Rockingham, WA: Rockingham Mental Health Service (Rockingham Peel Group (RkPG), WA, Australia)

Germany
Freiburg: Department of Clinical Psychology and Psychotherapy, Institute for Psychology, University of Freiburg, Freiburg, Germany
Hamburg: IVAH GmbH (Institute for Training in CBT), Hamburg, Germany
Lübeck: Klinik für Psychiatrie und Psychotherapie, University of Lübeck, Lübeck, Germany

Greece
Athens: 1st Department of Psychiatry, Eginition Hospital, Medical School, Athens University, Athens, Greece

the Netherlands
Amsterdam: De Viersprong, Amsterdam/Duivendrecht, the Netherlands
Heerlen: Mondriaan Mental Health Institute, Heerlen, the Netherlands
Helmond: Mental Health Institute GGZ Oostbrabant, Helmond, the Netherlands
Hilversum: Symfora, GGZ Centraal Mental Health Institute, Hilversum, the Netherlands
Maastricht: Community Mental Health Center Maastricht (RIAGG Maastricht), Maastricht, the Netherlands
Venlo: Vincent van Gogh Mental Health Institute, Venlo, the Netherlands
Venray: Vincent van Gogh Mental Health Institute, Venray, the Netherlands

UK
Bradford: Bradford District Care NHS Foundation Trust, Bradford, UK
London: Maudsley NHS Foundation Trust, London, UK

© 2022 Arntz A et al. *JAMA Psychiatry*.

		Cohort number					
Country	Site	1st	2nd	3rd	4th	5th	
Australia	Peel	PG-ST					
	Rockingham	IG-ST					
Germany	Freiburg	IG-ST	PG-ST				
	Hamburg	PG-ST	IG-ST				
	Lübeck	PG-ST	IG-ST				
Netherlands	Heerlen	IG-ST	PG-ST				
	Helmond	IG-ST	PG-ST				
	Hilversum	PG-ST	IG-ST				
	Maastricht	PG-ST	IG-ST	IG-ST			
	Amsterdam	PG-ST	IG-ST	PG-ST	IG-ST	PG-ST	
	Venray	PG-ST					
	Venlo	IG-ST					
UK	Bradford	IG-ST	PG-ST				
	London	PG-ST	IG-ST				
Greece	Athens	IG-ST	PG-ST				
COHORTS		as 1st	as 2nd	as 3rd	as 4th	as 5th	mean rank
# PG-ST	15	8	5	1	0	1	26
# IG-ST	15	7	6	1	1	0	26
TOTAL	30						

© 2022 Arntz A et al. *JAMA Psychiatry*.

eAppendix 2. Deviations from the original plan

Sites

The participating Australian mental health institute (Peel and Rockingham Kwinana Mental Health Service) ran the two cohorts in two different sites (Peel, Rockingham), which were therefore treated in the analyses as separate sites. Similarly, one Dutch mental health center (Vincent van Gogh Institute) ran the two cohorts in two different sites (Venlo, Venray), which were also treated as separate sites in the analyses.

The candidate sites in Ireland, Sweden, UK, and USA could not participate due to failure to obtain funding. One Dutch site withdrew due to logistic problems in the initial recruitment stage, before recruiting a complete cohort. Later, one Dutch, two British and one Greek site joined the study.

To reach the planned N and to reach a balanced order of cohort of ST-A vs ST-B arms, the Maastricht site ran one extra cohort, and the Amsterdam site 3 extra cohorts (see Supplement 1).

Treatment sessions

Although this is not a deviation from the original plan (on the contrary), there has been some incorrect information previously given about the number of treatment sessions in the ST arms.

The original trial registration (Netherlands Trial Register NL2266) stated 64 instead of 63 individual sessions in the combined individual-group ST treatment (IG-ST). This error has been corrected in the register.

The design ms.[1] also gives incorrect information, probably because of an overestimation of the number per weeks per year available for treatment. The correct numbers and the correct division over time are given in Supplement 2.

Secondary outcomes

Contrary to what was stated in the design ms. (Wetzelaer et al., 2014, p.8), but in line with the trial registration (Netherlands Trial Register NL2226 (NTR2392)), the Schema Mode Inventory (SMI) was not only administered in the ST arms, but also in the TAU arm. Therefore the SMI functional and dysfunctional scores were analyzed as secondary outcomes.

Social functioning: when grant application failed for the USA sites, the SAS-SR (Weissman & Bothwell, 1976), added to the original plan to meet a request by a grant reviewer to allow comparability to other studies conducted in the USA, was skipped, to not overburden the participants. Note that work and social functioning are already assessed with the SOFAS, the WSAS, and work/student status (i.e., having a paid job and/or being a student).

The Relationship Scales Questionnaire (RSQ), assessing adult attachment dimensions, and the Emotional Core Needs Inventory (ECNI), assessing the degree to which participants experience that core emotional needs are met in their present life, will be used for secondary studies of change processes in treatment. They are not reported as clinical outcomes in the present report.

© 2022 Arntz A et al. *JAMA Psychiatry*.

The EQ5D will be used to calculate utilities for the cost-utility analysis, which together with the cost-effectiveness will be separately reported.

Change in SCID-assessed diagnoses from baseline to 3-year follow-up will be subject of a separate paper.

Originally, treatment retention was not specified as a secondary outcome. However, it is usual to report treatment dropout (or retention) in reports of RCT's, as this information is needed to get a complete picture of the results, and – more specifically – of the acceptability/tolerability of the treatments. We therefore decided to do a multilevel analysis of treatment retention, with site (and if possible, cohort nested under site) as random factor.

The original plan did not specify the analysis of subscales of the BPDSI (of which the total score is the primary outcome), assessing each of the 9 BPD traits according to the DSM definition. To get a complete picture of the effects on the BPD traits, we explored treatment effects on each of the subscales and report them in Supplement 9.

We also decided to report issues related to safety and possible harmful effects. Although this was not prespecified, we follow the trend to report serious adverse events and negative side effects of treatments. First, given the high suicidality and high suicide risk associated with BPD, we analyzed and report suicidality and actual suicide attempts (Supplement 10). Next, we decided to report deaths (including suicides). Lastly, we decided to report deteriorations, defined as an increase in BPDSI total score ≥ 11.70 compared to baseline, based on the reliable change criterion of the BPDSI.[2]

Statistical analysis

Originally, country was proposed as additional fixed factor in the analysis. However, we later realized that because both ST-A and ST-B are provided in each country, the addition of country makes little sense. Therefore in the published design ms. this was no longer part of the analytic plan.[1] A check on primary outcome learned that including country as fixed factor did indeed not change conclusions, whereas the fit of the model significantly deteriorated after including country (p=.017). For results, see next paragraph.

Effects of possible predictors and moderators, such as baseline severity and medication use will be explored in a separate paper, and not in the present RCT outcome ms.

Results for primary outcome controlling for country

Results of analysis of primary outcome controlling for country as fixed factor, with for the cohort nested under site level random effects of treatment and time (an additional random effect of treatment for site was redundant).

1. ST vs TAU

The reduction in BPDSI total scores remains significantly larger in the combined ST arms than in the TAU arm, p = .002.

Fixed Effects[a]				
Source	F	df1	df2	Sig.

© 2022 Arntz A et al. *JAMA Psychiatry*.

Corrected Model	37.379	7	69	.000
Country	3.970	4	68	.006
Time	226.813	1	33	.000
Con2_C	.107	1	82	.744
**Con2_C * Time**	**10.077**	**1**	**1023**	**.002**
Probability distribution: Gamma<br>Link function: Log				
a. Target: BPDSI_Total_01				

2. ST-A, ST-B and TAU

As can be seen, ST-B is significantly different from TAU in reduction of the primary outcome as assessed with the BPDSI, while ST-B is not.

Fixed Effects[a] (Reference = TAU)				
Source	F	df1	df2	Sig.
Corrected Model	28.564	9	80	.000
Country	3.913	4	68	.006
Time	204.992	1	58	.000
STA	.151	1	80	.699
STB	.019	1	80	.890
Time * STA	.954	1	918	.329
**Time * STB**	**15.043**	**1**	**923**	**.000**
Probability distribution: Gamma<br>Link function: Log				
a. Target: BPDSI_Total_01				

As can be seen, ST-B is significantly different from ST-A (and from TAU) in reduction of the primary outcome as assessed with the BPDSI.

Fixed Effects[a] (Reference = ST-B)				
Source	F	df1	df2	Sig.
Corrected Model	28.564	9	77	.000
Country	3.913	4	68	.006
Time	145.433	1	47	.000
STA	.047	1	79	.829
TAU	.019	1	80	.890
**Time * STA**	**4.797**	**1**	**467**	**.029**
**Time * TAU**	**15.043**	**1**	**923**	**.000**
Probability distribution: Gamma<br>Link function: Log				
a. Target: BPDSI_Total_01				

© 2022 Arntz A et al. *JAMA Psychiatry*.

References

[1] *Wetzelaer P, Farrell J, Evers SMMA, et al. Design of an international multicentre RCT on group schema therapy for borderline personality disorder. BMC Psychiatry 2014; 14: 319.*

[2] Giesen-Bloo J, van Dyck R, Spinhoven P, et al. Outpatient Psychotherapy for Borderline Personality Disorder: Randomized Trial of Schema-Focused Therapy vs Transference-Focused Psychotherapy. *Archives of General Psychiatry* 2006; **63**(6): 649-58.

© 2022 Arntz A et al. *JAMA Psychiatry*.

eTable 1. Overview of type and frequency of sessions over 2 years for PGST and IGST.

CONDITION	TYPE	NUMBER of SESSIONS	24 MONTHS OF TREATMENT			
			Months 1-12	Months 13-18	Months 19-21	Months 22-24
**PG-ST: GROUP EMPHASIS**	Group ST	118	2x week group (88)	Weekly group (22)	Bi-weekly (5)	Monthly (3)
	Individual ST	4-17	2 plus 10 banked	Bank of 3 sessions	1	1
	**TOTAL**	**122-135**				
**IG-ST: BALANCED INDIVIDUAL & GROUP**	Group ST	63	Weekly group (44)	*Bi-weekly grp (11)	Bi-weekly (5)	Monthly (3)
	Individual ST	61	Weekly (44)	*Bi-weekly (11)	Monthly (3)	Monthly (3)
	**TOTAL**	**124**		*group & individual should alternate so that there is 1 session per week of some treatment		

Note. Because of holidays and other reasons for absence of therapists and patients, 44 of 52 annual weeks were planned.

© 2022 Arntz A et al. *JAMA Psychiatry*.

eTable 2. Overview of initial TAU per site.

	n(TAU)	None[1]	medication only	psycho-dynamic	TFP	MBT	CCT	STEPPS	DBT[2]	CBT	Mindfull-ness	Supportive	CAT	Other	MISSING
Amsterdam	46								46						0
Athens	16	2		14[3]											0
Bradford	17								16					1	0
Freiburg[4]	16	4	2	2					3	4					1
Hamburg[4]	17			1						16					0
Heerlen	15		2	1		2		3		1	1	4			1
Helmond	16					1	1				1	11		2	0
Hilversum	15			2				6	4			2			1
London	15			4		2					3	3	2	1	0
Lübeck[4]	16								13	3					0
Maastricht	26				6	6	1			2	2	5		2	2
Peel	8	1							3					4	0
Rockingham	8	2	2						3		1				0
Venlo	7	1						1	2	2					1
Venray	8					1		7							0
Total	246	10	6	24	6	12	2	17	90	28	8	25	2	10	6

Note. Initial TAU that was started by participant. Could be followed by other treatment(s) if participant ended this treatment.

TAU = treatment as usual; TFP = Transference Focused Psychotherapy; MBT = Mentalization Based Treatment; CCT = Client Centered Therapy; STEPPS = Systems Training for Emotional Predictability and Problem Solving; DBT = Dialectical Behavior Therapy; CBT = Cognitive Behavior Therapy; CAT = Cognitive Analytic Therapy.

[1]Includes refusal of further participation or moving to another city etc., before TAU was decided.

[2]In Amsterdam and for one cohort in Lübeck, the DBT time frame, format and dosage was matched to the combined individual and group-ST (IG-ST) format. In other cases, DBT was delivered in group and individual format weekly for the first year and then in the second year a 6- or 12-months additional program was offered as clinically indicated. In some sites DBT was supplemented with other therapy modalities.

[3]In Athens: psychodynamic group provided twice weekly in year 1 and once weekly in year 2

[4]In German sites that were not DBT, treatment was up to 80 (individual) sessions over two years.

© 2022 Arntz A et al. *JAMA Psychiatry*.

eAppendix 3. Assessment of Treatment Integrity.

Practical barriers made video-recording of TAU largely impossible. For example, some patients were seen in private practices with therapists not involved in the study; others participated in routine group treatments with non-trial participants that refused recording. We designed a self-report instrument on which participants rated to what degree items were characteristic of their treatment, the Treatment Integrity Scale (TIS) (see below). The internal consistency of the nonspecific and specific subscales were good (.84,.87), and subscales discriminated between ST and TAU, especially the specific subscale (Table 1, main text), Cohen's d .34, .90.

Treatment integrity of group-ST was assessed by independent trained raters who viewed randomly selected video-recordings of 85 sessions across the five countries. Recordings were chosen from early, middle, and late stages of therapy and were rated on 28 competency subscale items of the Group Schema Rating Scale-Revised.[12] Interrater agreement (ICC) ranged between .74 and .98. Overall competence was rated between 'good' and 'very good' with a mean rating of 4.89 (SD .64) on a 7-point scale from 0 (very poor) to 6 (excellent).

Treatment integrity of individual ST was assessed by independent trained raters who judged 101 randomly selected recordings on a previously used scale.[3] On average, 16 ST-elements were detected per recording (SD 3.7; ICC .59). The mean competence by which the techniques were applied rated on a 0-6 scale (0=bad/harmful, 2=unsatisfactory, 4=good, 6=excellent) was 4.6 (SD .9; ICC .79), and the mean rating of general therapist competence was 4.7 (SD 1.1; ICC .65), with 79% of recordings rated >4 for techniques and 83% >4 for therapist competence.

© 2022 Arntz A et al. *JAMA Psychiatry*.

Treatment Integrity Scale
One therapist version

Instruction

This questionnaire is designed to gain insight into how you experience your treatment.

It is asked how the therapist behaves and what techniques are used in treatment. Please circle the number that matches the correct answer.

Everything you enter here is confidential and is not passed on to your therapist(s).

It might be that you don't recognize something, this is no problem. In that case you can mark a 1 ("no or very little').

1	2	3	4	5	6	7
not at all or a little		somewhat, sometimes		considerably, regularly		extensively, very often

1. The therapist is warm to me and tries to create safety.

2. The therapist is detached. *

3. The therapist cares about me.

4. The therapist senses my feelings and shows understanding of my experiences and feelings.

5. The therapist is critical to me.*

6. If a particular pattern of thoughts and feelings dominates me, then this is pointed out in therapy as a side (mode) of mine and I am helped to recognize this side.

7. The therapist relates to me as a sort of good parent, for example by:
   a. being available for me when I need him/her (outside regular sessions, eg via email, or by an additional session if needed)
   b. meeting my needs
   c. understanding and respecting my feelings, needs and opinions
   d. advising me if I need that
   e. letting me feel connected to him / her

8. The therapist relates a specific problem in my life to one part or side of me, and explores how that relates to my childhood experiences.

9. In therapy I learn to put into words the different sides of me.

10. If I experience a strong emotion, I learn in therapy to relate this change in emotion to a part or side of me.

© 2022 Arntz A et al. *JAMA Psychiatry*.

11. The therapy is focused on learning to control emotions.

12. In therapy we do exercises during which I imagine a situation from the past, present or future and I am asked for my needs and experience of this situation.

13. The therapy is talking, there are no exercises done. *

14. The therapy is focused on the present - there is no attention to my youth. *

15. Nasty (traumatic) experiences of my youth are processed in therapy with imagery exercises, role playing, or multiple chair techniques, during which interventions are fantasized.

16. The therapist sets limits when I show behavior that is harmful or dangerous.

17. If the therapist discusses my awkward or unhealthy actions, he / she does so in a way that I like, without letting me feel bad (examples of such actions: not doing homework exercises, drinking too much, drug use, avoidance of healthy behaviors, impulsive actions, self-injury).

18. In therapy we use role playing to practice how to respond well to a situation, outside the therapy.

19. The therapist helps me to weigh pros and cons of sides or parts of me.

20. The therapist helps me understand how my unhealthy behavior (e.g. avoidance of feelings, avoidance of healthy behaviors, alcohol abuse, self-injury, unsafe sex, uncontrolled anger) is associated with sides or parts of me.

21. The therapist helps me to change behavior that is not good for me, for example by advising me how things can be handled differently or by teaching me skills.

22. In therapy I learn to find safety in my life.

* Reversed scoring.

Note.

Group version has “therapists” instead of “therapist”. If both individual and group treatment was provided, both versions were filled out and averaged. The table below gives the effect sizes of the difference between ST and TAU (Cohen’s d) per item. The item (15) on childhood trauma processing showed the highest d.

Items 1-5, 16-19, 21-22 should be present in ST but were hypothesized to be not very specific for ST (average d = .22). These formed the nonspecific subscale (internal consistency .84).

Items 6-10, 12-15 and 20 were hypothesized to be relatively specific for ST (average d = .69). These constituted the specific subscale (internal consistency .87).

Item 11 was hypothesized to be not ST (d=.03).

Item	Effect size d
1	0.12

© 2022 Arntz A et al. *JAMA Psychiatry*.

2*	0.41
3	0.27
4	0.06
5*	0.23
6	0.51
7	0.58
8	0.75
9	0.42
10	0.41
11	0.03
12	0.58
13*	0.65
14*	0.77
15	1.23
16	0.24
17	0.17
18	0.68
19	0.06
20	0.25
21	0.03
22	0.11
Total	0.68

© 2022 Arntz A et al. *JAMA Psychiatry*.

eAppendix 4. Clinical outcome measures.*

Borderline Personality Disorder Severity Index version IV (BPDSI-IV)

The primary outcome was severity of BPD, assessed with the Borderline Personality Disorder Severity Index, version IV (BPDSI-IV). The BPDSI-IV is a semi-structured interview containing 70 items based on the nine BPD dimensions described in DSM-IV, assessing frequency and severity of manifestations of BPD. The BPDSI-IV is a reliable and valid instrument, suitable for use as an outcome measure [1-5]. The 9 subscales represent dimensional assessments of each of the 9 BPD criteria according to the DSM, their factorial validity was supported by a confirmatory factor analysis [2]. The subscales have internal consistencies ranging from .67 to .93 (mean .81), the total scale an internal consistency of .96 (Cronbach's alfa) [2]. The subscales of the BPDSI-IV provide information on the severity of each of the nine dimensions of BPD. Interrater agreement proved to excellent, ICC's ranging from .98 to 1.00 for subscales and total scale [2]. The recall period for the BPDSI-IV is three months. The total score ranges from 0 to 90. From the BDPSI-IV the number of suicide attempts during the last 3 months was derived, as well as an index of suicidality by summing the items on suicidality (part of the subscale on (para)suicide).

BPD checklist

The BPD checklist is a self-report instrument that measures the burden of BPD manifestations as experienced by patients. Since the BPD checklist measures changes in subjective burden, it is complementary to the BPDSI-IV that measures changes in symptomatology objectively. It consists of 47 items based on the nine dimensions of BPD in DSM-IV and answers are scored on five point Likert scales. The total score, which was used in the study, is highly reliable, Cronbach's alfa = .92 in a BPD sample, .97 in a mixed sample [6]. Suitability for use as a treatment outcome measure has been established [6]. The recall period for the BPD checklist is one month.

Brief Symptom Inventory (BSI)

The BSI is a self-report instrument used as an inventory of general psychiatric symptoms present at the time of assessment and is a short alternative to the SCL-90-R, from which it was developed [7]. It contains 53 items to inventory the following nine types of primary symptom dimensions: somatic, cognitive, inter-personal sensitivity, depressive mood, anxiety, hostility, phobia, paranoia and psychoticism. Answers are scored on a 5-point Likert Scale. Cronbach's α is .96 for the instrument's total score [8]. In addition, the BSI has good discriminant validity [8]. The totals score of the BSI was used as an index of general psychiatric symptoms.

Happiness item

The happiness item is a single question on general happiness in the months prior to the assessment and is scored on a seven point Likert scale [9]. This scale consists of the following verbal descriptions of different states of happiness: (1) completely unhappy, (2) very unhappy, (3) fairly unhappy, (4) neither happy nor unhappy, (5) fairly happy, (6) very happy, (7) completely happy. Norms for all participating countries are available [9]. For a single happiness item high test-retest reliability ($r = 0.86$) and good concurrent, convergent, and divergent validity have been reported [10]. The happiness item has excellent sensitivity to change for patients with BPD who were treated with Group Schema Therapy [11].

© 2022 Arntz A et al. *JAMA Psychiatry*.

Schema Mode Inventory (SMI)

The SMI is a self-report instrument that consists of 143 items on 16 schema modes that are scored on six point Likert scales. It measures the extent to which dysfunctional as well as functional schema modes are present at the time of assessment [12]. It is an adaptation of the original SMI containing 270 items [13] and short SMI containing 118 items [14]. Its subscales have satisfactory to high internal consistency (Cronbach's α ranges from .79 to .96) [14]. We used a total score of the dysfunctional modes, and of the functional modes.

Young Schema Questionnaire – short form (YSQ)

The YSQ is a self-report instrument containing 75 items that are scored on a six point Likert scale [15]. It is used to measure the presence or absence of 16 core maladaptive schemas at the time of assessment. The YSQ has adequate temporal as well as rank-order stability and an analysis of its discriminant power in clinical versus non-clinical samples revealed it is highly sensitive in predicting the presence or absence of psychopathology [16]. Internal consistency is high for the overall scale (Cronbach's α ranges from .94 to .96) and satisfactory to high for its subscales (Cronbach's α ranges from .72 to .94) [17].

Global Assessment of Functioning (GAF) and Social and Occupational Functioning Assessment Scale (SOFAS)

Based on axis V of DSM-IV, the GAF and SOFAS are 100-point scales used to assess general and social/occupational functioning, respectively. A short semi-structured interview serves to elicit the information needed for scoring. The recall period for both instruments is one month. The GAF is a valid scale of global psychopathology and the SOFAS is a valid measure of social, occupational and interpersonal functioning [18]. Both instruments have excellent interrater reliability (intraclass correlation coefficients > .74) [18].

Work and Social Adjustment Scale (WSAS)

The WSAS is a self-report instrument that consists of 5 items that are scored on a scale ranging from 0 to 8. It is used to assess functional impairment at the time of assessment in the domains of work, household, social leisure, private leisure and family and relationships. The WSAS' reliability, validity and sensitivity to change have been firmly established in samples of patients with different clinical disorders [19-21].

World Health Organization Quality of Life questionnaire (WHOQOL-short)

The WHOQOL-short is a self-report instrument for assessing quality of life in the two weeks prior to assessment. It is a short version (35 items) of the WHOQOL and focuses on the domains of physical health, psychological health, social relationships, environment, positive feelings, negative feelings and self-esteem. The WHOQOL-short is a reliable and valid instrument [55].

* Note that this supplement is an adaptation of the section on clinical outcome measures in [23].

References

1. Arntz A, van den Hoorn M, Cornelis J, Verheul R, van den Bosch WM, de Bie AJ: Reliability and validity of the borderline personality disorder severity index. J Pers Disord 2003, 17(1):45–59.

© 2022 Arntz A et al. *JAMA Psychiatry*.

2. Giesen-Bloo J, Wachters LM, Schouten E, Arntz A: The borderline personality disorder severity index-IV: psychometric evaluation and dimensional structure. Pers Individ Differ 2010, 49:136–141.
3. Di Giacomo, E., Arntz, A., Fotiadou, M., Aguglia, E., Barone, L., Bellino, S., ... & Pinna, F. (2018). The Italian Version of the Borderline Personality Disorder Severity Index IV: Psychometric Properties, Clinical Usefulness, and Possible Diagnostic Implications. Journal of Personality Disorders, 32(2), 207-219.
4. Kröger, C., Vonau, M., Kliem, S., Röpke, S., Kosfelder, J. & Arntz, A. (2013). Psychometric properties of the German version of the Borderline Personality Disorder Severity Index - Version IV. Psychopathology, 46, 396-403. DOI:10.1159/000345404
5. Leppänen, V., Lindeman, S., Arntz, A., & Hakko, H. (2013). Preliminary evaluation of psychometric properties of the Finnish Borderline Personality Disorder Severity Index: Oulu-BPD-Study. Nordic Journal of Psychiatry, 67, 312-319. DOI: 10.3109/08039488.2012.745600
6. Bloo, J., Arntz, A., & Schouten, E. (2017). The Borderline Personality Disorder Checklist: Psychometric evaluation and factorial structure in clinical and nonclinical samples. Roczniki Psychologiczne, 20(2), 311-336.
7. Derogatis LR, Melisaratos N: The Brief Symptom Inventory: an introductory report. Psychol Med 1983, 13(3):595–605.
8. de Beurs E, Zitman F: The Brief Symptom Inventory (BSI): betrouwbaarheid en validiteit van een handzaam alternatief voor de SCL-90. Maandblad Geestelijke Volksgezondheid 2006, 61(2):120–137.
9. Veenhoven R: http://worlddatabaseofhappiness.eur.nl/hap_quer/hqs_fp.htm. 2008
10. Abdel-Khalek AM: Measuring happiness with a single-item scale. Soc Behav Pers Int J 2006, 34(2):139–150.
11. Dickhaut V, Arntz A: Combined group and individual schema therapy for borderline personality disorder: a pilot study. J Behav Ther Exp Psychiatry 2013, 45(2):242–251.
12. Lobbestael J, Van Vreeswijk MF, Arntz A: An empirical test of schema mode conceptualizations in personality disorders. Behav Res Ther 2008, 46(7):854–860.
13. Young JE, Arntz A, Atkinson T, Lobbestael J, Weishaar ME, van Vreeswijk MF, Klokman J: The Schema Mode Inventory. New York: Schema Therapy Institute; 2007.
14. Lobbestael J, van Vreeswijk M, Spinhoven P, Schouten E, Arntz A: Reliability and validity of the short Schema Mode Inventory (SMI). Behav Cogn Psychother 2010, 38(4):437–458.
15. Young JE: Young Schema Questionnaire Short Form. New York: Cognitive Therapy Centre; 1998.
16. Rijkeboer MM, van den Bergh H, van den Bout J: Stability and discriminative power of the Young Schema-Questionnaire in a Dutch clinical versus non-clinical population. J Behav Ther Exp Psychiatry 2005, 36(2):129–144.
17. Baranoff J, Oei TP, Cho SH, Kwon SM: Factor structure and internal consistency of the Young Schema Questionnaire (Short Form) in Korean and Australian samples. J Affect Disord 2006, 93(1–3):133–140.
18. Hilsenroth MJ, Ackerman SJ, Blagys MD, Baumann BD, Baity MR, Smith SR, Price JL, Smith CL, Heindselman TL, Mount MK, Holdwick DJ Jr: Reliability and validity of DSM-IV axis V. Am J Psychiatry 2000, 157(11):1858–1863.
19. Mundt JC, Marks IM, Shear MK, Greist JH: The work and social adjustment scale: a simple measure of impairment in functioning. Br J Psychiatry 2002, 180:461–464.
20. Mataix-Cols D, Cowley AJ, Hankins M, Schneider A, Bachofen M, Kenwright M, Gega L, Cameron R, Marks IM: Reliability and validity of the work and social adjustment scale in phobic disorders. Compr Psychiatry 2005, 46(3):223–228.

© 2022 Arntz A et al. *JAMA Psychiatry*.

21. Jansson-Frojmark M: The work and social adjustment scale as a measure of dysfunction in chronic insomnia: reliability and validity. Behav Cogn Psychother 2014, 4(42):186–198.
22. The WHOQOL Group: Development of the World Health Organization WHOQOL-BREF quality of life assessment. Psychol Med 1998, 28(3):551–558.
23. Wetzelaer P, et al.: Design of an international multicentre RCT on group schema therapy for borderline personality disorder. BMC Psychiatry 2014, 14:319.

© 2022 Arntz A et al. *JAMA Psychiatry*.

eAppendix 5. Overview of Random Effects for Secondary Outcomes.

Random effects of treatment for site and for cohort nested under site, and time for cohort nested under site were the default (3 random effects). If estimation failed, priority was given to the random effects for cohort under site, to account for group dependencies within sites. If none of these three random effects could be included, a random intercept for site was used. The specific random effects included in the model for a specific outcome are listed below.

Acceptability and safety

Treatment retention. Random effect of site. Models with random treatment effects for site or cohort nested under site did not converge.

Suicidality. Model with three random parts.

Suicide attempts. GEE used, GLMM not possible (no Tweedie distribution available in GLMM); random part NA.

BPDSI subscales

BPDSI Subscale 1 Abandonment. Two random effects: treatment for cohort nested under site, and time for cohort.

BPDSI Subscale 2 Interpersonal Relationships. Model with three random parts.

BPDSI Subscale 3 Identity. Two random effects: treatment for cohort nested under site, and time for cohort.

BPDSI Subscale 4 Impulsivity. Model with three random parts.

BPDSI Subscale 5 (Para)suicide. Two random effects: treatment for cohort nested under site, and time for cohort.

BPDSI Subscale 6 Affective Instability. Model with three random parts.

BPDSI Subscale 7 Emptiness. Model with three random parts.

BPDSI Subscale 8 Anger. Random effect of time for cohort.

BPDSI Subscale 9 Dissociation & Paranoia. Model with three random parts.

Other secondary outcomes

BPDCL, Happiness, WHOQOL, YSQ, SMI Dysfunctional, SMI Functional, WSAS. Two random effects: treatment for cohort nested under site, and time for cohort.

BSI and Work/Studying. Two random effects: treatment for site, and time for cohort.

© 2022 Arntz A et al. *JAMA Psychiatry*.

eAppendix 6. Estimation procedures for the effect size Cohen d and its 95% CI.

Following Morris (2008), the SD of the baseline was used a denominator for the estimation of Cohen's d. In short, the baseline SD has the least bias and is the most robust, and has a clear interpretation: it expresses the change in terms of the SD of the patient population before treatment. For the nominator the estimated (difference in) change according to the (G)LMM analyses was used (i.e., the beta's times the time over which the estimation was done). The estimated change is considered to be more robust than observed changes, as it is based on multilevel analysis taking into account the multiple sites, cohorts within sites, and missing values.

For GLMM analyses based on non-normal distributions using a loglink, for the denominator the square root of the baseline variance in the transformed scale as estimated by a GLMM on the baseline data with no random parts and only a fixed intercept was used. (adding random parts, e.g. for site, would influence the residual variance, hence lead to a biased estimation of the variance in the patient population). For the nominator the beta's of the analyses were used, that are in transformed scale. This way, both the nominator and the denominator are based on the transformed scale and more robust for skewed distributions than when assuming normal distributions.

For the 95% CI's of Cohen's d standard formulas are available (e.g., based on

$$SE = (n1+n2)/(n1n2)+d^2/(2(n1+n2), \qquad (1)$$

valid for n>20; see Goulet-Pelletier & Cousineau, 2018). However, using this to estimate the SE would ignore the multilevel structure in the data. The effect might be less homogeneous across sites than assumed in the formula. We had multiple sites and multiple cohorts, hence the effects estimated by the (G)LMM analyses are pooled effects across sites and cohorts and might have different variance than in case of a single sample. Thus, similar to a meta-analysis, the overall effect size might be relatively less precise than the effect size observed in a single sample.

To estimate valid 95% CI's for Cohen's d, we based them on the 95% CI's of the beta's of the (G)LMM analyses. In short, the beta's and the lower and upper values of their 95% CI's were multiplied by the appropriate time (i.e., 6 for 3 years, as time was coded in steps of 6 months). Then, these were divided by the square root of the baseline variance, estimated as explained above, yielding estimates for Cohen's d and its 95% CI. The estimates of the 95% CI were indeed larger than those based on the standard formula, as was expected given the multilevel design.

For piecewise regression we used the following approach. Time effects over 3 years are the result of the sum of the linear effect over 3 years and that over years 2 and 3. We estimated the change over 3 years as the combination of the two linear effects. Note that for estimation of the 2nd piece (years 2 and 3) the corresponding beta and 95% CI limits were multiplied by 4, and not 6, as the second piece of the regression is zero in year 1, after which it starts. Also note that for the treatment by time interactions, the time effect over 3 years does not contribute (this was deleted from the model as not contributing to explaining the effects). Hence, the effect size (and its 95% CI) was based on the second piece (year 2 and 3) beta of the interaction of time and treatment only.

The 95% CI of overall time effect for piecewise regression has two sources of variance: that of the linear slope over the full 3 years, and that of the linear slope over years 2 and 3. The estimation of the total SE was based on the following formula for the SE of a combination of variables:

© 2022 Arntz A et al. *JAMA Psychiatry*.

$$SE (6*Beta1 + 4*Beta2) = SQRT[(6*SE1)^2 + (4*SE2)^2 + 6*4*COV(Beta1,Beta2)]. \quad (2)$$

The SPSS LMM can deliver the covariances between beta's of the fixed part on request, this these were used. However, the GLMM module does not have that option. Hence, the correlation between the two beta's of the time effects as estimated by the LMM analyses on the natural log (LN) of the dependent variable was used to estimate the covariance:

$$COV(Beta1, Beta2) = R(Beta1, Beta2) * SE(Beta1) * SE(Beta2), \quad (3)$$

with the SE's from the GLMM analysis. (Checks on the sensitivity of the approach showed that the R(Beta1, Beta2) of the observed dependent variable of the LMM analysis was slightly higher (e.g., for WSAS -.824 vs -.715 for LN(WSAS)). Given the negative correlation, using the R based on untransformed dependent variable would lead to a sharper SE. Thus, for a more conservative estimation of the SE, the R of the LMM of the LN-transformed dependent variable was used.

References

Goulet-Pelletier, J.-C. & Cousineau, D. (2018). A review of effect sizes and their confidence intervals, Part I: The Cohen's d family. *The Quantitative Methods for Psychology, 14(4),* 242-265 . doi:10.20982/tqmp.14.4.p242.

Morris, S. B. (2008). Estimating effect sizes from pretestposttest-control group designs. *Organizational Research Methods, 11(2),* 364–386. doi:10.1177/1094428106291059

© 2022 Arntz A et al. *JAMA Psychiatry*.

eTable 3. Results of Loc-f sensitivity analysis.

The study protocol announced a Loc-f sensitivity analysis. Thus, the statistical analysis for the primary outcome was repeated after imputing missings after the last observation by means of the last-observation-carried-forward method. The results are summarized in the Table below. As can be seen, although effect sizes generally shrank, the conclusions were maintained (ST superior to TAU; IG-ST superior to the other arms, and PG-ST N.S. different from TAU.

BPDSI_Total_Loc-f	Time Effects (slope over 3 years)				
	t	df	p	d	r
Main Time Effect	12.69	34	<.001	1.51	**0.91**
Time x Treatment Comparison					
**ST vs TAU**	**2.14**	**1379**	**0.032**	**0.34**	**0.06**
PG-ST vs TAU	-0.27	1115	0.79	-0.05	0.01
**IG-ST vs TAU**	**3.60**	**1134**	**<.001**	**0.78**	**0.11**
**IG-ST vs Group-ST**	**3.02**	**478**	**0.003**	**0.73**	**0.14**

Note. With random effects of treatment and time for cohort nested under site.

© 2022 Arntz A et al. *JAMA Psychiatry*.

eTable 4. Sensitivity analysis of the zero-offset value for the gamma-regression of the BPDSI-total score.

ST vs TAU			
Gamma	TIME X TREATMENT		
OFFSET	t	p	
0.001	3.242	0.001	
0.01	3.241	0.001	used
0.05	3.234	0.001	
0.1	3.226	0.001	
0.5	3.165	0.002	

PG-ST, IG-ST, TAU							
Gamma	PG-ST vs TAU		IG-ST vs TAU		IG-ST vs PG-ST		
OFFSET	t	p	t	p	t	p	
0.001	0.988	0.323	3.950	<0.001	2.215	0.027	
0.01	0.988	0.323	3.948	<0.001	2.214	0.027	used
0.05	0.987	0.324	3.940	<0.001	2.209	0.028	
0.1	0.985	0.325	3.930	<0.001	2.203	0.028	
0.5	0.971	0.332	3.852	<0.001	2.158	0.031	

As can be seen, the variation in the offset value has only marginal influence on the results. In other words, the analysis is robust for the choice of the offset value.

© 2022 Arntz A et al. *JAMA Psychiatry*.

eTable 5. Descriptive statistics of observed BPDSI total scores, GLMM results in transformed scale, pairwise contrasts at 3 years (in original scale), treatment retention per 3 months by treatment arm

eTable 5.a. Descriptive statistics of observed BPDSI total scores.

	Treatment Arm														
	TAU					PG-ST					IG-ST				
	BPDSI_total					BPDSI_total					BPDSI_total				
Year	N	Median	Mean	Quartile 1	Quartile 3	N	Median	Mean	Quartile 1	Quartile 3	N	Median	Mean	Quartile 1	Quartile 3
0	246	31.56	31.63	25.32	37.44	125	29.62	30.96	25.12	35.80	123	29.57	30.44	23.73	36.13
0.5	184	27.60	26.71	19.33	33.80	97	24.95	25.30	19.05	30.46	105	25.44	25.69	17.60	32.51
1	151	22.47	23.18	14.87	30.96	85	20.26	21.54	14.96	27.24	99	22.56	22.14	13.98	28.95
1.5	138	20.13	21.67	13.09	29.81	74	20.00	19.96	12.61	25.36	86	17.76	19.01	12.33	25.84
2	140	17.77	19.15	9.65	26.86	75	15.93	16.67	11.29	22.82	83	17.46	17.08	9.90	23.38
3	132	15.18	16.55	7.17	23.21	67	12.26	14.35	6.38	19.38	78	10.32	12.68	4.05	20.62

eTable 5.b. Results of GLMM analysis of primary outcome (BPDSI-total score) in transformed scale.

	Estimated Means (95% CI), effect size													
Time Point	TAU			PG-ST			IG-ST				Time Effects (slope over 3 years)			
	M	95% CI	d^a	M	95% CI	d^a	M	95% CI	d^a		t	df	p	d^a	r
Baseline	3.42	3.35; 3.50		3.41	3.31; 3.50		3.42	3.33; 3.51		Main Time Effect	14.11	31	<.001	2.76	0.93
0.5 year	3.31	3.24; 3.39	0.40	3.28	3.19; 3.37	0.45	3.25	3.17; 3.34	0.59	Time x Treatment Comparison					
1 year	3.20	3.13; 3.28	0.80	3.16	3.06; 3.25	0.90	3.09	3.00; 3.18	1.18	**ST vs TAU**	**3.24**	**1028**	**0.001**	**0.73**	**0.10**
1.5 years	3.09	3.01; 3.17	1.20	3.03	2.93; 3.13	1.35	2.93	2.83; 3.03	1.77	PG-ST vs TAU	0.94	924	0.35	0.30	0.03
2 years	2.98	2.88; 3.08	1.60	2.91	2.79; 3.02	1.80	2.76	2.65; 2.88	2.36	**IG-ST vs TAU**	**3.99**	**916**	**<.001**	**1.14**	**0.14**
3 years	2.76	2.63; 2.89	2.41	2.66	2.50; 2.82	2.70	2.43	2.28; 2.59	3.55	**IG-ST vs PG-ST**	**2.28**	**491**	**0.023**	**0.84**	**0.08**

© 2022 Arntz A et al. *JAMA Psychiatry*.

Note. Analyzed with Generalized Linear Mixed Models gamma-regression with log-link, with random effects of treatment for site and of treatment and time for cohort (within site). Estimated means are in transformed scale. T-values and effect sizes d are positive when there is a positive effect (i.e., reduction over time, stronger reduction in ST than in TAU, etc.). ST represents the combined ST-arms. The main time effect is the average over ST and TAU. Significant treatment by time interactions are printed in bold.

[a] Effect sizes d are based on the parameters of the GLMM analyses (change over time), with the square-root of the baseline variance of a model with no random parts and only a fixed intercept, as denominator (i.e., SD baseline in transformed scale = 0.277). Estimated means are based on these parameters, for example effect size d for IG-ST at 3 years = (estimated mean at baseline – estimated mean at 3 year) / SD = (3.42-2.43)/0.277 = 3.568 (rounding errors lead to slightly different value than in the table (3.55).

[b] Effect sizes r are defined as $r = \sqrt{(t^2/(t^2 + df))}$, these represent the effect size associated with the effect tests in the fixed part of the GLMM.

eTable 5.c. Pairwise Contrasts BPDSI-total at year 3 between the 3 arms (in Original Scale)

Pairwise Contrasts	Contrast Estimate	Std. Error	t	df	p	95% Confidence Interval	
						Lower	Upper
ST-A - TAU	-1.514	1.179	-1.284	94	.202	-3.855	.828
ST-B - TAU	-4.335	1.013	-4.280	75	5.472E-5	-6.352	-2.317
ST-B - ST-A	-2.821	1.304	-2.163	192	.032	-5.392	-.249

Note. Based on the GLMM analysis used for the primary outcome analysis.

eTable 5.d. Treatment retention per 3 months by treatment arm

	TAU		PGST		IGST	
Quarter	N	proportion	N	proportion	N	proportion
0	246	1.000	125	1.000	123	1.000
1	216	0.878	109	0.872	114	0.928
2	200	0.813	97	0.775	107	0.871
3	187	0.759	93	0.743	106	0.863
4	180	0.730	90	0.718	100	0.815
5	170	0.689	86	0.686	94	0.766
6	166	0.673	85	0.678	92	0.750
7	163	0.661	82	0.653	92	0.750
8	158	0.640	78	0.621	91	0.741

© 2022 Arntz A et al. *JAMA Psychiatry*.

eTable 6. BPDSI-based secondary outcomes: BSPDI subscales, suicidality, suicide attempts.

	Estimated Means (95% CI), effect size														
Outcome and Time Point	TAU			PG-ST			IG-ST				Time Effects (slope over 3 years)				
	M	95% CI	d	M	95% CI	d	M	95% CI	d		t	df	p	d	r
BPDSI-Abandonment															
Baseline	2.87	2.59; 3.19		2.97	2.57; 3.43		3.05	2.64; 3.52		Main Time Effect	13.01	35	<.001	1.21	0.91
0.5 year	2.49	2.27; 2.74	0.18	2.49	2.19; 2.84	0.22	2.54	2.23; 2.89	0.23	Time x Treatment Comparison					
1 years	2.16	1.97; 2.37	0.36	2.09	1.83; 2.38	0.44	2.11	1.85; 2.40	0.46	**ST vs TAU**	**2.03**	**877**	**0.042**	**0.28**	**0.07**
1.5 years	1.87	1.69; 2.08	0.53	1.75	1.52; 2.02	0.66	1.75	1.53; 2.02	0.69	PG-ST vs TAU	1.41	702	0.16	0.25	0.05
2 years	1.62	1.44; 1.84	0.71	1.47	1.24; 1.73	0.88	1.46	1.24; 1.71	0.92	IG-ST vs TAU	1.81	716	0.07	0.31	0.07
3 years	1.22	1.03; 1.45	1.07	1.03	0.82; 1.30	1.32	1.01	0.81; 1.26	1.38	IG-ST vs PG-ST	0.27	278	0.79	0.06	0.02
BPDSI-Interpersonal Relationships															
Baseline	2.58	2.28; 2.91		2.34	2.01; 2.72		2.51	2.16; 2.92		Main Time Effect	12.51	37	<.001	1.29	0.90
0.5 year	2.25	2.00; 2.53	0.20	2.03	1.76; 2.34	0.21	2.14	1.86; 2.47	0.24	Time x Treatment Comparison					
1 years	1.97	1.76; 2.21	0.41	1.77	1.54; 2.04	0.43	1.83	1.59; 2.10	0.48	ST vs TAU	0.93	845	0.35	0.14	0.03
1.5 years	1.73	1.53; 1.95	0.61	1.54	1.32; 1.79	0.64	1.56	1.35; 1.81	0.72	PG-ST vs TAU	0.29	693	0.77	0.06	0.01
2 years	1.51	1.32; 1.73	0.81	1.34	1.13; 1.58	0.85	1.34	1.14; 1.57	0.96	IG-ST vs TAU	1.16	702	0.25	0.22	0.04
3 years	1.16	0.98; 1.37	1.22	1.01	0.79; 1.20	1.28	0.97	0.79; 1.20	1.44	IG-ST vs PG-ST	0.68	291	0.50	0.16	0.04
BPDSI-Identity															
Baseline	4.75	4.35; 5.20		4.62	4.08; 5.24		4.86	4.29; 5.50		Main Time Effect	14.39	35	<.001	2.40	0.92
0.5 year	4.06	3.46; 4.37	0.36	3.89	3.46; 4.37	0.39	3.89	3.46; 4.37	0.50	Time x Treatment Comparison					
1 years	3.47	3.18; 3.79	0.71	3.27	2.90; 3.69	0.78	3.12	2.77; 3.51	1.00	**ST vs TAU**	**2.77**	**898**	**0.006**	**0.57**	**0.09**
1.5 years	2.97	2.68; 3.29	1.07	2.75	2.41; 3.15	1.17	2.50	2.19; 2.85	1.51	PG-ST vs TAU	0.79	839	0.43	0.22	0.03
2 years	2.54	2.25; 2.86	1.42	2.32	1.98; 2.71	1.56	2.00	1.71; 2.33	2.01	**IG-ST vs TAU**	**3.36**	**836**	**0.001**	**0.88**	**0.12**
3 years	1.85	1.56; 2.20	2.13	1.64	1.32; 2.04	2.34	1.28	1.04; 1.59	3.01	IG-ST vs PG-ST	1.94	466	0.054	0.66	0.09
BPDSI-Impulsivity															
Baseline	1.43	1.19; 1.72		1.29	1.02; 1.62		1.37	1.09; 1.72		Main Time Effect	10.67	31	<.001	0.76	0.89
0.5 year	1.29	1.07; 1.54	0.11	1.15	0.92; 1.43	0.12	1.16	0.93; 1.45	0.17	Time x Treatment Comparison					

© 2022 Arntz A et al. *JAMA Psychiatry*.

1 years	1.15	0.96; 1.38	0.22		1.02	0.82; 1.28	0.23		0.99	0.79; 1.23	0.33		ST vs TAU	1.59	681	0.11	0.18	0.06
1.5 years	1.04	0.86; 1.25	0.33		0.91	0.73; 1.15	0.35		0.84	0.67; 1.05	0.50		PG-ST vs TAU	0.26	534	0.80	0.04	0.01
2 years	0.93	0.77; 1.13	0.44		0.81	0.64; 1.04	0.47		0.71	0.56; 0.90	0.67		**IG-ST vs TAU**	**2.44**	**540**	**0.015**	**0.34**	**0.10**
3 years	0.75	0.60; 0.93	0.66		0.65	0.49; 0.86	0.70		0.51	0.39; 0.68	1.00		IG-ST vs PG-ST	1.73	196	0.09	0.30	0.12
BPDSI-(Para)Suicide																		
Baseline	0.83	0.68; 1.01			0.84	0.64; 1.12			0.77	0.58; 1.02			Main Time Effect	8.64	31	<.001	0.72	0.84
0.5 year	0.73	0.60; 0.88	0.10		0.73	0.55; 0.95	0.11		0.61	0.46; 0.80	0.18		Time x Treatment Comparison					
1 years	0.64	0.53; 0.78	0.20		0.63	0.48; 0.83	0.23		0.48	0.37; 0.63	0.37		**ST vs TAU**	**2.47**	**590**	**0.014**	**0.30**	**0.10**
1.5 years	0.56	0.46; 0.70	0.30		0.54	0.41; 0.73	0.34		0.38	0.28; 0.51	0.55		PG-ST vs TAU	0.55	505	0.59	0.09	0.02
2 years	0.50	0.39; 0.63	0.40		0.47	0.34; 0.65	0.46		0.30	0.22; 0.41	0.73		**IG-ST vs TAU**	**3.28**	**499**	**0.001**	**0.50**	**0.15**
3 years	0.38	0.28; 0.52	0.60		0.35	0.23; 0.52	0.68		0.19	0.13; 0.28	1.10		**IG-ST vs PG-ST**	**2.21**	**251**	**0.028**	**0.41**	**0.14**
BPDSI-Affective Instability																		
Baseline	7.06	6.55; 7.61			7.02	6.40; 7.71			6.91	6.31; 7.58			Main Time Effect	11.49	33	<.001	1.89	0.89
0.5 year	6.51	6.05; 6.99	0.27		6.37	5.84; 6.95	0.32		6.09	5.59; 6.64	0.41		Time x Treatment Comparison					
1 years	5.99	5.56; 6.45	0.53		5.78	5.29; 6.32	0.64		5.37	4.92; 5.86	0.82		**ST vs TAU**	**2.91**	**1001**	**0.004**	**0.60**	**0.09**
1.5 years	5.52	5.09; 5.98	0.80		5.24	4.75; 5.78	0.96		4.73	4.30; 5.21	1.24		PG-ST vs TAU	1.11	912	0.27	0.30	0.04
2 years	5.09	4.64; 5.57	1.07		4.76	4.25; 5.32	1.27		4.17	3.74; 4.65	1.65		**IG-ST vs TAU**	**3.34**	**908**	**0.001**	**0.87**	**0.11**
3 years	4.32	3.82; 4.87	1.60		3.92	3.36; 4.56	1.91		3.24	2.80; 3.75	2.47		IG-ST vs PG-ST	1.68	478	0.09	0.57	0.08
BPDSI-Emptiness																		
Baseline	6.11	5.60; 6.66			5.71	5.10; 6.38			6.03	5.10; 6.38			Main Time Effect	12.34	32	<.001	1.43	0.91
0.5 year	5.47	5.04; 5.94	0.23		5.15	4.64; 5.71	0.21		5.20	4.69; 5.77	0.30		Time x Treatment Comparison					
1 years	4.90	4.51; 5.32	0.45		4.64	4.18; 5.16	0.42		4.49	4.05; 4.98	0.60		ST vs TAU	1.13	825	0.260	0.18	0.04
1.5 years	4.39	4.01; 4.80	0.68		4.19	3.73; 4.70	0.63		3.88	3.47; 4.34	0.90		PG-ST vs TAU	-0.40	702	0.692	-0.08	0.02
2 years	3.93	3.55; 4.35	0.90		3.78	3.31; 4.31	0.84		3.35	2.95; 3.80	1.20		**IG-ST vs TAU**	**2.20**	**705**	**0.028**	**0.45**	**0.08**
3 years	3.15	2.75; 3.62	1.35		3.07	2.57; 3.67	1.26		2.50	2.11; 2.96	1.80		**IG-ST vs PG-ST**	**2.04**	**318**	**0.042**	**0.54**	**0.11**
BPDSI-Anger																		
Baseline	2.71	2.46; 2.99			2.87	2.51; 3.29			2.54	2.21; 2.91			Main Time Effect	10.89	39	<.001	0.93	0.87
0.5 year	2.43	2.23; 2.66	0.12		2.48	2.18; 2.80	0.17		2.10	1.86; 2.38	0.21		Time x Treatment Comparison					

© 2022 Arntz A et al. *JAMA Psychiatry*.

1 years	2.19	1.99; 2.40	0.24		2.13	1.88; 2.42	0.34		1.75	1.54; 1.98	0.42		**ST vs TAU**	**3.49**	**732**	**<.001**	**0.42**	**0.13**
1.5 years	1.96	1.77; 2.18	0.37		1.84	1.60; 2.11	0.51		1.45	1.26; 1.66	0.63		PG-ST vs TAU	1.79	668	0.074	0.28	0.07
2 years	1.76	1.56; 1.99	0.49		1.58	1.34; 1.87	0.67		1.20	1.02; 1.41	0.84		**IG-ST vs TAU**	**3.59**	**664**	**<.001**	**0.54**	**0.14**
3 years	1.42	1.20; 1.67	0.73		1.17	0.93; 1.48	1.01		0.83	0.66; 1.03	1.26		IG-ST vs PG-ST	1.34	347	0.182	0.26	0.07
BPDSI-Dissociation/Paranoia																		
Baseline	2.24	1.88; 2.68			2.24	1.81; 2.78			2.26	1.83; 2.80			Main Time Effect	7.57	31	<.001	0.91	0.81
0.5 year	1.98	1.66; 2.35	0.13		1.94	1.58; 2.38	0.15		1.84	1.50; 2.26	0.21		Time x Treatment Comparison					
1 years	1.75	1.46; 2.09	0.25		1.67	1.35; 2.07	0.30		1.50	1.22; 1.85	0.41		**ST vs TAU**	**2.50**	**612**	**0.013**	**0.33**	**0.10**
1.5 years	1.55	1.27; 1.88	0.38		1.44	1.14; 1.82	0.45		1.23	0.98; 1.54	0.62		PG-ST vs TAU	0.81	601	0.42	0.14	0.03
2 years	1.37	1.10; 1.70	0.51		1.25	0.96; 1.63	0.60		1.00	0.77; 1.30	0.83		**IG-ST vs TAU**	**2.92**	**581**	**0.004**	**0.49**	**0.12**
3 years	1.07	0.80; 1.42	0.76		0.93	0.66; 1.32	0.90		0.67	0.48; 0.93	1.24		IG-ST vs PG-ST	1.56	398	0.12	0.35	0.08
**Suicidality**																		
BPDSI-Suicidality Items (sumscore)																		
Baseline	1.12	0.87; 1.44			1.31	0.96; 1.79			1.13	0.83; 1.54			Main Time Effect	9.89	29	<.001	0.72	0.88
0.5 year	0.97	0.82; 1.43	0.07		1.07	0.79; 1.43	0.13		0.84	0.63; 1.12	0.15		Time x Treatment Comparison					
1 years	0.85	0.66; 1.08	0.15		0.87	0.65; 1.17	0.27		0.62	0.47; 0.83	0.31		**ST vs TAU**	**3.43**	**511**	**<.001**	**0.43**	**0.15**
1.5 years	0.73	0.57; 0.95	0.22		0.74	0.57; 0.97	0.40		0.46	0.34; 0.63	0.46		PG-ST vs TAU	1.55	439	0.12	0.26	0.07
2 years	0.64	0.48; 0.84	0.30		0.58	0.41; 0.82	0.54		0.34	0.24; 0.48	0.61		**IG-ST vs TAU**	**3.93**	**437**	**<.001**	**0.63**	**0.18**
3 years	0.48	0.34; 0.68	0.56		0.38	0.24; 0.60	0.82		0.19	0.12; 0.29	1.19		IG-ST vs PG-ST	1.80	223	0.07	0.37	0.12
BPDSI-Suicide Attempts (number per 3 months)														Wald	df	p	d	r
Baseline	0.113	0.069; 0.183			0.083	0.034; 0.200			0.140	0.058; 0.337			Main Time Effect	5.91	1	0.015	0.24	0.11
0.5 year	0.101	0.061; 0.169	0.02		0.069	0.035; 0.135	0.04		0.089	0.040; 0.199	0.09		Time x Treatment Comparison					
1 years	0.091	0.050; 0.167	0.04		0.057	0.031; 0.108	0.07		0.057	0.025; 0.129	0.17		ST vs TAU	1.26	1	0.26	0.24	0.05
1.5 years	0.082	0.039; 0.170	0.06		0.048	0.022; 0.104	0.10		0.036	0.015; 0.091	0.25		PG-ST vs TAU	0.14	1	0.71	0.09	0.02
2 years	0.074	0.031; 0.178	0.08		0.040	0.014; 0.112	0.14		0.023	0.008; 0.068	0.34		**IG-ST vs TAU**	**4.06**	**1**	**0.044**	**0.39**	**0.09**
3 years	0.060	0.018; 0.200	0.12		0.028	0.005; 0.147	0.21		0.010	0.002; 0.042	0.51		IG-ST vs PG-ST	1.29	1	0.26	0.30	0.05

© 2022 Arntz A et al. *JAMA Psychiatry*.

Note. Estimated means are in original scale. Effect sizes d are based on the parameters of the GLMM analyses (change over time), with the square-root of the baseline variance of a model with no random parts and only a fixed intercept, as denominator. Effect sizes r are defined as $r = \sqrt{t^2/(t^2 + d.f.)}$, these represent the effect size associated with the effect tests in the fixed part of the GLMM. T-values and d are positive when there is a positive effect (i.e., reduction over time, superior reduction in ST than in TAU, etc.). ST represents the combined ST-arms. The main time effect is the average over ST and TAU. Significant treatment by time interactions are printed in bold.

© 2022 Arntz A et al. *JAMA Psychiatry*.
