# Supplementary material for: Effectiveness of Predominantly Group Schema Therapy and Combined Individual and Group Schema Therapy for Borderline Personality Disorder: A Randomized Clinical Trial
Source: JAMA Psychiatry. 2022 Mar 2;79(4):287–99. doi: 10.1001/jamapsychiatry.2022.0010 (PMC8892362; doi:10.1001/jamapsychiatry.2022.0010)
Supplement: Supplement 3. — Data Sharing Statement [file jamapsychiatry-e220010-s003.pdf]

Data Sharing Statement

Arntz. Effectiveness of Predominantly Group Schema Therapy and Combined Individual and Group Schema Therapy for Borderline Personality Disorder. *JAMA Psychiatry*. Published March 02, 2022. doi:10.1001/jamapsychiatry.2022.0010

Data
Data available: Yes
Data types: Deidentified participant data
How to access data: Pseudonymised individual participant data that underlie the results reported in this article will be made available for researchers upon reasonable request, which must include a protocol and statistical analysis plan and not be in conflict with our prespecified publication plan. The request should include a guarantee of compliance with the EU and participating countries' General Data Protection Regulations given the privacy sensitive character of the data. Data and a data dictionary will be made available after publication, for at least 5 years. Requests for data sharing will be considered by the study board. Requests should be directed to a.r.arntz@uva.nl; requestors will need to sign a data access agreement.
When available: With publication

Supporting Documents
Document types: Statistical/analytic code, Informed consent form
How to access documents: a.r.arntz@uva.nl
When available: With publication

Additional Information
Who can access the data: Pseudonymised individual participant data that underlie the results reported in this article will be made available for researchers upon reasonable request, which must include a protocol and statistical analysis plan and not be in conflict with our prespecified publication plan. The request should include a guarantee of compliance with the EU and participating countries' General Data Protection Regulations given the privacy sensitive character of the data. Data and a data dictionary will be made available after publication, for at least 5 years. Requests for data sharing will be considered by the study board. Requests should be directed to a.r.arntz@uva.nl; requestors will need to sign a data access agreement.
Types of analyses: Pseudonymised individual participant data that underlie the results reported in this article will be made available for researchers upon reasonable request, which must include a protocol and statistical analysis plan and not be in conflict with our prespecified publication plan. The request should include a guarantee of compliance with the EU and participating countries' General Data Protection Regulations given the privacy sensitive character of the data. Data and a data dictionary will be made available after publication, for at least 5 years. Requests for data sharing will be considered by the study board. Requests should be directed to a.r.arntz@uva.nl; requestors will need to sign a data access agreement.
Mechanisms of data availability: Pseudonymised individual participant data that underlie the results reported in this article will be made available for researchers upon reasonable request, which must include a protocol and statistical analysis plan and not be in conflict with our prespecified publication plan. The request should include a guarantee of compliance with the EU and participating countries' General Data Protection Regulations given the privacy sensitive character of the data. Data and a data dictionary will be made available after publication, for at least 5 years. Requests for data sharing will be considered by the study board. Requests should be directed to a.r.arntz@uva.nl; requestors will need to sign a data access agreement.
Any additional restrictions: Pseudonymised individual participant data that underlie the results reported in this article will be made available for researchers upon reasonable request, which must include a protocol and statistical analysis plan and not be in conflict with our prespecified publication plan. The request should include a guarantee of compliance with the

EU and participating countries' General Data Protection Regulations given the privacy sensitive character of the data. Data and a data dictionary will be made available after publication, for at least 5 years. Requests for data sharing will be considered by the study board. Requests should be directed to a.r.arntz@uva.nl; requestors will need to sign a data access agreement.
